# Structure and Methylation of 35S rDNA in Allopolyploids *Anemone multifida* (2*n* = 4*x* = 32, BBDD) and *Anemone baldensis* (2*n* = 6*x* = 48, AABBDD) and Their Parental Species Show Evidence of Nucleolar Dominance

**DOI:** 10.3389/fpls.2022.908218

**Published:** 2022-07-06

**Authors:** Jelena Mlinarec, Ljudevit Luka Boštjančić, Nenad Malenica, Adela Jurković, Todd Boland, Sonja Siljak Yakovlev, Višnja Besendorfer

**Affiliations:** ^1^Oikon ltd.-Institute of Applied Ecology, Zagreb, Croatia; ^2^LOEWE Centre for Translational Biodiversity Genomics (LOEWE-TBG), Senckenberg Biodiversity and Climate Research Centre, Senckenberg Gesellschaft für Naturforschung, Frankfurt, Germany; ^3^Department of Computer Science, ICube, UMR 7357, CNRS, Centre de Recherche en Biomédecine de Strasbourg, University of Strasbourg, Strasbourg, France; ^4^Division of Molecular Biology, Department of Biology, University of Zagreb, Horvatovac, Croatia; ^5^Memorial University of Newfoundland’s Botanical Gardens, St. John’s, NL, Canada; ^6^CNRS, AgroParisTech, Ecologie Systématique Evolution, Université Paris-Saclay, Orsay, France

**Keywords:** bisulfite sequencing, CpG islands, genome size, epigenetic modification, IGS, Ranunculaceae, silver staining, polyploidy

## Abstract

Transcriptional silencing of 35S rDNA loci inherited from one parental species is occurring relatively frequently in allopolyploids. However, molecular mechanisms by which it is selected for transcriptional silencing remain unclear. We applied NGS, silver staining and bisulfite sequencing to study the structure, expression and methylation landscape of 35S rDNA in two allopolyploids of common origin, allotetraploid *Anemone multifida* (2*n* = 4*x* = 32, genome composition BBDD) and allohexaploid *A. baldensis* (2*n* = 6*x* = 48, AABBDD), and their genome donors, *A. sylvestris* (2*n* = 16, AA), *A. cylindrica* (2*n* = 16, BB) and *A. parviflora* (2*n* = 16, DD). The size of the recovered 35S rDNA units varied from 10,489 bp in *A. cylindrica* to 12,084 bp in *A. sylvestris*. *Anemone* showed an organization typical of most ribosomal 35S rDNA composed of NTS, ETS, rRNA genes, TTS and TIS with structural features of plant IGS sequences and all functional elements needed for rRNA gene activity. The NTS was more variable than the ETS and consisted of SRs which are highly variable among *Anemone*. Five to six CpG-rich islands were found within the ETS. CpG island located adjacent to the transcription initiation site (TIS) was highly variable regarding the sequence size and methylation level and exhibited in most of the species lower levels of methylation than CpG islands located adjacent to the 18S rRNA gene. Our results uncover hypomethylation of *A. sylvestris*- and *A. parviflora*-derived 35S rDNA units in allopolyploids *A. multifida* and *A. baldensis*. Hypomethylation of *A. parviflora*-derived 35S rDNA was more prominent in *A. baldensis* than in *A. multifida*. We showed that *A. baldensis* underwent coupled *A. sylvestris*-derived 35S rDNA array expansion and *A. parviflora*-derived 35S rDNA copy number decrease that was accompanied by lower methylation level of *A. sylvestris*-derived 35S rDNA units in comparison to *A. parviflora*-derived 35S rDNA units. These observations suggest that in *A. baldensis* nucleolar dominance is directed toward *A. sylvestris*-derived chromosomes. This work broadens our current knowledge of the 35S rDNA organization in *Anemone* and provides evidence of the progenitor-specific 35S rDNA methylation in nucleolar dominance.

## Introduction

In eukaryotes, the 35S rDNA is arranged as highly repeated tandems and located at one or more chromosomal loci (Garcia et al., 2017). The rDNA unit is composed of the coding region for the 18S, 5.8S and 26S rRNA genes, the internal transcribed spacers (ITS1 and ITS2) and the intergenic spacer (IGS). IGS is composed of the 3′ external spacer (3′-ETS), the non-transcribed region (NTS), and the 5′ external transcribed spacer (5′-ETS). The 35S rDNA in plants is highly variable in copy number and in the nucleotide composition and length of IGS, however, the functional role of this region is relatively conservative (Rogers and Bendich, 1987). Repetitive elements (subrepeats), transcription initiation (TIS) and termination sites (TTS) can be found within IGS, functioning as enhancers, sequences with self-complementarity that could generate a conserved secondary structure, which are involved in the regulation of transcription of the 18-5.8-26S rDNA cistrons. Only 35S rDNA loci with active rRNA transcription and processing can form a nucleolus during the interphase of each cell cycle, and therefore, can be named as nucleolar organizer regions (NORs) (Ritossa and Spiegelman, 1965).

DNA methylation is an epigenetic mark that plays an essential role in regulating gene expression. DNA methylation is known to occur in all cytosine contexts (CpG, CpNpG, CpNpN) (Lawrence et al., 2004). CpG islands are regions rich in CpG, CpNpG, CpNpN di- and tri-nucleotides, no smaller than 200 bp and mostly present in promoters. The DNA methylation of CpG islands regulate gene expression through transcriptional silencing of the corresponding gene (Takai and Jones, 2002; Grummt and Pikaard, 2003; Lim et al., 2019). In 35S rDNA, the IGS is a region involved in transcriptional regulation, therefore, contains many pyrimidine base-pairs as methylation sites (Inácio et al., 2014). The enzymatic addition of a methyl group to DNA is performed by DNA methyltransferase (DNMT) on the 5′-carbon of the pyrimidine ring in cytosine. It is well known that the methylation in the promoter region represses the rRNA gene expression in NORs (Grummt and Pikaard, 2003). Due to the presence of the ribosomal genes in many copies, only a small amount of these genes is transcriptionally active at any given time. The remaining of rDNA units bears epigenetic modifications, characteristic of heterochromatin, keeping it transcriptionally silent (Vieira et al., 1990; Neves et al., 1995; Costa-Nunes et al., 2010).

Allopolyploidy, interspecific hybridization followed by chromosome doubling, is one of the major driving forces in plant evolution (Liu and Wendel, 2003). The merger of two or more divergent genomes within one nucleus is challenging regarding long-term viability, and the sub-genomes undergo structural changes such as deletions, translocations, transposon activation and meiotic irregularities (Mlinarec et al., 2012a). Besides, subgenomes may undergo the reversible silencing of one of the NOR-bearing homoeologues, phenomenon termed as Nucleolar Dominance (ND) (Leitch and Leitch, 2008; Weiss-Schneeweiss et al., 2013; Wendel et al., 2018). Although our understanding of the role of DNA methylation in establishing and maintaining the ND has increased significantly in the last decades (Chen and Pikaard, 1997; Lawrence et al., 2004; Earley et al., 2006; Costa-Nunes et al., 2010), the mechanisms by which one ancestral rDNA set is selected for silencing remains elusive (Symonova, 2019). It was shown that in *Arabidopsis thaliana* and *Hordeum vulgare*, the 35S rRNA genes were silenced based on the chromosomal position in which they resided (Nicoloff et al., 1979; Schubert and Kunzel, 1990; Mohannath et al., 2016). In the allotetraploid *Arabidopsis suecica*, the small interfering RNA (siRNA)-directed DNA methylation pathway is required to inactivate the 35S rDNA of an *A. thaliana* origin (Preuss et al., 2008).

*Anemone* sensu stricto (s.s.) (c. 150 species, basic chromosome number *x* = 8), consists of perennial, low-growing herbs of worldwide distribution and with considerable diversity in morphology (Tamura, 1995). Various studies in the genus *Anemone* used the variation in the internal transcribed spacers (ITS1 and ITS2) of the 35S rDNA and the 5S rDNA intergenic spacers (NTS) mainly for systematic and phylogenetic purposes (Meyer et al., 2010; Mlinarec et al., 2016). However, the sequence structure and the methylation landscape of the 35S rDNA has never been studied in *Anemone*.

The genus *Anemone* offers an interesting plant model in studies of 35S rDNA evolution and molecular mechanisms of silencing of ancestral rDNA set in allopolyploids. *Anemone multifida* (2*n* = 4*x* = 32, BBDD) and *A. baldensis* (2*n* = 6*x* = 48, AABBDD) are allopolyploids of hybrid origin with *A. sylvestris* (2*n* = 16, AA), *A. cylindrica* (2*n* = 16, BB) and *A. parviflora* (2*n* = 16, DD) as their genome donors (Mlinarec et al., 2012a,^2016^). Allotetraploid *A. multifida* originated from the cross of the species the most similar to *A. cylindrica* (North America, moderate climate) and *A. parviflora* (North America, arctic climate), while allohexaploid *A. baldensis* (Europe, mountainous climate) originated from the cross of *A. multifida* (North and South America, mountainous climate) and *A. sylvestris* (Northern Europe and Asia, moderate climate). In *A. multifida* and *A. baldensis*, a uniparental/biparental 35S rDNA inheritance from the D subgenome donor (*A. multifida*) and the A and D subgenome donor (*A. baldensis*) has occurred as a result of the complete elimination of the rDNA units from the B subgenome donor (Mlinarec et al., 2012a). *Anemone sylvestris* and *A. cylindrica* have two 35S rDNA loci positioned terminally on short arm of two acrocentric chromosome pairs, while allopolyploids *A. multifida* and *A. baldensis* possess four and eight 35S rDNA signals, respectively. In *A. multifida*, these four 35S rDNA sites are located terminally in the D subgenome, while in *A. baldensis*, four 35S rDNA sites are located both in the A subgenome and the D subgenome (Mlinarec et al., 2012a,b).

Bisulfite-sequencing is an efficient method to determine 5-methylcytosine (5-mC) content at single-base resolution. It is based on sodium-bisulfite modification reaction which deaminates cytosine (but not the 5-mC) to uracil (U). The result is the conversion of C to thymine (T) in DNA during the synthesis of the complementary strand allowing the discrimination of unmethylated cytosine (C) from 5-mC. Presence of C in the sequencing read indicates that the cytosine was methylated, as the 5-mC is protected during sodium bisulfite reaction.

In order to shed more light on the 35S rDNA evolution and molecular mechanisms that lie behind epigenomic regulation in allopolyploid, we applied next generation sequencing (NGS), silver staining and bisulfite sequencing to study the organization and DNA methylation landscape of 35S rDNA in *Anemone* allopolyploids and their progenitor species. The following questions were posed: (*i)* what is the molecular organization of 35S rDNA in allopolyploids *A. multifida* (2*n* = 4*x* = 32, BBDD) and *A. baldensis* (2*n* = 6*x* = 48, AABBDD) and their parental species *A. sylvestris* (2*n* = 16, AA), *A. cylindrica* (2*n* = 16, BB) and *A. parviflora* (2*n* = 16, DD)?, (*ii)* is the methylation landscape uniform or is it changing depending on the region within the ETS analyzed? and (*iii)* is DNA methylation involved in the preferential suppression of the homoeologous rDNA units?

## Materials and Methods

### Samples and DNA Extraction

*Anemone parviflora* was obtained from the wild from Canada, Flower’s Cove, Newfoundland (voucher number: UNB60590), while *A. sylvestris* (voucher number: ZA40577), *A. cylindrica* (voucher number: ZA40583), *A. multifida* (voucher number: ZA40585) and *A. baldensis* (voucher number: ZA40571) were grown in pots in Zagreb, Botanical Garden of University of Zagreb (Table 1).

**TABLE 1:** List of *Anemone* species studied, their voucher number, origin and colection data.

Species	Voucher number	Origin
*A. baldensis*	ZA40571	Garden material from Botanical Garden of University of Zagreb, Croatia
*A. cylindrica*	ZA40583	Seed from Botanical Garden Chemnitz, Germany, Botanical garden University of Zagreb, Croatia
*A. parviflora*	UNB60590	Flower’s Cove, Newfoundland, Canada, collected by T. Boland
*A. multifida*	ZA40585	Wenatchee Mountains, Washington State, United States, purchased from Kevock Garden, United Kingdom, Botanical garden University of zagreb
*A. sylvestris*	ZA40577	Čučerje, Medvednica, Croatia, Botanical Garden of the University of Zagreb

Fresh leaves of *Anemone sylvestris, A. cylindrica, A. multifida* and *A. baldensis* and silica dried *A. parviflora* leaves were used for DNA extraction. Care was taken to collect young light green leaves from all plants. Plants were in the period of developing flower buds. Total genomic DNA (gDNA) was isolated from ∼100 mg of pooled leaves using a commercial plant gDNA isolation kit (DNeasy^®^ Plant Mini Kit - QIAGEN) according to the manufacturer’s instructions. The amount and purity of isolated gDNA was determined by using the NanoVue spectrophotometer (GE, Healthcare, United States).

### Silver Staining and Genomic *in situ* Hybridization

Nuclei and metaphase chromosomes from the root tip cells were used for determination of active Nucleolar Organizing Regions (NORs). Metaphase chromosome spreads were obtained according to Mlinarec et al. (2006). Silver staining was performed according to the method of Hizume et al. (1980). Three individuals were analyzed from each species. In total, from 448 to 536 cells were analyzed per each species (Supplementary Table 1). We could not analyze the nucleoli of *A. parviflora* due to lack of living plants.

### Flow Cytometry Analysis

The 2C nuclear DNA content was assessed by flow cytometry according to methods described by Marie and Brown (1993) and Mlinarec et al. (2009). *Triticum aestivum* cv. Chinese spring (2C = 30.9 pg and 43.7% G) was used as an internal standard. A slightly modified Galbraith’s nuclei isolation buffer (Galbraith et al., 1983): 45 mM MgCl2, 30 mM sodium citrate, 60 mM MOPS (4-morpholine propane sulphonate, pH = 7), and 1% (w/v) polyvinylpyrrolidone 10,000, pH 7.2) containing 0.1% (w/v) Triton X–100, supplemented with fresh 5 mM sodium metabisulphite and RNase (2.5 U/ml), was used for nuclei isolation and nuclei were stained with 100 μg/mL propidium iodide (Sigma Chemical Co., St. Louis, United States), the DNA intercalating fluorochrome dye. The nuclei suspension was filtered through 48 μm nylon mesh. To obtain the mean DNA content, six *Anemone multifida* and *A. baldensis* individuals were measured separately and with repetition on a flow cytometer (CyFlow SL3, Partec, Munster, Germany) with a 532 nm laser. At least 5,000 to 10,000 nuclei were analyzed for each of six samples. The 2C DNA value was calculated using the linear relationship between the fluorescent signals from stained nuclei of the *A. multifida* and *A. baldensis* specimen and the *Triticum aestivum* internal standard.

### Illumina NextSeq Sequencing and Clustering of Genomic Reads

A total of 1.25-17 μg of high molecular weight (> 20 kb) genomic DNA from *A. parviflora*, *A. cylindrica*, *A. sylvestris*, *A. multifida* and *A. baldensis* was all sequenced using the Illumina HiSeq X platform (375 × 10^6^ reads/lane, library insert size 350 bp) with a total 50 Gbp output for all five species. Raw Illumina 150 bp pair-end reads from low coverage DNA-seq experiments were obtained from a commercial service (Macrogen, Netherlands). Raw Illumina reads from DNA-seq experiment are available from SRA-NCBI under the BioProject ID PRJNA830872. Sequencing coverage, genome size, read archive accessions and other parameters are shown in Table 2.

**TABLE 2:** Basic statistics of NGS carried out in this study and copy number of the 35S rDNA units in allopolyploids *Anemone multifida* and *Anemone baldensis* and their ancestral species.

Species	1C [pg]	1C [Mb]	Sequencing coverage^a^	GC [%]	Read archive accession	Number of mapped reads to 26S rDNA loci	Coverage 26S rDNA^b^	Total number of reads used for mapping^c^	Genome coverage of reads used for mapping^d^	Number of 35S rDNA copies per 1C^e^	Subgenome	Subgenomic copy number of 35S rDNA per 1C^h^
*Anemone parviflora*	5.9^f^	5,770.20	9.87	39	SRR18890536	2,51,920	9,556.02	17,10,09,442	3.85	2,480		
*Anemone cylindrica*	9.3^f^	9,095.40	17.37	38	SRR18890535	4,14,074	15,565.30	78,86,75,298	11.27	1,381		
*Anemone sylvestris*	8.06^g^	7,882.68	15.95	38	SRR18890538	6,14,468	23,196.44	65,50,48,152	10.8	2,147		
*Anemone multifida*	15.25	14,914.50	3.56	39	SRR18890539	1,22,614	4,650.04	27,83,53,314	2.43	1,917		
*Anemone baldensis*	21.01	20,542.89	2.73	38	SRR18890537	2,47,230	9,375.60	29,21,94,250	1.85	5,070	AA	2,934
											DD	2,136

^a^Calculated as follows: Number of Reads * Average Read length (bp) */Genome size (Mb) *106, with an average read length of 150 bp.

*^b^The length of 26S rDNA sequence that was used as a reference is 3402 bp.*

*^c^Reads that mapped to the chloroplast genome were excluded from analysis.*

^d^Calculated as follows: Number of Reads * Average Read length (bp) */Genome size (Mb) *106.

^e^Calculated as follows: Coverage of 26S rDNA */Genome coverage of reads used for mapping.

*^f^From Rothfels et al. (1966).*

*^g^From Veselý et al. (2013).*

*^h^Calculated from the coverage of IGS subgenomic variants which is 55.2x (Subgenome A): 40.2 x (Subgenome D) (see Figure 2).*

### Reconstruction of the Ribosomal DNA Array

Three to five million pair end reads from each genome were subjected to clustering analysis using RepeatExplorer and TAREAN pipeline (Novák et al., 2013; Boštjančić et al., 2021). Clusters containing 35S rDNA sequences were manually identified and confirmed with the BLASTn search against sequences deposited in GeneBank. These partial ribosomal DNA arrays were used as baits for the reconstruction of the full-length ribosomal DNA array loci with GetOrganelle 1.7.3.4 (Jin et al., 2020) and recommended parameters for reconstruction of plant ribosomal DNA arrays: -R 10 -k 35,85,115 -F embplant-nr. To confirm the correct identification of ribosomal DNA array assembly graphs were visualized with Bandage 0.8.1 (Wick et al., 2015). Boundaries of each coding region were determined from the alignment of *Anemone* ribosomal DNA arrays with the 35S rDNA sequence of *Alnus jorullensis* subsp. *jorullensis* (NCBI acc.: MF136530). Putative transcription initiation site (TIS) 5′-TATATTAGGGGGG-3′ and putative transcription termination site (TTS) 5′-CCCTCCCC-5′ as boundaries of the non-transcribed spacer (NTS) and external transcribed spacer (ETS) were identified based on the similarity those identified based on the comparative analysis of sequences with literature data concerning different plant species (Inácio et al., 2014; Hu et al., 2019). Dot-plot analysis of NTS and ETS region was conducted using LAST with threshold score = 39 (*E* = 8.4e-11) at online Mafft web interface^1^ (Katoh et al., 2019). Pairwise sequence identity of the NTS and ETS regions of intragenic spacer (IGS) were determined from the pairwise alignment of IGS regions with BioEdit (Hall, 1999). To determine the copy number of ribosomal DNA array in individual *Anemone* genomes, reads that were unmapped to the chloroplast genomes were mapped to the conserved 26S rDNA region of each ribosomal DNA array with bowtie2 v2.2.5 (Langmead and Salzberg, 2012), using the flags: –very-sensitive-local, –no-mixed, –no-unal, –no-discordant. Coverage information for the 26S rDNA was extracted using samtools coverage 1.11-2 (Li et al., 2009).

### Identification of Repetitive Sequences in the Intergenic Spacer

Forward, reverse, palindromic and complement repeats were identified using the online web interface for REPuter^2^ Kurtz et al., 2001) with the following settings: Hamming distance of 3 and minimum repeat size of 30 bp (Zhang et al., 2019). For the identification of tandem repeats, we utilized the online web interface for the program Tandem Repeats Finder^3^ Benson, 1999), the minimal repeat size was 10 bp and reported similarity percentage groups were 90-99 and 100% (Li et al., 2020).

### Phylogenetic Analysis

Phylogenetic relationships and inheritance patterns were reconstructed using the NTS and ETS regions of reconstructed ribosomal DNA arrays. Sequence alignment was conducted with Mafft v7.481 with selected E-INS-i method (Katoh et al., 2005). Maximum likelihood (ML) analysis of each dataset was conducted in IQ-TREE (Nguyen et al., 2015) with 10,000 bootstrap replicates and automatic selection of gene model TN + F + G4 for ETS tree and TPM3u + F + I + G4 for NTS tree. Phylogenetic trees were visualized in FigTree 1.4.4^4^.

### Bisulfite Conversion

Bisulfite conversion was done using the EZ DNA Methylation-Lightning Kit (Zymo Research, Irvine, California, United States) according to the manufacturer’s instructions. Approximately 300 ng of genomic DNA was used as input. The converted DNA was used immediately for PCR amplification or stored at −20°C for later use.

### CpG Islands Detection

To identify the presence of CpG islands within IGS of the 35S rDNA sequence, the NewCPGreport tool^5^ was employed. This program estimated putative “CpG islands” using the observed versus expected GC numbers as well as their grouping patterns in the sequence. As a default, the CpG island should contain >50% CpG and Obs/Exp ratio >0.6.

### Primer Design, PCR Amplification and Cloning of Bisulfite Converted DNA

Primers for gDNA of bisulfite converted DNA templates were designed with Primer3^6^ and MethPrimer 2.0^7^, respectively. Regions within the CpG1, CpG2, and CpG3 islands of *A. parviflora*, *A. sylvestris*, *A. cylindrica* (Supplementary Table 2 and Supplementary Figure 1) were selected. As a template for primer design, we used 35S rDNA sequence from *A. parviflora*, *A. sylvestris* and *A. cylindrica* obtained from RepeatExplorer. Specific primer pairs have been used for amplification of genomic DNA and bisulfite converted DNA. All PCRs were performed using GoTaq^®^ Green Master Mix (Promega, Madison, WI, United States): 1X GoTaq^®^ Green Master Mix, 10 pmol of each primer (Macrogen, Amsterdam, Netherlands) and 1 μl of template DNA (16 ng), in a 25 μl final reaction volume. The PCR program consisted of 35 cycles, each with 1 min denaturation at 95°C, 10 sec annealing from 54 to 58°C, 1 min extension at 72°C, and a final extension of 20 min. Annealing temperatures specific for each primer pair are presented in Supplementary Table 2. Amplicons were extracted and purified using ReliaPrep™ DNA Clean-Up and Concentration System and cloned into pGEM-T Easy vector according to the manufacturer’s instruction (Promega, Madison, WI, United States). Positive clones were PCR-screened using vector SP6 and T7 primers. Bisulfite-converted DNA (13 to 45 clones per sample) and genomic DNA (1-3 clones per sample) were Sanger sequenced by Macrogen (Amsterdam, Netherlands).

### DNA Methylation Analysis

The genomic and bisulfite converted sequences (clones) were overlapped by ClustalX sequence alignment tool 1.83 and aligned. To identify the methylated cytosines, the ClustalX alignment file was uploaded to CyMATE software platform at http://www.cymate.org (Hetzl et al., 2007; Müllner and Hetzl, 2008). The CG, CHG and CHH methylation contexts were analyzed. Statistical analyses were performed using Wilcoxon signed-rank test (function wilcox.exact, from exactRankTests (version 0.8-34) package) implemented in the R. *P*- values were corrected for multiple testing using the Benjamini-Hochberg adjustment, with α = 0.05.

## Results

### The Number of Active NORs Revealed by Silver Staining

**FIGURE 1:**
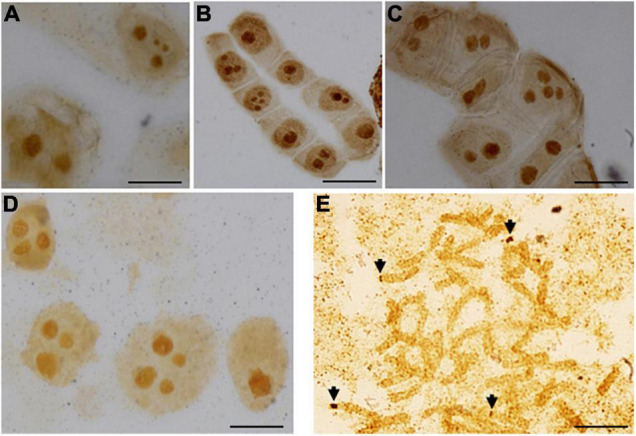
Silver stained nucleoli of **(A)** *A. cylindrica*, **(B)** *A. sylvestris*, **(C)** *A. multifida* and **(D)** *A. baldensis*. **(E)** Silver stained chromosomes of *A. baldensis*. NORs as well as NOR-bearing chromosomes are marked with arrow. Bar = 10 μm.

To determine the number of transcriptionally active 35S rDNA sites, we applied silver staining. The number of nucleoli in the individual cell and their frequencies were presented in Supplementary Table 1. The number of observed nucleoli ranged from one to four in diploids *A. cylindrica* and *A. sylvestris*, as well in allotetraploid *A. multifida* and allohexaploid *A. baldensis*. The most frequent number of nucleoli was two in *A. sylvestris*, *A. cylindrica* and *A. multifida* and three in *A. baldensis*. The maximum number of four nucleoli present in *A. sylvestris*, *A. cylindrica* and *A. multifida* corresponded to the four 35S rDNA sites, i.e., NORs, thus confirming that all rDNA sites can be active within the single cell (Figures 1A-C). In *A. baldensis*, the maximum number of four nucleoli, as well as the existence of four silver stained signals (e.g., NORs) in metaphase chromosomes indicate that in *A. baldensis* four out of eight 35S rDNA sites are active (Figures 1D,E).

Molecular organization of the 35S rDNA of allopolyploids *A. multifida* and *A. baldensis* of common origin and their progenitor species *A. cylindrica*, *A. sylvestris* and *A. parviflora*: a comparative analysis.

**FIGURE 2:**
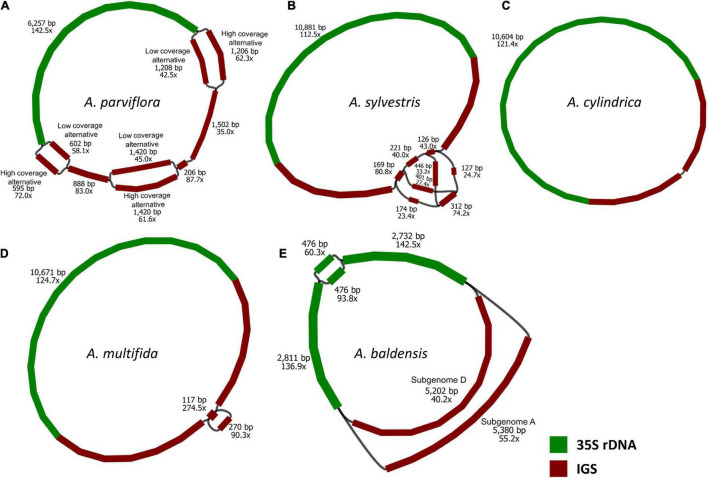
Graphical outputs from GetOrganelle showing assembled 35S rDNA array: **(A)** *A. cylindrica*, **(B)** *A. sylvestris*, **(C)** *A. parviflora*, **(D)** *A. multifida* and **(E)** *A. baldensis*. The colors represent IGS region (red) and 35S rRNA region (green).

The genome size results showed that the average 2C DNA value in pg was 30.05 ± 0.46 (credibility of value (CV%) = 1.51) for *A. multifida* and 42.01 ± 1.25 (CV% = 2.98) for *A. baldensis*. Genome size values for parental species are taken from the literature: *A. cylindrica* (2C = 18.6 and 21.4 pg, Rothfels et al., 1966 and Bai et al., 2012, respectively), *A. parviflora* (2C = 11.8 pg, Rothfels et al., 1966) and *A. sylvestris* (2C = 17.02 and 16.12 pg, Barow and Meister, 2003; Veselý et al., 2013, respectively). Using high-throughput genomic data, we assembled 35S rDNA units in all five *Anemone* species. The 35S rDNA assembly graphs which contained the complete 35S rDNA unit were constructed using GetOrganelle 1.7.3.4 (Jin et al., 2020; Figure 2). The total length of the 35S rDNA unit varied from 10,489 bp in *A. cylindrica*, 10,715 bp in *A. multifida*, 10,761-10,939 in *A. baldensis*, 11,269-11,278 bp in *A. parviflora* and 12,084 bp in *A. sylvestris* (Supplementary Table 3). The total length of the recovered 18S-5.8S-26S regions (including ITS1 and ITS2 sequences) varied from 5763 bp in *A. multifida*, 5764 bp in *A. sylvestris*, *A. parviflora* and *A. baldensis* to 5770 bp in *A. cylindrica* (Supplementary Table 3). The homogeneity of the 18S, 5.8S and 26S rDNA rDNA units was high with little variation in genic sequences and both ITS regions. The length variation was accounted for by differences in length of ITS1 of *A. sylvestris* and *A. baldensis* (176 bp), vs. *A. parviflora, A. multifida* and *A. cylindrica* (175 bp) which contained a single nucleotide deletion after nucleotide position site 55. The 18S, 5.8S and 26S rDNA sequences of *A. sylvestris*, *A. parviflora*, *A. cylindrica*, *A. multifida* and *A. baldensis* showed from 93.7 to 97% nucleotide identity with their counterparts from *Alnus jorullensis* subsp. *jorullensis* (NCBI acc.: MF136530).

The genome proportion that each 35S rDNA cluster comprised was calculated from the number of mapped reads to 26S rDNA sequences compared with the total number of reads used for mapping. Reads that mapped to the chloroplast genome were excluded from the analysis. From the genome proportion, the copy numbers of 35S rDNA sequences were estimated using the formula: coverage of 26S rDNA/genome coverage of reads used for mapping. Copy number per 1C ranged from 1381 in *A. cylindrica*, 1917 in *A. multifida*, 2147 in *A. sylvestris*, 2480 in *A. parviflora* to 5070 copies in *A. baldensis* distributed over two loci (Table 2).

**FIGURE 3:**
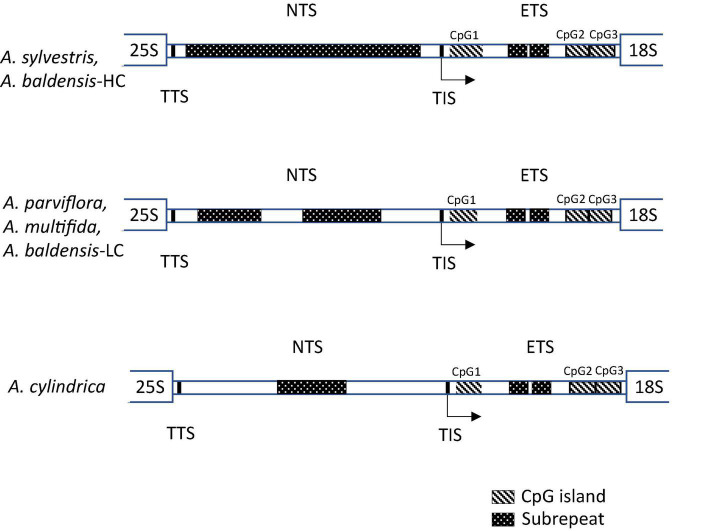
Structural organization of the IGS in *Anemone* and position of CpG1, CpG2 and CpG3 islands. IGS-intergenic spacer; ETS-external transcribed spacer; TTS-transcription termination site; NTS-non-transcribed spacer; TIS-transcription initiation site.

The size of IGS sequences generated by GetOrganelle varied from 4702 bp in *A. cylindrica*, 4935 bp in *A. multifida*, 4980-5158 in *A. baldensis*, 5487-5492 bp in *A. parviflora* and to 6302 bp in *A. sylvestris*. Sequence similarity of IGS ranged from 46.5 to 78.8%, being the lowest between *A. sylvestris* and *A. parviflora* (46.5%) and the highest between *A. cylindrica* and *A. baldensis* (78.8%). The elementary structure of the *Anemone* IGS sequence generated by GetOrganelle consisted of six distinct regions: NTS, ETS, TTS, TIS, SR, and CpG islands (Figure 3). With the help of the alignment of our sequence with the transcription initiation regions (TIS) of other species, including Poaceae (Chang et al., 2010; Huang et al., 2017; Hu et al., 2019), Cucurbitaceae (Zentgraf et al., 1990), Fabaceae (Kato et al., 1990), Brassicaceae (Gruendler et al., 1991), Solanaceae (Borisjuk et al., 1997), and Punicaceae (Parvaresh and Talebi, 2014), we found the putative TIS of *Anemone* may be TATATTAGGGG (positions 3500-3512 in *A. sylvestris*, 2402-2414 in *A. parviflora*, 2163-2175 in *A. multifida*, 1901-1913 in *A. cylindrica*, 2208-2220 in *A. baldensis*) where A after TATATT represent start position (+ 1) (Supplementary Table 3 and Supplementary Figure 2). We also found that in *Anemone* sp. the putative TTS is CCCTCCCC (Yang et al., 2015; Hu et al., 2019). The IGS of *Anemone* contains an abundant and highly variable number of methylation sites that are irregularly distributed along the whole sequence. Interestingly, the majority of CCGG motives are placed upstream 5′ end of the NTS. In total, we counted 16 (*A. sylvestris*, *A. baldensis*-HC variant), 17 (*A. cylindrica*), 36 (*A. multifida*, *A. baldensis*-LC variant), and 44 (*A. parviflora*-HC variant, *A. parviflora*-LC variant) CCGG motives. Furthermore, we counted from 169 (*A. cylindrica*) to 244 (*A. sylvestris*) CHG sites and from 632 (*A. cylindrica*) to 933 (*A. sylvestris*) CHH methylation sites in the IGS of five *Anemone* species investigated in this study. CHG and CHH sites are more frequent in the NTS than in the ETS region.

The region between the putative TTS and TIS represents the NTS (1899 bp in *A. cylindrica*, 2161 bp in *A. multifida*, 2206-2353 in *A. baldensis*, 2398-2400 bp in *A. parviflora*, 3498 bp in *A. sylvestris*), whereas the remaining IGS region is the ETS (2760 bp in *A. multifida*, 2760-2791 bp in *A. baldensis*, 2789 bp in *A. cylindrica*, 2790 bp in *A. sylvestris*, 3073-3080 bp in *A. parviflora*) (Supplementary Table 3). The NTS appeared as significantly more variable between investigated *Anemone* species in comparison to the ETS (Table 3). NTS showed 29.3-96.7% similarity among five *Anemone* species investigated in this study, being the lowest between *A. sylvestris* and *A. baldensis*-LC variant (29.3%) and the highest between *A. multifida* and *A. baldensis*-LC variant (96.7%), while the ETS showed 64-100% similarity, being the lowest between *A. cylindrica* and *A. baldensis*-LC variant (64%) and the highest between *A. sylvestris* 1, 2, and 3 variants (100%) (Table 3).

**TABLE 3:** Pairwise identity matrix for two Intragenic spaces regions (IGS), Non-transcribed spacer (NTS) above the diagonal (gray background) and external transcribed spacer (ETS) below the diagonal (blue background) of five Anemone species and their IGS variants identified in this study.

Pairwise identity (%)	*A. baldensis-HC*	*A. baldensis-LC*	*A. cylindrica*	*A. multifida*	*A. parviflora-HC*	*A. parviflora-LC*	*A. sylvestris 1*	*A. sylvestris 2*	*A. sylvestris 3*
*A. baldensis-HC*	-	32	50.8	32.4	32.2	32.5	67	67	67
*A. baldensis-LC*	64.6	-	46.4	96.7	54.9	56.2	29.3	29.3	29.3
*A. cylindrica*	92.5	64	-	46.5	44.9	45.7	48.2	48.2	48.2
*A. multifida*	64.7	99.7	64.1	-	53.2	54.4	29.6	29.6	29.6
*A. parviflora-HC*	79.6	78.8	78.5	78.9	-	94.9	30.4	32.7	32.7
*A. parviflora-LC*	79.4	78.7	78.4	78.8	98.03	-	30.6	32.3	32.3
*A. sylvestris 1*	99.71	64.8	92.6	64.9	79.8	79.6	-	91.2	90.8
*A. sylvestris 2*	99.71	64.8	92.6	64.9	79.8	79.6	100	-	93.5
*A. sylvestris 3*	99.71	64.8	92.6	64.9	79.8	79.6	100	100	-

**FIGURE 4:**
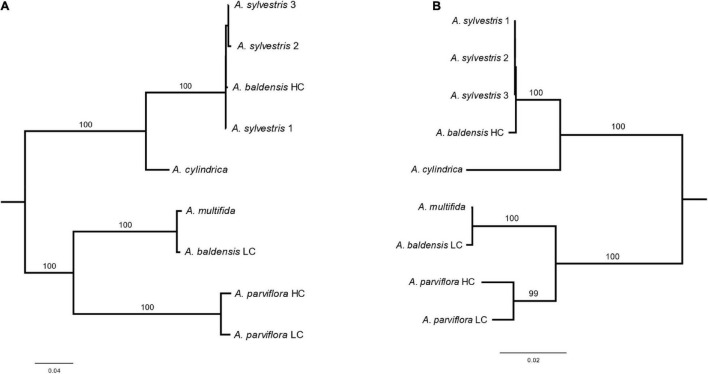
Maximum likelihood (ML) unrooted phylogenetic tree based on the alignments of the **(A)** Non-transcribed spacer (NTS) and **(B)** External transcribed spacer (ETS). Phylogenetic tree was produced in IQTree with 10,000 bootstrap replications. Values on the branches represent ML bootstrap values.

We identified two variants of IGS in *A. baldensis*: *A. baldensis-*HC (5159 bp) and *A. baldensis*-LC (4981 bp) (HC stands for High Copy and LC stands for Low Copy) (Figures 2, 3). The IGS variants differed in size, nucleotide sequence, abundance and origin. *Anemone baldensis*-HC and *A. baldensis*-LC variants exhibited 32% similarity in the NTS and 64.6% similarity in the ETS region. Furthermore, the *Anemone baldensis*-HC variant has higher coverage in comparison to the *A. baldensis*-LC variant along the whole IGS region (coverage proportion = 55.2x (*A. baldensis*-HC variant): 40.2x (*A. baldensis*-LC variant) (Figure 2E). The last, *Anemone baldensis*-HC variant is the most similar to the IGS of *A. sylvestris*, while the *A. baldensis*-LC variant is the most similar to the IGS of *A. multifida* (Figure 4 and Table 3). In *A. parviflora*, two IGS variants, *A. parviflora*-HC and *A. parviflora*-LC, have been observed, exhibiting high and low coverage, respectively. The variants have 94.9% of similarity in the region of NTS and 98.03% similarity in the region of ETS (Table 3). In *A. sylvestris*, three IGS variants, *A. sylvestris* 1, *A. sylvestris* 2 and A. *sylvestris* 3, have been observed. Interestingly, all three variants differ in the nucleotide sequence of the NTS, ranging from 90.8 to 93.5% of similarity, while the size is conserved (Table 3).

The analysis shows that among five *Anemone* sp. investigated in this study *A. sylvestris* possesses the highest number of subrepeats (SRs), both tandem and forward. In *A. sylvestris*, the NTS is highly enriched in SRs along the whole length (Figure 3 and Supplementary Figure 3). Similarly, the NTS of *A. baldensis* HC variant is also enriched with the SRs along its length, however, slight reduction in the number of SRs has been observed in the allopolyploid (Figure 3 and Supplementary Figure 3). The NTS of *A. baldensis*-LC variant, *A. multifida*, *A. parviflora* and *A. cylindrica* show significantly lower number of SRs in comparison to the NTS of *A. sylvestris* and *A. baldensis*-HC variant. *A. baldensis*-LC variant, *A. multifida* and *A. parviflora* exhibit similar distribution of SRs, located in two regions of the NTS. *A. cylindrica* possesses one region in the central part of the NTS enriched with the SRs (Figure 3 and Supplementary Figure 3). Contrary to the NTS, the ETS showed a more conserved number and organization of SRs among five *Anemone* species investigated in this study. In all five *Anemone* species SRs are located in two regions in the central part of the ETS (Figure 3 and Supplementary Figure 4). All five *Anemone* species investigated in this study showed similar number of repeats in the ETS region (Supplementary Figure 5). On contrary, all three *A. sylvestris* variants (1, 2, and 3) showed significantly higher number of repeats in the NTS region in comparison with the NTS region of *A. baldensis, A. cylindrica, A. multifida and A. parviflora* (Supplementary Figure 5).

The application of the CpG Island Report program was conducted using the following parameters: Obs/Exp ratio >0.60, C% + %G >50.00 and minimum length >200. Thus, the CpG-rich regions in the ETS identified in this study satisfied almost all of the criteria proposed for vertebrate CpG islands (Gardiner-Garden and Frommer, 1987): they were larger than 200 bases, had higher G + C contents than the surrounding DNA regions and had O/E CpG values close to or slightly higher than those statistically expected. Five CpG islands were observed in the ETS of *A. cylindric*a, *A. baldensis*-HC variant and *A. sylvestris*, while six CpG islands were observed in the ETS of *A. multifida*, *A. parviflora*-HC variant and *A. baldensis-*LC variant (Supplementary Table 4).

### DNA Methylation Analysis

**FIGURE 5:**
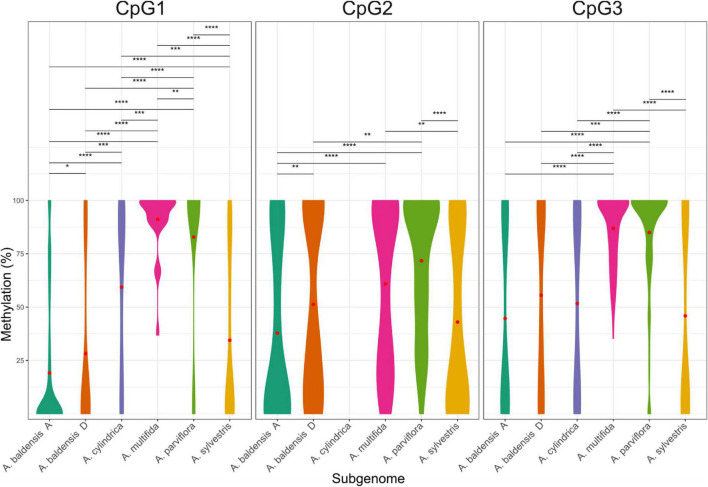
Violin plots representing the overall cytosine methylation level in regions CpG1, CpG2, and CpG3 within the 5′ETS of 35S rDNA established by bisulfite sequencing of Sanger-sequenced clones. Wider regions of the violin plot represent methylation levels that occur more frequently. The values on the graph represent percentage of the methylated clones per each site for each type of modification. Different colors represent different species. Red dots represent mean methylation level. Statistical significance is represented as: * for α ≤ 0.05, ** for α ≤ 0.01, *** for α ≤ 0.001 and **** α ≤ 0.0001.

To determine the level of cytosine methylation within the 35S rDNA, we used Sanger sequenced bisulfite-treated genomic DNA from three diploid and two polyploid *Anemone* taxa (Table 1). We were particularly focused on the CpG islands within the ETS as those regions appeared to be involved in transcription regulation (Deaton and Bird, 2011). In each species, we amplified three CpG islands, which corresponded to the CpG1′, CpG4′, and CpG5′ islands in *A. sylvestris, A. baldensis* HC and CpG1′, CpG5′, and CpG6′ islands in *A. cylindrica, A. parviflora-*HC, *A. multifida* and *A. baldensis*-LC, determined by Emboss Newcpgreport tool (Supplementary Table 4). Analyzed regions within the ETS are named as CpG1, CpG2, and CpG3 islands and are shown in Figure 3 and Supplementary Figure 1 (marked in bold). In *A. parviflora*, we sequenced and analyzed each of 14, 15, and 17 clones of CpG1, CpG2, and CpG3. In *A. cylindrica*, we sequenced and analyzed each of 13 clones of CpG1 and CpG3. In *A. sylvestris* we sequenced and analyzed 15 clones of CpG1, 15 clones of CpG2 and 16 clones of CpG3 (Table 4). In allotetraploid *A. multifida* we sequenced and analyzed 18 clones of CpG1, 15 clones of CpG2 and 37 clones of CpG3. All clones of *A. multifida* belonged to the D subgenome, since the 35S rDNA from the B subgenome is lost in the process of diploidization (Mlinarec et al., 2012a). In *A. baldensis*, we sequenced and analyzed 9 clones of CpG1, 11 clones of CpG2 and 34 clones of CpG3 of the A subgenome, and 22 clones of CpG1, 15 clones of CpG2 and 11 clones of CpG3 of the D subgenome (Table 4). Size of the analyzed sequences ranged from 211 to 637 bp (Table 4). The results of bisulfite sequencing are presented as diagrams at a single clone resolution (Supplementary Figure 6) and summarized in Table 4, Supplementary Table 5, and Figure 5. Table 4 contains average methylation frequencies, while Supplementary Table 5 contains methylation frequencies for each position within the CpG island.

**TABLE 4:** Methylation levels in the ETS of the 35S rDNA in studied species determined by bisulfite sequencing.

Species	Subgenome	CpG region	CpG1 variant	Size (bp)	Number of clones	methylated (%)	non-methylated (%)	CGN	CHG	CHH
*A. parviflora*		CpG1		230	14	95.43	4.57	92.31	97.67	96.14
		CpG2		346	15	71.67	25.95	98.82	93.29	60.29
		CpG3		379	17	87.40	12.60	99.31	94.12	86.43
*A. cylindrica*		CpG1		211	13	59.36	40.64	94.23	77.88	25.54
		CpG3		225	13	47.69	52.31	94.44	51.75	5.49
*A. sylvestris*		CpG1	short	277	2	28.68	71.32	65.00	35.00	5.80
		CpG1	long	637	13	33.25	66.75	72.27	44.14	10.97
		CpG2		280	15	41.55	58.45	93.65	42.00	12.63
		CpG3		267	16	42.69	57.31	97.48	47.60	4.18
*A. multifida*	D	CpG1	short	222	38	91.09	8.91	98.94	97.26	86.68
	D	CpG2		346	15	60.83	39.17	97.1	82	41.82
	D	CpG3		379	37	87.08	12.92	99.55	97.63	77.16
*A. baldensis*	A	CpG1	long	637	9	19.19	80.81	50.00	15.08	4.33
	A	CpG2		278	11	37.81	62.19	94.09	29.29	11.59
	A	CpG3		267	34	43.09	56.91	96.91	47.51	5.29
	D	CpG1	short	323	22	29.68	70.32	63.69	20.13	6.06
	D	CpG2		346	15	51.29	48.71	98.26	80.67	26.30
	D	CpG3		266	11	52.38	47.62	98.35	59.74	11.11

Total methylation frequency of CpG1 island ranged from 19.19 to 95.43%, being the lowest in the A subgenome of *A. baldensis*, and the highest in *A. parviflora*. Total methylation frequency of CpG2 island ranged from 37.81 to 71.61%, being the lowest in the A subgenome of *A. baldensis*, and the highest in *A. parviflora*. Total methylation frequency of CpG3 island ranged from 42.69 to 87.40%, being the lowest in the *A. sylvestris*, and the highest in *A. parviflora*. In general, CpG1 island showed lower methylation frequencies (19.19-59.36%), followed by the CpG2 island (37.81-60.83%), while CpG3 island showed the highest methylation frequency (42.69-87.40%). The only exception was the CpG1 island of *A. parviflora* and *A. multifida* showing considerably high methylation frequency, 95.43 and 85.21%, respectively (Table 4).

In *Anemone*, we found considerable variation between clones originating from the same individual (Table 4). For example, in *A. multifida*, three clones contained 54.17% of total methylation, while 25 clones exhibited 84.38-100% of methylation regarding CpG1 island.

In *Anemone*, the highest methylation was observed for ETS cytosine at symmetrical CG motifs (average methylation = ∼88%), followed by CHG motifs (average methylation ∼55.88%), while considerably low (average methylation of 23.31%) methylation was found at non-symmetrical (CHH) sites (Table 4).

### Discrimination Between the 35S rDNA Originating From the A and D Homoeologues in *Anemone baldensis*

In *Anemone*, two CpG1 size variants, CpG1-637 and CpG1-277, were revealed by cloning and sequencing of the CpG1 region, which were 637 bp and 277 bp long, respectively. Longer variant CpG1-637 dominated in *A. sylvestris* and its homologs in *A. baldensis* (the A subgenome), while shorter variant CpG1-277 dominated in *A. cylindrica, A. parviflora* and its homologs in *A. baldensis* (the D subgenome) and *A. multifida* (the D subgenome). Detailed analysis showed that variant CpG1-637 occurred from duplication of CpG1-277 (Supplementary Figure 7). Thus, in *A. baldensis*, shorter CpG1 variant (CpG1-277) originated from the *A. parviflora*-like subgenome, while longer CpG1 variant (CpG1-637) originated from the *A. sylvestris*-like subgenome. A primer pair designed to amplify the CpG1 region in *A. sylvestris* enabled discrimination of CpG1 regions from the A and D subgenomes of *A. baldensis* according to the length of the CpG1.

To study the comparative methylation of the CpG2 region between the A and D subgenome of *A. baldensis* we took advantage of the CpG2 divergence. Specific primers were designed to amplify the CpG2 regions of A and D homoeologous in *A. baldensis*. Primers synthesized according to the *A. parviflora* 35S rDNA sequence were used to amplify CpG2 region from the D subgenome, while primers synthesized according to the 35S rDNA of *A. sylvestris* were used to amplify CpG2 region from the A subgenome of *A. baldensis* (Supplementary Table 2).

The primers that were constructed according to the 35S rDNA sequence from *A. sylvestris* amplified CpG3 regions from both A and D subgenomes of *A. baldensis*, however, those were still discriminated according to the ten SNPs within the amplified sequence (Supplementary Table 2). The CpG3 island that was similar to those of *A. parviflora* originated from the D subgenome, while the CpG3 island similar to *A. sylvestris* originated from the A subgenome.

### Comparative 35S rDNA Methylation Analysis Between *Anemone parviflora* and *Anemone sylvestris* and Their Homologs in Allopolyploids *Anemone multifida* and *Anemone baldensis*

Results of this study clearly show a decrease in the methylation frequency (= hypomethylation) of the 35S rDNA between parental species and their allopolyploids, e.g., both allopolyploids showed a decrease in methylation level in the 35S rDNA loci. Hypomethylation was more prominent in allohexaploid *A. baldensis* than in allotetraploid *A. multifida*. In particular, average methylation frequency of the CpG1 and CpG2 regions in *A. parviflora* (95.43 and 71.67%, respectively) was higher in comparison to average methylation frequency in its homologs in *A. multifida* (91.09 and 60.83%, respectively) and *A. baldensis* (29.68 and 51.29%, respectively) (Table 4 and Figure 5). Average methylation frequency of CpG3 region was similar between *A. parviflora* (87.4%) and in its homologs in A. *multifida* (87.08%), and higher when compared with its homologs in *A. baldensis* (52.38%). Furthermore, average methylation frequency of CpG1 and CpG2 regions was higher in *A. sylvestris* (33.25 and 41.55%, respectively) in comparison to its homologs in *A. baldensis* (19.19% and 37.81%, respectively), while the average methylation frequency of CpG3 region of *A. sylvestris* (42.69%) was similar to its homologs in *A. baldensis* (43.09%) (Table 4 and Figure 5). Statistical analyses showed that the differences in methylation level of 35S rDNA between *A. parviflora* and its homologs in *A. baldensis* are statistically significant, while the differences between *A. parviflora* and its homologs in *A. multifida*, as well as differences between *A. sylvestris* and its homologs in *A. baldensis* are not significant (Supplementary Table 6).

**FIGURE 6:**
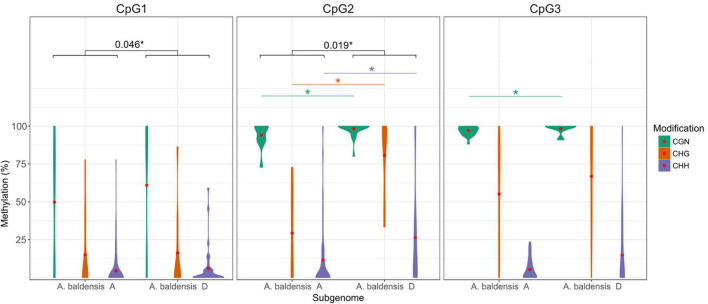
Comparative 35S rDNA methylation analysis between the subgenomes A and D of *A. baldensis*. Different colors represent different modifications. Red dots represent mean methylation level. Statistical significance for different modifications is represented as: * for α ≤ 0.05.

In allohexaploid *A. baldensis*, the D subgenome is more methylated than the A subgenome considering all three CpG islands, and also considering all three CGN, CHG and CHH classes (Table 4 and Figure 6). Statistical analyses showed that the differences in methylation level between the A and D subgenomes of *A. baldensis* are statistically significant considering CpG1 and CpG2 islands, while differences are not significant considering CpG3 island (Supplementary Table 7).

## Discussion

The sequence organization of the complete 35S rDNA array and the methylation status of three CpG-rich regions within the ETS was presented and compared for the first time in *A. multifida* (2*n* = 4*x* = 32, BBDD) and *A. baldensis* (2*n* = 6*x* = 48, AABBDD) and their diploid parental species *A. sylvestris* (2*n* = 2*x* = 16, AA), *A. cylindrica* (2*n* = 2*x* = 16, BB) and *A. parviflora* (2*n* = 2*x* = 16, DD).

### Polyploids *Anemone multifida* and *Anemone baldensis* and Their Parental Species *Anemone sylvestris, Anemone cylindrica* and *Anemone parviflora* Share the 35S rDNA Organization

The total length of the recovered 35S rDNA units varied from 10,489 bp in *A. cylindrica* to 12,084 bp in *A. sylvestris*. Comparative analysis of 35S rDNA between *Anemone* species showed that the size differences of the 35S rDNA was caused by the variability in sequence length of the IGS. In *Anemone*, the NTS (1899 -3498 bp) showed high variability in sequence length, in comparison to significantly less variable size of ETS (2760-3080 bp). Similar was observed in other plant genera such as *Stipa* sp. (*Poaceae*) in which the NTS was much more variable in sequence length (1453-2347 bp), than the ETS (677-684 bp) (Krawczyk et al., 2017).

Results of this study reveal great differences in the amount and position of SRs within the NTS between *Anemone*, while the ETS is less variable in the amount and position of the SRs. In *A. sylvestris* almost the complete NTS consists of SRs, while in *A. cylindrica* only a small central region of the NTS is composed of the SRs. Furthermore, in allohexaploid *A. baldensis*, reduction of SRs in *A. sylvestris*-derived 35S rDNA units has been observed. Thus, results of this study clearly show the fast-evolving nature of the NTS within the IGS and that the SRs are very likely responsible for generating such great variability. It is known that the SRs serve as recombination hotspots generating the high multiplicity of ribosomal genes (Rogers and Bendich, 1987). In *Fagaceae* and *Asteraceae*, variability in the length of IGS sequences was mainly due to different copy numbers of SRs in the NTS and ETS region, respectively (Linder et al., 2000; Inácio et al., 2014).

The IGS in *Anemone* (4702-6302 bp) fit well within the range of IGS of other plant species such as *Fagus sylvatica* (1715-1858 bp), *Quercus suber* (1980-2242 bp), *Stipa* sp. (2193-3098 bp), *Erianthus arundinaceus* (2955-3299 bp) and *Trillium* sp. (7400-12200 bp) (Yakura et al., 1983; Inácio et al., 2014; Krawczyk et al., 2017; Hu et al., 2019). Also, the ETS of *Anemone* (2760-3080 bp) fit well among known ETS lengths (680–3170 kb) (Linder et al., 2000; Vander Stappen et al., 2003; Inácio et al., 2014; Krawczyk et al., 2017; Hu et al., 2019). In *Helianthus*, the lengths of the ETS sequences varied from approximately 1600 bp (*H. annuus*, *H. argophyllus*, *H. praecox ssp. hirtus*) to 2100 bp (*H. atrorubens*) (Linder et al., 2000), while in *Stipa* sp. it ranged from 677 to 684 bp (Krawczyk et al., 2017).

Both regions, the NTS and the ETS, showed a strong phylogenetic signal (bootstrap values above 99 in both the NTS and the ETS trees) among five *Anemone* species investigated in this study (Figure 4). Furthermore, both the NTS and the ETS as molecular markers enabled to reveal the origin of allopolyploids *A. multifida* and *A. baldensis*. In agreement with the previous studies of Mlinarec et al. (2012a), *A. baldensis* high copy IGS variant (*A. baldensis*-HC variant) was the most similar to *A. sylvestris*, donor of the A subgenome, while *A. baldensis* low IGS copy variant (*A. baldensis*-LC variant) was the most similar to *A. multifida*, as well as to *A. parviflora*, donor of the D subgenome. Thus, the results of this study confirm that *A. sylvestris* (AA) and *A. multifida* (DD) are ancestral species of *A. baldensis* (AABBDD). Nonetheless, owning to its higher number of sub-repeats and due to the overall variability of the NTS region, the NTS region might not be a suitable marker for the phylogenetic inference in other distantly related species. Furthermore, in *A. baldensis*, *A. sylvestris*-derived 35S rDNA units are presented in considerably higher copy number than the *A. parviflora*-derived 35S rDNA units (Table 2). This result is in line with previous results of Southern blot and FISH experiments showing the presence of significantly weaker 35S rDNA signals in the *A. parviflora*-like bands (Southern blot) and chromosomes (FISH) in comparison to the *A. sylvestris*-like ones (Mlinarec et al., 2012a). Baldwin and Markos (1998) calculated an approximately 1.3- to 2.4-fold higher rate of sequence evolution by nucleotide substitution in the ETS region studied than in ITS1 + ITS2 in *Calycadenia* (Compositae). Due to the extremely high rate of evolution within the IGS region, it managed to resolve the complex intraspecies relationships in Asteraceae, Poaceae and Compositae (Baldwin and Markos, 1998; Linder et al., 2000; Krawczyk et al., 2017).

In *Anemone*, the ETS is discretely GC-rich (52.38-53.64%). The CpG islands found in the ETS account for the GC richness of this region. The placement of CpG islands within the ETS in *Anemone* is similar to cork oak and beech which exhibited 54 and 62% GC, respectively (Inácio et al., 2014). In *Arabidopsis thaliana* CpG islands were found in the SR region that is discretely rich in GC (53%; Gruendler et al., 1991).

While the length of the IGS varied in size between the *Anemone* species, its units present a specific order of conserved motifs in closely related species. *Anemone*, investigated in this study showed an organization typical of most ribosome IGS composed of NTS, ETS, TTS, and TIS, with structural features of plant IGS sequences and all functional elements needed for rRNA gene activity (Hu et al., 2019). Potential transcriptional enhancers and promoters for the RNA polymerase I machinery and one putative transcription initiation site were detected in all five species based on comparisons with the TIS motif of other species (Kato et al., 1990; Zentgraf et al., 1990; Gruendler et al., 1991; Borisjuk et al., 1997; Chang et al., 2010; Parvaresh and Talebi, 2014; Huang et al., 2017; Hu et al., 2019). TIS region detected in investigated *Anemone* has a TATATT sequence upstream the initiating A, like in the majority of the plants studied so far, including the Gymnosperms (Linder et al., 2000; Hu et al., 2019). Moreover, in *Anemone* the TIS sequence, TATATTAGGGG, is the most similar to those of *Saccharum officinarum* and *Saccharum robustum* from the Poaceae family (Hu et al., 2019). The pyrimidine-rich motif CCCTCCCC, serving as proximal terminator at the 5′end of 26S rDNA, is also highly similar to the TTS at the 5′end of IGS in other plants (Yang et al., 2015; Hu et al., 2019).

Cloning and sequencing of the CpG1 region in *A. sylvestris* revealed two size variants, CpG1-637 and CpG1-277, downstream of the TIS region. The longer variant originated as duplication from shorter variant and it probably arose during divergence of *A. sylvestris*, as it is absent in *A. parviflora* and *A. cylindrica.* The presence of two size variants allowed discrimination of CpG1 islands according to the parental origin in *A. baldensis*. Such intraspecies length variability in the IGS of 35S rRNA genes is shown in other plant species, e.g., *Atropa*, *Aegilops*, *Nicotiana*, *Prunu*s and *Brachypodium* (Volkov et al., 1993, 2017; Borisjuk et al., 1997; Shcherban et al., 2008; Borowska-Zuchowska et al., 2020).

### The Methylation of the 35S rDNA in *Anemone*

In seed plants and most animals, the multicopy genes encoding ribosomal RNA (rDNA) typically exhibit heterochromatic features and high levels of DNA methylation (Matyášek et al., 2019). *Anemone* species analyzed in this study show wide range of methylation in 35S rDNA loci, determined by bisulfite sequencing, from relatively low levels (19.19%) to high levels (95.43%) in the CpG1 islands of *A. baldensis* and *A. parviflora*, respectively. Methylation frequencies observed in *Anemone* in this study fit well within the methylation frequencies observed in other seed plants. *Brassica rapa* and *B. oleracea* showed 30 and 17%, respectively, of the overall methylation level of ETS revealed by bisulfite sequencing (Shcherban et al., 2008). *Fagus sylvatica*, *Quercus faginea*, *Q. rubra* and *Castanea mollissima s*howed high levels of methylation (≥80%), while in *Q. suber*, *Q. pyrenaica* and *C. sativa* the levels of methylation were lower, ranging from 30 up to 60% regarding the *Bam*HI restriction site within 26S rDNA (Inácio et al., 2014). Concerning the 18S rRNA gene, only *Q. suber*, and *Q. faginea* showed moderate levels of methylation: 30 and 15%, respectively, while *F. sylvatica*, *Q. rubra*, *C. sativa* and *C. mollissima* showed low level (<3%) of methylation regarding *Bam*HI restriction site (Inácio et al., 2014). Furthermore, *Cycas revoluta* showed moderate levels of methylation (approximately 33% of total methylation) in 35S rDNA as revealed by bisulfite sequencing of 23 Sanger-sequenced clones (Wang et al., 2016). *Solanum lycopersicum* and *Theobroma cacao* showed moderate levels of 35S rDNA methylation, 33 and 24.2% of total methylation, respectively, determined from high-throughput bisulfite sequencing (Matyášek et al., 2019). Contrary to plants, bryophyte showed little or no methylation in both 35S and 5S loci, determined from high-throughput bisulfite sequencing (Matyášek et al., 2019). Total methylation levels of 35S rDNA, including both ITSs and non-repetitive parts of IGS subregions, ranged from negligible (1.7% in *Physcomitrella patens*, 2.4% in *Polytrichum formosum*, 6.9% in *Marchantia polymorpha*) to moderate (16.6% in *Dicranum scoparium*) (Matyášek et al., 2019). In bryophytes, low levels of CG and CHG methylation in 35S rDNA have been confirmed by two assays, using methylation-sensitive restriction enzymes and by High-Resolution Cytosine Methylation Analysis of rDNA by Whole Genome Bisulfite Sequencing (Matyášek et al., 2019).

Considerably high variations in 35S rDNA methylation frequency between species studied so far could be an artifact caused by different methods used and also by the fact that in most studies only a relatively small number of copies within an array was analyzed. Comparing data from different studies that used different methods can lead to misinterpretation of the results. Therefore, in addition to focusing on epigenomics of single cell type and increasing data sets, methylation data should be confirmed by more than one assay, which would provide a more balanced, comprehensive and critical view of the research issue (Kumar et al., 2018).

Of special interest within the 35S rDNA array is the IGS of rRNA genes, where the promoter region (TIS) and other regulatory elements are located. CpG islands within IGS with higher average GC content are associated with regulation of rRNA gene expression regarding the cytosine methylation (Berger, 2007). Furthermore, the methylation property of the CpG islands within the ETS may have major implications on 35S rRNA gene regulation. In *Anemone*, segments of different methylation frequencies may have derived from arrays that have different impact on the transcription regulation of 35S rRNA genes. The proximity of CpG1 island to the TIS region and its lower level of methylation in comparison to distantly located CpG2 and CpG3 island, revealed in this study, suggests its putative role in regulation of gene transcription mediated by DNA methylation. It is estimated that about 60% of promoter sequences are located close to CpG islands in different organisms and, contrary to expectation, these are not highly methylated areas even though they contain a relatively high GC amount (Antequera, 2003; Deaton and Bird, 2011). In bryophytes *Polytrichum formosum, Physcomitrella patens*, *Marchantia polymorpha*, *Dicranum scoparium*, as well as angiosperms *Solanum lycopersicum* and *Theobroma cacao*, the methylcytosine density was relatively uniform across the rDNA units with no apparent differences between coding and non-coding regions, established by whole genome bisulfite sequencing (Matyášek et al., 2019).

Results of this study showed that *A. parviflora* and *A. multifida* exhibit significantly higher methylation levels of 35S rDNA (95.43 and 91.09%, respectively) in comparison with *A. cylindrica, A. sylvestris* and *A. baldensis* (19.19-56.36%) in the same region of ETS (CpG1 island). One possible explanation is that differences in methylation level could be related to the age of the leaves and/or individual. However, we consider this hypothesis less likely as gDNAs of all plants used in this study were isolated from the young leaves and from individuals of similar age. Recent investigations of Potabattula et al. (2020) consistently showed that methylation of the rDNA transcription unit including upstream control element (UCE), core promoter, 18S rDNA, and 28S rDNA in human sperm significantly increased with donor’s age, revealed by using bisulfite pyrosequencing.

In *Anemone* all 35S rDNA loci are located distantly at the ends of chromosomes (Mlinarec et al., 2012a,b). Therefore, it is not likely that methylation in *Anemone* is related to the location on chromosomes. In *Arabidopsis*, 5S rDNA units located proximally to centromeres were more methylated than those located distantly (Vaillant et al., 2008).

In *Anemone* we observed considerable variations in methylation of CpG1 island between clones originating from the same individual. Astonishing epigenetic variation in methylation patterns was observed in other plants such as *Brassica napus* in which virtually none of the clones had identical methylation profile (Książczyk et al., 2011). In *B. oleracea* the number of methylated Cs ranged between 6–35%, while it ranged between 19–40% in *B. rapa* (Książczyk et al., 2011). Variations could be due to the differences in transcriptional status of the rRNA genes within the array. It is considered that within the tandemly arranged units, highly methylated genes are heterochromatic and inactive, while genes with low or no methylation levels are active (Suzuki and Bird, 2008). In *A. multifida*, moderately methylated CpG1 clones with 54.17% of methylation possibly originate from the active part of 35S rDNA arrays, while completely methylated CpG1 clones (100% of methylation) probably originate from inactive part of 35S rDNA arrays. In mammals, only about one hundred of 45S rRNA genes are estimated to be transcribed at any one time (Grummt and Pikaard, 2003). In *Danio rerio* (zebrafish) only twelve 5S rDNA copies appeared to be active in oocytes, while a large number of genes in another locus are silenced and activated only at later developmental stages (Locati et al., 2016). Alternatively, in *A. multifida*, completely methylated 35S rDNA fraction may derive from rDNA pseudogenes. So far, we have no evidence that 35S rDNA pseudogenes constitute a significant proportion of 35S rDNA arrays in *Anemone*. Previous work showed that *A. apennina* had significant deletions in both spacer and 5S rDNA coding region, however, pseudogenic character of 5S rDNA remained to be confirmed (Mlinarec et al., 2016). On the contrary, in *Cycas revoluta* pseudogenes showed less methylation (25%) compared with functional genes (53%), which is explained by a reduced number of symmetrical methylated sites in pseudogenized copies (Wang et al., 2016).

In all investigated *Anemone* species in this study, the CG and CHG sites appeared consistently to be more frequently methylated than the non-symmetrical CHH sites. Higher methylation frequency of the CG and CHG sites in comparison to the non-symmetrical CHH sites is typical for plant rDNA (Garcia et al., 2012). In *Cycas revoluta*, 35S rDNA cytosines at symmetrical CG and CHG motifs were shown to be highly methylated (>50% of cytosines), while low (c. 7%) methylation was found at non-symmetrical (CHH) sites (Wang et al., 2016). In *Artemisia*, *Tagetes*, *Helichrysum*, *Elachanthemum* and *Helianthus* the methylation density at different motifs had the identical tendency descending in order: CG > CHG > CHH (Garcia et al., 2012). In *Solanum lycopersicum* and *Theobroma cacao*, 35S rDNA cytosines at CG motifs exhibited 60.1 and 73.5% methylation, respectively, while the methylation at CHH sites was 15.6 and 4%, respectively (Matyášek et al., 2019).

### Variation in rDNA Methylation Between Allopolyploids *Anemone multifida* and *Anemone baldensis* and Their Parental Species *Anemone sylvestris* and *Anemone parviflora*

Uniparental elimination of rDNA is a regular process of DNA deletion induced by allopolyploidy (Kovarik et al., 2008; Shcherban et al., 2008). In our previous work we showed that allopolyploids *A. multifida* and *A. baldensis* were subdued by the complete elimination of the *A. cylindrica*-like 35S rDNA units (Mlinarec et al., 2012a). There are many examples of other allopolyploid species undergoing uniparental inheritance such as *Aegilops*, *Brachypodium*, *Brassica* and *Nicotiana* (Kovarik et al., 2008; Shcherban et al., 2008; Książczyk et al., 2011; Borowska-Zuchowska and Hasterok, 2017). Results of this study showed a copy number reduction of the *A. parviflora*-derived 35S rDNA units in *A. multifida* and *A. baldensis*: from 2480 copies per haploid genome in *A. parviflora* to 1916 and 2136 copies in the *A. parviflora*-derived subgenome of *A. multifida* and *A. baldensis* (the D subgenome), respectively (Table 2). Contrary, a marked amplification of *A. sylvestris*-derived 35S rDNA units has been observed in *A. baldensis*: from 2147 copies per haploid genome in *A. sylvestris* to 2933 copies in the *A. sylvestris*-derived subgenome of *A. baldensis* (the A subgenome) (Table 2). Contraction of copy number of some rDNA families inherited from one parent was observed in *Aegilops sharonensis x Ae. umbellulata* (Shcherban et al., 2008).

The expression of 35S rRNA genes in individual 35S rDNA loci can be assessed by silver staining. We demonstrated in this study that in *A. multifida* all two *A. parviflora*-derived 35S rDNA loci are active. On the contrary, presence of the highest number of four nucleoli in *A. baldensis* suggest that in this species two 35S rDNA loci may be suppressed. Since nucleolar fusion frequently occurs during the cell cycle (mononucleation), only the highest number of nucleoli estimated in a large sample size should be taken as evidence of the number of active rDNA loci (Ochatt and Seguí-Simarro, 2021; Rosselló et al., 2022). In this study the number of nucleoli was estimated in relatively large samples size of 536 cells in four individuals (Supplementary Table 1). This is further confirmed by performing silver staining on the metaphase chromosomes of *A. baldensis* revealing the presence of four silver stained signals, e.g., four NORs located on two pairs of acrocentric chromosomes (Figure 1E). Higher methylation frequency in the *A. parviflora*-derived 35S rDNA units in comparison to the *A. sylvestris-*derived 35S rDNA units might indicate suppression of *A. parviflora*-derived NORs in allohexaploid *A. baldensis*. In allotetraploid *Aegilops sharonensis x Ae. umbellulata* differences in the expression level between individual NOR loci were not strictly correlated with gene dosage in a particular locus (Shcherban et al., 2008).

To test the 35S rDNA methylation changes in response to allopolyploidy, we performed bisulfite sequencing of CpG islands within the ETS. Considerably higher level of methylation in the 35S rDNA units of both *A. sylvestris* and *A. parviflora* in comparison to its homologs in *A. baldensis* (the A and D subgenomes) suggests that the repressed 35S rDNA copies may be reactivated in a polyploid. Thus, it seems that the *A. parviflora*-derived 35S rDNA loss and *A. sylvestris*-derived 35S rDNA expansion were accompanied by hypomethylation of the ETS within both the *A. parviflora*-derived and *A. sylvestris*-derived 35S rDNA units. Hypomethylation of the promoter region within rDNA units from one subgenome was observed in synthetic allotetraploid *Aegilops sharonensis x umbellulata*, revealed by digesting DNA with isoschizomers *Hpa*II and *Msp*I, which have different sensitivities to cytosine methylation (Shcherban et al., 2008). Contrary to *A. baldensis* and *Aegilops sharonensis x umbellulata*, in allopolyploid *Brassica napus*, C-genome NORs became hypermethylated, while the methylation status of A-genome NORs remained unchanged (Książczyk et al., 2011). Further, in the hybrid grass *Brachypodium hybridum*, the methylation level of *B. distachyon* homologs remained unchanged, while those of *B. stacei* became hypermethylated (Borowska-Zuchowska et al., 2020). In bryophytes, the rDNAs were unmethylated both in gametophyte and sporophyte tissues suggesting that, within species, the ploidy level and developmental stage do not influence methylation (its absence) at this locus (Matyášek et al., 2019).

Interestingly, we showed that the hypomethylation of *A. parviflora*-derived 35S rDNA was more prominent in allohexaploid *A. baldensis* than in allotetraploid *A. multifida* which could be related with the hybrid vigor. Namely, *A. baldensis* shows greater hybrid vigor in comparison to *A. multifida*. It is shown than in *Arabidopsis*, decrease in DNA methylation 1 is required to produce a full level of hybrid vigor (Kawanabe et al., 2016).

Considerably higher levels of DNA methylation of the *A. parviflora*-like 35S rDNA units in comparison to the *A. sylvestris*-like 35S rDNA units considering CpG1 and CpG2 islands within ETS, indicate the involvement of DNA methylation in establishing and maintaining the Nucleolar Dominance (ND) in allohexaploid *A. baldensis*. The preferential methylation of the *A. parviflora*-like 35S rDNA loci accompanied by the copy number decrease indicates that in *A. baldensis* the ND is toward the *A. sylvestris*-like 35S rDNA loci. Considerable number of studies on plants that exhibit ND demonstrate significant differences in the DNA methylation levels between the transcriptionally active and repressed rDNA units (Vieira et al., 1990; Neves et al., 1995; Chen and Pikaard, 1997; Houchins et al., 1997; Komarova et al., 2004; Lawrence et al., 2004; Costa-Nunes et al., 2010; Dobešová et al., 2015; Borowska-Zuchowska et al., 2020). In hybrid grass *Brachypodium hybridum*, the actively transcribed D-genome 35S rDNA units exhibited a low DNA methylation level, while the *B. stacei*-like rRNA genes were characterized by a high level of DNA methylation (Borowska-Zuchowska and Hasterok, 2017).

In *Anemone* polyploids, the ND was observed in leaves, however, other tissues might show different pattern. A tissue-specific expression pattern of the rDNA homeologs has been observed in many plant hybrids and allopolyploids (Volkov et al., 2007; Borowska-Zuchowska et al., 2021). For instance, the ND was stable in leaves but not in roots of *B. hybridum* (Borowska-Zuchowska et al., 2021). Furthermore, although a fully established ND was observed in leaves, a trace expression of the *A. thaliana*-derived rRNA genes was detected in the root-tip cells of the mature *Arabidopsis suecica* plants (Pontes et al., 2007).

Our preliminary plastid phylogenomics of *Anemone* reveal that in polyploid *A. baldensis, A. sylvestris*-derived subgenome is maternal indicating that in *A. baldensis* the ND is toward the maternal (sub)genome. Maternal control of 35S rDNA expression and epigenetic silencing of paternal 35S rRNA was observed in *Xenopus* hybrids (Michalak et al., 2015). However, there are also examples in the literature that the ND is independent of maternal or paternal effects (Sleutels et al., 2000); or that the ND in plant allopolyploids is not a maternal effect (Chen and Pikaard, 1997).

## Conclusion

In this study, we provide in depth comparative analysis of the 35S rDNA methylation among five *Anemone* species. The organization of 35S rDNA is presented for the first time in any member of the Ranunculaceae family so far. A heterogenous methylation pattern across the ETS is shown. Our research uncovers contrasting changes in 35S rDNA copy number in allopolyploid *Anemone baldensis* accompanied with diverse methylation frequencies within 35S rDNA arrays between different subgenomes within allopolyploid. These observations suggest that in *A. baldensis* the nucleolar dominance (ND) is toward *A. sylvestris*-derived homologs resulting in considerable disruption at both genomic and epigenomic levels. Taken together, this work enhances our current knowledge of the 35S rDNA organization in *Anemone* and provides evidence for progenitor-specific 35S rDNA methylation in the ND.

## Data Availability Statement

The data presented in the study are deposited in the repository SRA-NCBI under the BioProject ID PRJNA830872(http://www.ncbi.nlm.nih.gov/bioproject/830872), accession numbers SAMN27739250, SAMN27739251, SAMN27739252, SAMN27739253, SAMN27739254.

## Author Contributions

VB, JM, and NM conceived the study and designed the experimental part of the study. TB carried out by the field work. JM, NM, and AJ carried out the DNA extraction, bisulfite sequencing and cloning. VB carried out the silver staining. LB performed the bioinformatic and statistical analyses. JM and NM designed the primers. SY performed the flow cytometry. JM carried out the DNA methylation analysis, wrote the manuscript, and interpreted the results. All authors read, edited, enhanced the original version of the manuscript, and approved its final version.

## Conflict of Interest

JM was employed by Oikon ltd.-Institute of Applied Ecology. The remaining authors declare that the research was conducted in the absence of any commercial or financial relationships that could be construed as a potential conflict of interest.

## Publisher’s Note

All claims expressed in this article are solely those of the authors and do not necessarily represent those of their affiliated organizations, or those of the publisher, the editors and the reviewers. Any product that may be evaluated in this article, or claim that may be made by its manufacturer, is not guaranteed or endorsed by the publisher.

## Footnotes

1
https://mafft.cbrc.jp/alignment/server/

2
https://bibiserv.cebitec.uni-bielefeld.de/reputer/

3
https://tandem.bu.edu/trf/trf.html

4
https://github.com/rambaut/figtree/

5
https://www.ebi.ac.uk/Tools/seqstats/emboss_newcpgreport/

6
https://bioinfo.ut.ee/primer3-0.4.0/

7
www.urogene.org/methprimer2/tester-invitation.html

## Abbreviations

5-mC: 5-Methylcytosine
bal: *Anemone baldensis*
cyl: *A. cylindrica*
DAPI: 4′,6′ -diamidino-2-phenylindole
ETS: external transcribed spacer
FISH: fluorescence *in situ* hybridization
IGS: intergenic spacer
ITS: internal transcribed spacer
mul: *A. multifida*
ND: Nucleolar dominance
NOR: Nucleolar organizer region
NTS: non-transcribed region
par: *A. parviflora*
rDNA: ribosomal DNA
SR: subrepeat
syl: *A. sylvestris*
TIS: transcription initiation site
TTS: transcription termination sites.
