# Supplementary material for: Structure and Methylation of 35S rDNA in Allopolyploids Anemone multifida (2n = 4x = 32, BBDD) and Anemone baldensis (2n = 6x = 48, AABBDD) and Their Parental Species Show Evidence of Nucleolar Dominance
Source: Front Plant Sci. 2022 Jul 6;13:908218. doi: 10.3389/fpls.2022.908218 (PMC9296772; doi:10.3389/fpls.2022.908218)
Supplement: Supplementary file 1 [file Image_1.pdf]

Supplementary Figure S1. ETS sequence of *Anemone* sylvestris (Asyl_ETS), *A. parviflora* (Apar_ETS), *A. cylindrica* (Acyl_ETS) in fasta. The primers used for amplification of CpG islands in bisulfite sequencing assay for all five *Anemone* species are in yellow, the sequenced regions are in bold.

>Asyl_ETS

GGAGGTGTTGGTTGGATCCCTGGGAAAAAAAAAATTTCCCCCCGAGTTGGTGAAAGGCCAGCAATGCCCACACG
ATGCATCGACCAATGCATGTGAGCGTGTGGGCATGTCCCGATGAGGTCCCTTTTGAGTTTCC**AACACCGAACAAA**
CCACGTGGGGGCGTGGGAATGGCTCTCGATCACCCTATTGGTACGGGCGATCCTGGGCGACGGCTGGGCAATG
GCTGGGCGTTTGCTGGGCATTCGCTGGGCGTTCTCTGGGCGATACATGGGCCGGTGATTCGGCGTATCACCATG
AGATAATTCCTTGCCATCGTCCATGGCGCGTCACGAGATGGTGTTGGCTGGATGGTTGGGCACATGGGCATAGC
TTGGGAGATCCCCGGGCAATATGTGGGCATTGGATGGTTGGCGGATATCTTGTGAATGTTTTGAGATCAGCACGT
GGAGCGTTGCTCGGGCTGGTGCTGAGCAACCCCCAAATATGTGAGCTGGGTACTTTTTGTGGACGGTTGTCAATC
CTCATGCTTGGTAGCTATGAAATGAAATGTTCTCCATGCCTCTCCCTCGTATAACTTGCCCCGATACTATCAGGCGA
CTGTGTTTGTCTCCCATGCAGCATCTTAGTCCGTATATCGCTCAATCCGGTTACATTCGTTTGTAACGCTGCGGCGC
TGGTGGCCAAAGCGGGCGATGGAATAAGGTGTTGCATGGGGGAAATCATGGGCTGTTACTTTGATGCGGGGGGA
ACATTCAGGATATTGCTACTAGATTGCGTGTTGGGAAGACTACCGTGCCCAATAACCCAGTTTGCACCCTTCCGACT
CCACGTAGTGCAAGCACAAAATGTCCACGAGATGCCTGATACTGTTCCGAAGCTTTTC**ATGTTGGTTTGAAACTTT**
TGCCCCGATAGTGTGTCCTGACCGTTGCAGCAAGATTTTGCGCACAGCGAGCGTTTCGAATGGCGCCTCGTTCTA
CCATATGTATTGCCCTTTGCGGGGTGATGCACACATGGGTGAGCGGGCATTGTTCCCAACGCAACGTGTTGGCG
TGTGAGTGGTAGTAGAGACATCTCTGCTTTTGGGCTCCGTGCTTCGCGCATCGAACCATAAGCACATGTTTCCCT
CATTGGCGTCCGGCGTACTTGACTGTATGCCGGCAAGTAAAATGGTTCCTGTGTTGCCTACTCGGAGAAATTGGA
AAAT**ATCATTGCTAACGAGATTCATTGCCTTTGGTCGGCCAAAAGCTGGCCGAGGGCAACATGTTAGTGTGGCTT**
GTGAAACCTACCGTGGCATTCGCCCGCGTGTCTGGCTAGCAAGCTCGCACGTGCTTGGGGATATGGAAAACATA
GATGGGTCAGGGTTTCAAACTCGGCCGATGCAATTGTAGCTAATTTTGTGACCGCCGGACCGTAATGATAAGTC
CTACTCCCTGCGTGACCAGGTGTAGTTAGGCGCGGCGCGGACAACCGGCGACATAAAGGAATGCGTCTCAAAG
ATTAAGCCATGCATG

>Aparv_ETS

GGAGGTGTTGGTTGGCTCCCAAAAAAAAAAAATTTCCCCCTCGAGCTGGTGAAAGGCCAACAATGCC**CACACGTC**
TCTCATGCATCCCCCAATGCATGTGAGCGTGTGGGCATGTCCCGATGAGGACCCTTCGAGTTCACAACACCACGT
GGGGGCGTGGGAATGGCTCTCGATCACCCTATTGGCACGGGCGACGGCTGGGCGATGGCTGAGCGATGTTGGG
CGCTCTTTGGGCGATACATGGGCCAGTGATTCGGCGTATCGCCATAAGAGAATTCCTTGCCATCGTCCATGGCAC
GTCACGAGATGGTGTTGGAAATAGTTGGCTGGTGGAAAAAAAAACCCATTGGATGGACCGTCGTGAGAACGG
GTTGGTTGGATGGTTGGGCGCATGGGCGCAGCTTGGGAGATCCCCGGGCAATCGATGGGCATTGGATGGTTGG
CGGACATCTTGTGAACGTTTGTGATCACCATGTGGAGCATTGCTCGGGCAGGTGCTGAGCAACCCCAAATATGT
GAGCTGGGTACTTTTTGTGGACGGTTGTCAATCCTCATGCTTGGTAGCTATGAAATGAAGGGGGTTCACCACGCCT
CTCCCTCGGATAACCTTCCGGGCCCATACCACACGTTCATTTGACCTGTTTAAGCCGCTTCATGTGCGAAAAAAAAC
ATGGTCTTCTCAACGAGTGGCTTAGGCTTGGGGGTGTGAGGGGGGAGGAGTGTGGTGCTGCCCACGATGCATCA
ACTTGCCCCGATACTATTAGACGATCTTGTTCGTCTCCCATGCAACATCTTAGTCCGTATATCGCTCAATCCGGTTAC
ATTCGTTTGTAACGCTGCGGCGCTGGTGGCCAAAGCGGGCGATGGAATAAGGTGTTGCATTGGGGAACATCATG
GGCTGTTACTTTGATGCTGGGACCTTCAGGGCATTGCTACTAGATTGCGTGTTGGGGAGACTCCCGTGCCCAATAA

CCA**GTTTGCACCCATCCGGCTCCACGTAGTGCAATGACAAAATGTTCAGGAGATGCCCCGATACTGTTCTGAAGC**
TTTTCATGTTGGTTCGAAACTTGCCCCGACAGTGTGTCCCGACCGTTACAGCCAGATTTCGCGCACAGCGAGTGT
TTCGAACGGTGCTTCGTTCTACCATACGTATTGCCCTTTGCGGGGTAATGCACACATATGGGTGAGCAGGCATTG
TTCCCAACGCAACGTGTTGGCGTGTGAGTGGTAGTAGAGACATCCCTGCTTTTGGGCTCCGTGCTTCGCGCATCG
AACCATAAGCACATGTTTCCCTCATTGGCGTCCGGCGTACTTGATTGTATGTCGGCAAGTAAAATGGTTCCTGTG
CTCCCTACCCGAAGAAAGTGGAAAAGATCATTGCTAACGAGATTTATTGCCTTTGGTCGGCCAAAAGCTGGCCG
AGGGCAACATGTTAGTGTGGCTTGTGAAACCAACCGCGGCGTTTGCCCGCGTGTCTGGCTAGCGAGCTTGCACG
TGCTTGGGGATATGGAAAACATAGATGGGTCAGGGTTCTGAACTCGACCGACGCAATCGTAGCTAACGAGTGA
CCGCCGGACCGCAATGGTAAGTCCCACTCCCTGCGTGACCAGGTGTAGTTGGGCGCGGCGCGGACAACCGGCG
ACATTAAGGAATGCACTGTGAAACTGCGAATGGCTCA

>Acyl__ETS

GGAGGTGTTGGTTGGAGCCCCGGGAAAAAAAAAATTTCCCCCCCGAGCTGGTGAAAGGTCGGCAAAGACCCACA
CGTCTCCCTTGTAGTGCCTAATGCATGAGAGCGTGTGG**GCATGTCCCGATGAGGCCCCATTGAGATCACAACATC**
ATATAGGGGCGGAGGGATTGGCTCTCGATCACCCTATTGGCACGGGTGATCGTGGGCGACGGGCTGGGCGACG
GCTGAGCGCTCGCTGGGCGCTCGCTGGGCGATACATGGGCCGGTGATTCGGCGTATCACCATGAGAGAATTCCT
TGCCATCATCTATGGCACGTCACGAGATGTTGTTGGCTGAATGGTTGGGCGCATGGGCATAGCTTGTGGGAGAT
CCCCGGGCAATTGGTGGGCATTGGATGGTTGGCGGATATCTTGTGAATGTTTGAGATCACCACGTGGAGCGTTGC
TCGGGCTGGTGCCGAGCAGCCCCAAATATGTGAGTTGGGTACTTTTTGTGGACGGTTGTCAATCCTCTCATGCTTG
GTAGCTATGAAATGAAGGGGGTTTACCATGCCTCTCCCTTGTATAACTTGCCCCGATATTATCAGGCGACTGCGTTT
GTCTCCCATGCAGCATCTTAGTCCGTATATCGCTCAATCCGGTTACATTCGTTTGTAACGCTGCGGCGCTGGTGGCC
AAAGCGGGCGATGGAATAAGGTGTTGCATGGGGGAAATCATGGGCTGTTACTTTGATGCGGGGGAACCTTCAGG
ATATTGCTACTAGATTGCATTTTGGGAAGACTACCGTGCCCAATAACCCAGTTTGCACCCATCCGGCTCCACGTAGT
GCAAGCACAAAATGTCCATGAGATGCCTGATACTGTTCCGAAGC**TTTTCATATTGGTTTGAAACTTTTGCCCCGAT**
AGTGTGTCCCGACTGTTGTATCACCTGATTTCGCGCACAGCGAGCGTTTTGAATGGCGCCTCGTTCTACCATATGT
ATTGCCCTTTGCGGGGTGATGCACACATGGGTGAGCGGGCATTGTTCCCAACGCAACGTGTTGGCGTGTGAGTG
GTAGTAGAGACACCTCTGCTTTTGGGCTCCGTGCTTTGCGCATCGAACCATAAGCACATGTTTCCCTTATTAGCAT
CCGGCGTACTTGATTGTATACTGGCAAGTAAAATGGTTCCTGTGTTGCCTACCCGTAGAAATTGGA**AAATATCAT**
TGCTAACGAGATTCATTGCCTTTAGCCGGCCAAAAGCTGGCCGAGGGCAACATGTTAGTGTGGCTTGTGAAACC
TACCGCGGCGTTTGCCCGCGTGTCTGGCTAGCAAGCTTGCACGTGCTTGGGGATATGGAAAACATAGATGGGTC
AGGTTTCGAACTCGGCCGATGCAATTGTAGCTAATTTTGTGACCGCCGGACCGTAATGATAAGTCCTACTCCCTG
CGTGACCAGGTGTAGTTAGGCGCGGCACGGACAGTCGGCGACATAAAGGAATGCCGACATAAAGGAATGCTAC
CTGG
