# Supplementary material for: Structure and Methylation of 35S rDNA in Allopolyploids Anemone multifida (2n = 4x = 32, BBDD) and Anemone baldensis (2n = 6x = 48, AABBDD) and Their Parental Species Show Evidence of Nucleolar Dominance
Source: Front Plant Sci. 2022 Jul 6;13:908218. doi: 10.3389/fpls.2022.908218 (PMC9296772; doi:10.3389/fpls.2022.908218)
Supplement: Supplementary file 2 [file Image_2.pdf]

Supplementary Figure S2. The consensus 35S rDNA sequences *Anemone cylindrica, A. multifida, A. baldensis*-LC variant*, A. baldensis*-HC variant*, A. sylvestris* (variants 1, 2 and 3), *A. parviflora*-HC variant*, A. parviflora*-LC variant. Transcription initiation region (TIS) is highlighted in yellow.

>A_cylindrica/1-10489

ACTACAACACAACAATTGCTGGGTTTAGACCTTGAGGTTACAACTACAATTCTATTTTATGAGAGAGTGATACTTAG
AAAAAATTATAAGTCTGGGAAATAAAAACTCCACTTCATATGTAAGTGCATCTCGTGGACAAACATGGTCGAGTAT
CGCCCGGGCAAGGATGCATGCCCATCACCCGGGCAAATATCTTCGACAGTGCTGACGATGCTTGTGAGATGGATC
ACCGACCCCGATGTAGCATATTGCTGGGGCAAATATGCCTTGGTAGCTTTAATGATACCGGAGGAATATGCCTTGG
ATCATCCAGGGAAATATGCCCGAGAGTAGCCTAGCATGTGGGCAAATATGCATCGACAGCCCCGACGATGTTTGT
GAGATGGATCAACCGAGGATATGTCACGGGATAGCGTATCCCGTGAAAATATGCACAATGAGATGAATCACGCAT
TCAAATAACCCCTGTCATGATGATGCTTGTGAAATGGGCAACTATTGGGCATTTCATGGGCATCATTTGGGCATGA
GCTGGGCATTTGCTGGCCGCCATGTATCGCCCGCATGGCTTGCACCAGCGCCCGTAGCGTATCGCCATGTATCGCC
CGCATGGCTTGCACTTGCGCCCGTAGCGTATCGCCATGTATCGCCCGCATGGCTTGGGCATTTCATAGGCATCTTTT
GGGCGTGAGCTGGGCATTTGATGGCCGCCATGTATCGCCCGCATGGCTTGCACCATCGCCCGTAGCGTATCGCCA
TGTATCGCCCGCATGGCTTGCTTTAGCGCCCGTAGCGTATCGCCATGTATCGCCCGCATGGCTTGCACCAGCTTCC
GTTGCGTATCGCCATGTATCGCCCGCATGTAGCGTATCGCCATGTATCGCCGCATGGCTTGCACTAGCGCCCGTAG
CGTATCGCCATGTATCGCCGCATGGCTTGCACTAGCGCCCGTAGCGTATCGCCATGTATCGCCGCATGGCTTGCAC
CATAGCGTATCGCCATGTATCGCCCGCATGTCTTGCACCAGCTCCCGTAGCGTATCGCCATGTATCGCCCGCATGG
CTTGCACTTGAGCCCGTAGCGTATCGCCATGTATTGCCCGCATGGCTTGGGCATTTCATAGGCATCTTTTGGGCGT
GAGCTGGGCATTTGATGGCCGCCATGTATCGCCCGCATGGCCCGTAGCGTATCGCCATGTATCGCCCGCATGGCTT
GCACTAGCGCCCGTAGCGTATCGCCATGTATCGCCCGTAGCGTATCGCCATGTATCGCCCGCATGGCTTGCACCAG
CTCTCGTTGCGTATCGCCATGTATCGCCCGCATGGCTTGGGCATTTGTTGGGCATCCTTGGGCATTGGCTAGGCGT
CTCTTGGGCATTGGCTGGGCGCTATGCATCCCCGGGCAAGATCACTCGGGGAATGAGACCTATGCATCGTCTGGT
CAAGTATGGCTATGGTTCTCTCGGGCAACCTCAACTATGCATCACCACATAAGAGATATTAAGGTTGCATGGGTGA
TGCTATTGAGGCTGCGTGGGCGATGCATGGCCAGGCCGACGTGCATCCGGGAGGAAATTTCTTGAAAACACGTCT
AAATGGTCCAATTTTTTTGTGACAACTTAGTAGTGGGTAGTACTCATAAAAACACACCTAAGGCAAAAAGATTGAG
AAAAAAAGAGGTCCGGGGAAAAAAAAATCGCAAAAAATCGTTTTCGTCAATTCTGAGTATGAATACTCCATTATCAG
CTTCCAAAACATGTCTAGATGTGTAGTACGTTCGAAAACACCCCAAATGAAAAAAAAAAGTGTCAAAAAACGACAC
CGGAGTGAGAAGTTATGACATTTTGAATTTCAGTGTGCCCCAATGTTCCCTTTTCAGTACCCTGGAAAAATTTTAGT
GGCTATATTAGGGGGGAGGTGTTGGTTGGAGCCCCGGGAAAAAAAAAATTTCCCCCCCGAGCTGGTGAAAGGCT
AGCAATGCCCACACGATGCATCGGCCAATGCATGTGAGCGTGTGGGCATGTCCCGATGAGGTCCCTTTTGAGTTTC
CAACACCGAACAAACCACGTGGGGGCGTGGGAATGGCTCTTGATCACCCTATTGGCACGGGCGATCCTGGGCGA
CGGGTGGGCGATGGATGGGTGCTTGCTGGGTGATACATGGGCTGGTGATTCAGCGTATCATCATAGGAGAATTCC
TTACCATCGTCCATGGCACGTCACGAGATGGCATTGGAAATAGGGGGATGCGTTGGTTGATGGCTTCTTGGACAA
GAAAATCCCCCCGAGCTGGTGAAAGGTCGGCAAAGACCCACACGTCTCCCTTGTAGTGCCTAATGCATGAGAGCG
TGTGGGCATGTCCCGATGAGGCCCCATTGAGATCACAACATCATATAGGGGCGGAGGGATTGGCTCTCGATCACC
CTATTGGCACGGGTGATCGTGGGCGACGGGCTGGGCGACGGCTGAGCGCTCGCTGGGCGCTCGCTGGGCGCTCG
CTGGGTGCTTGCTGGGCGCTCGCTGGGCGATACATGGGCCGGTGATTCGGCGTATCACCATGAGAGAATTCCTTG
CCATCATCTATGGCACGTCACGAGATGTTGTTGGCTGAATGGTTGGGCGCATGGGCATAGCTTGTGGGAGATCCC
CGGGCAATTGGTGGGCATTGGATGGTTGGCGGATATCTTGTGAATGTTTGAGATCACCACGTGGAGCGTTGCTCG
GGCTGGTGCCGAGCAGCCCCAAATATGTGAGTTGGGTACTTTTTGTGGACGGTTGTCAATCCTCTCATGCTTGGTA
GCTATGAAATGAAGGGGGTTTACCATGCCTCTCCCTTGTATAACTTGCCCCGATATTATCAGGCGACTGCGTTTGTC
TCCCATGCAGCATCTTAGTCCGTATATCGCTCATCCTAATTACATTCGTTTGTAATACTGTGGCGCAGGTGGCCAAA

GCGGGTGATGGAATAAGGTGTTGCATGGGGGTACACTATAGGTCTCCCATGCAGCATCTTAGTCCGTATATCGCTC
AATCCGGTTACATTCGTTTGTAACGCTGCGGCGCTGGTGGCCAAAGCGGGCGACGGAATAGGTTGTTGCATGGGG
GTACGCATGGTCTTCCATGCAGCATCTTAGTTCGTATATTGCTCACCCCAGTTACATTTGTTTGTAACGCTGCGGCG
CTGGTGGCCAAAGCGAGCAATGGAAAAAGTTGTTGCATGGTGGATCATGTGTGCGTCTGCTCGCATGCACCATCT
TAGGCCTTGATTGCTCAGTCCGGTTACATTCGTTTGTAACGCTGTGGCGCTGGTGGCCAAAGCGGGCGATGGAAT
AAGGTGTTGCATGTGGGAAGGTCGTGGGCTGTTACTTTGATGTTGGGGGTTCACCATGCCTCTCTCCTGGATAACC
TTCCCGTTCCATTCCACGCGGCCATTTGACTTGTTTAAGGCGCTTCATGTGCGAAAAAAACATGGTCATCTCAACGA
GTGGTTTAGGATAGGGGGTGTGATGGGGGAGGAGTGTGTTGCTGCCCACTATGCATCAGCTTGGCCCGATACTAT
CAGGCGACCGTGTTAGTCTCCCATGCAGCATCTTAGTCCGTATATCGCTCAATCCGGTTACATTCGTTTGTAACGCT
GCGGCGCTGGTGGCCAAAGCGGGGCGGTGGAATAGGTTGTTGCATGGGGGTGCGCATGGTCTCCCATGAAACAT
CCATCTTAGTCCGTATATCGCTCATCTTGGTTACATTCGTTTGTAACGCTGTGGCACTGGTGGCCAAAGCGGGCGAT
GGAATAAGGTGTTTCATGGGGGATCATGCGTACGTCTGCTCCCATGCACCATTTTAGGCCTTGATCGCTCAAATCG
GTTACATTTTTGTTTGTAACGCTGCGGCGCTGGTGGCCAAAGCGGGCGATGGAATAAGGTGTTGCATGGGGGAAA
TCATGGGCTGTTACTTTGATGCGGGGGAACCTTCAGGATATTGCTACTAGATTGCATTTTGGGAAGACTACCGTGC
CCAATAACCCAGTTTGCACCCATCCGGCTCCACGTAGTGCAAGCACAAAATGTCCATGAGATGCCTGATACTGTTC
CGAAGCTTTTCATATTGGTTTGAAACTTTTGCCCCGATAGTGTGTCCCGACTGTTGTATCACCTGATTTCGCGCACA
GCGAGCGTTTTGAATGGCGCCTCGTTCTACCATATGTATTGCCCTTTGCGGGGTGATGCACACATGGGTGAGCGG
GCATTGTTCCCAACGCAACGTGTTGGCGTGTGAGTGGTAGTAGAGACACCTCTGCTTTTGGGCTCCGTGCTTTGCG
CATCGAACCATAAGCACATGTTTCCCTTATTAGCATCCGGCGTACTTGATTGTATACTGGCAAGTAAAATGGTTCCT
GTGTTGCCTACCCGTAGAAATTGGAAAATATCATTGCTAACGAGATTCATTGCCTTTAGCCGGCCAAAAGCTGGCC
GAGGGCAACATGTTAGTGTGGCTTGTGAAACCTACCGCGGCGTTTGCCCGCGTGTCTGGCTAGCAAGCTTGCACG
TGCTTGGGGATATGGAAAACATAGATGGGTCAGGTTTCGAACTCGGCCGATGCAATTGTAGCTAATTTTGTGACC
GCCGGACCGTAATGATAAGTCCTACTCCCTGCGTGACCAGGTGTAGTTAGGCGCGGCACGGACAGTCGGCGACAT
AAAGGAATGCTACCTGGTTGATCCTGCCAGTAGTCATATGCTTGTCTCAAAGATTAAGCCATGCATGTGTAAGTAT
GAACTAATTCAGACTGTGAAACTGCGAATGGCTCATTAAATCAGTTATAGTTTGTTTGATGGTATATGCTACTCGG
ATAACCGTAGTAATTCTAGAGCTAATACGTGCAACAAACCCCGACTTTTGGAAGGGATGCATTTATTAGATAAAAG
GTCAACGCGGGCTTGTCCCGTTGCTCTGATGATTCATGATAACTCGACGGATCGCACGGCCTTTGTGCCGGCGACG
CATCATTCAAATTTCTGCCCTATCAACTTTCGATGGTAGGATAGTGGCCTACTATGGTGGTGACGGGTGACGGAGA
ATTAGGGTTCGATTCCGGAGAGGGAGCCTGAGAAACGGCTACCACATCCAAGGAAGGCAGCAGGCGCGCAAATT
ACCCAATCCTGACACGGGGAGGTAGTGACAATAAATAACAATACCGGGCTCTTAGAGTCTGGTAATTGGAATGAG
TACAATCTAAATCCCTTAACGAGGATCCATTGGAGGGCAAGTCTGGTGCCAGCAGCCGCGGTAATTCCAGCTCCAA
TAGCGTATATTTAAGTTGTTGCAGTTAAAAAGCTCGTAGTTGGACTTTGGGTTTGGTAGGCCGGTCCGCCCTTGTG
GTGTGCACCGGTCGACCAGTCCCTTCTACCGGCGATACGCTCCTGTCCTTAATTGGCCGGGTCGTGCCACCGGTGC
TGTTACTTTGAAGAAATTAGAGTGCTCAAAGCAAGCCCAAGCTCTGGATATATTAGCATGGGATAACATCATAGGA
TTTCGGTCCTATTGCGTTGGCCTTCGGGATCGGAGTAATGATTAACAGGGACAGTCGGGGGCATTCGTATTTCATA
GTCAGAGGTGAAATTCTTGGATTTATGAAAGACGAACCACTGCGAAAGCATTTGCCAAGGATGTTTTCATTAATCA
AGAACGAAAGTTGGGGGCTCGAAGACGATCAGATACCGTCCTAGTCTCAACCATAAACGATGCCGACCAGGGATC
GGCGGATGTTGCTTTTAGGACTCCGCCGGCACCTTATGAGAAATCAAAGTTTTTGGGTTCCGGGGGGAGTATGGT
CGCAAGGCTGAAACTTAAAGGAATTGACGGAAGGGCACCACCAGGAGTGGAGCCTGCGGCTTAATTTGACTCAA
CACGGGGAAACTTACCAGGTCCAGACATAGTAAGGATTGACAGACTGAGAGCTCTTTCTTGATTCTATGGGTGGT
GGTGCATGGCCGTTCTTAGTTGGTGGAGCGATTTGTCTGGTTAATTCCGTTAACGAACGAGACCTCAGCCTGCTAA
CTAGCTATGTGGAGGTACCCTCCACGGCCAGCTTCTTAGAGGGACTATGGCCGTTTAGGCCACGGAAGTTTGAGG
CAATAACAGGTCTGTGATGCCCTTAGATGTTCTGGGCCGCACGCGCGCTACACTGATGTATTCAACGAGTCTATAG
CCTTGGCCGACAGGCCCGGGTAATCTTTGAAAATTTCATCGTGATGGGGATAGATCATTGCAATTGTTGGTCTTCA

ACGAGGAATTCCTAGTAAGCGCGAGTCATCAGCTCGCGTTGACTACGTCCCTGCCCTTTGTACACACCGCCCGTCG
CTCCTACCGATTGAATGGTCCGGTGAAATGTTCGGATCGCGGCAACGTGGGTGGTTCGCCGCCGGCGACGCCGCG
AGAAGTCCATTGAACCTTATCATTTAGAGGAAGGAGAAGTCGTAACAAGGTTTCCGTAGGTGAACCTGCGGAAGG
ATCATTGTCGATACCTGCTCAGCAGAACGACCCACGAACACGTGAAAACAACCTACCATGCCCGGGGAGCGGGCT
CTGGCCAGCGACCCGGTGCTGCAACAAAATTCGGCGCAACTGGCGTCAAGGAAAACTCACCGGAAGCAAGGCGT
CGGCTCGTTCGGCGCCGCGTGTCCGAATACTCAAACGACTCTCGGCAACGGATATCTCGGCTCTTGCATCGATGAA
GAACGTAGCGAAATGCGATACTTGGTGTGAATTGCAGAATCCCGTGAACCATCGAGTCTTTGAACGCAAGTTGCG
CCCGAAGCCTTTTTGGCCGAGGGCACGCCTGCCTGGGCGTCACACACAGCGTCGCCCCCCACCAATCCCTCTGGAT
GGGATGGGGATGGGGGCGGAGATTGGCCCCCCGAGCCCTCTGGTGCACGGTCGGCATAAATGTTGGTCCCCGGC
GGCGAGCGTCGCGGTCAGCGGTGGTTGTATACTCATCCCCCGAGGACAAAATGACGCGCACGCCTCGTCGCTCGT
CGGGCAGAGCAAACCCCTGGGAGCACGGCTTCCACCTGCGACCCCAGGTCAGGCGGGATTACCCGCTGAGTTTAA
GCATATCAATAAGCGGAGGAAAAGAAACTTACGAGGATTCCCCTAGTAACGGCGAGCGAACCGGGAATAGCCCA
GCTTGGGAATCGGGCGACCCCGTCGTCCGAATTGTAGTCTGTAGAAGCGTCCTCAGCGGCGGACCGGGCCCAAGT
CCCCTGGAAGGTGGCGCCAGAGAGGGTGAGAGCCCCGTCGTGCCCGGACCCTGTCGCACCACGAGGCGCTGTCG
GCGAGTCGGGTTGTTTGGGAATGCAGCCCCAATCGGGCGGTAAATTCCGTCCAAGGCTAAATACTGGCGAGAGA
CCGATAGCGAACAAGTACCGCGAGGGAAAGATGAAAAGGACTTTGAAAAGAGAGTCAAAGAGTGCTTGAAATTG
TCGGGAGGGAAGCGGATGGGGGTCGGCGATGTGTGCCGGTCGGATGCGGAACGGCGAGAGCTGGTCCGCCGCT
CGGCTCGGTGCGCGGACCGACGCGGATTGTGGAGGCGACCCAAGCCTGGGCCTCCTCGGAGGCCTGCGGAGATG
TCGTCCCCGCGATTGTGGTGGGCAGCACGCGCCTCACGGCGTGCCTCGGCAACTGCGTGCTCCCGGCGTCGGCCA
GTGGGCTCCCCATTCGGCCCGTCTTGAAACACGGACCAAGGAGTCTGACATGTGTGCGAGTCAGCGGGTGAGTAA
ACCCGTGAGGCGCAAGGAAGCTAATTGGCGGGATCCCCCTGTGGGTTGCACCGCCGACCGACCTAGATCTTCTGT
GAAGGGTTCGAGTGTGAGCATACCTGTCGGGACCCGAAAGATGGTGAACTATGCCTGAGCGGGGCGAAGCCAGA
GGAAACTCTGGTGGAGGCCCGCAGCGATACTGACGTGCAAATCGTTCGTCTGACTTGGGTATAGGGGCGAAAGA
CTAATCGAACCATCTAGTAGCTGGTTCCCTCCGAAGTTTCCCTCAGGATAGCTGGAGCTCGCGGGCGAGTTCTATC
AGGTAAAGCCAATGATTAGAGGCATCGGGGGCGCAACGCCCTCGACCTATTCTCAAACTTTAAATAGGTAGGACG
GCGCGGCTACTTTGCTGAGCCGCGCCACGGAATCGAGTGCTCCAAGTGGGCCATTTTTGGTAAGCAGAACTGGCG
ATGCGGGATGAACCGGAAGCCGGGTTACGGTGCCCAACTGCGCGCTAACCCAGAACCCACAAAGGGTGTTGGTC
GATTAAGACAGCAGGACGGTGGTCATGGAAGTCGAAATCCGCTAAGGAGTGTGTAACAACTCACCTGCCGAATCA
ACTAGCCCCGAAAATGGATGGCGCTGAAGCGCGCGACCCACACCCGGCCGTCAGGGCAATTGTTAGGCCCTGATG
AGTAGGAGGGCGCAGCGGTGGCCGCGAAACCCAGGGCGCGAGCCCGGGTGGAGCCGCCGTTGGTGCAGATCTT
GGTGGTAGTAGCAAATATTCAAATGAGAACTTTGAAGGCCGAAGAGGGGAAAGGTTCCATGTGAACGGCACTTG
CACATGGGTTAGTCGATCCTAAGAGACGGCCGAAAGGCGTCAGAGAGCGTGCATCACGCGAGCTTCGAAAGGGA
ATCGGGTTAAAATTCCTGAACCGGGACACAGTGGTCGACGGCAACGCTAGGAAGTCCGGAGACGTCGGCGGGGG
CCTCGGGAAGAGTTATCTTTTCTGTTTAACAGCCTGCCCACCCTGGAAACGGCTCAGCCGGAGGTAGGGTCCAGC
GGCTGGAAGAGCACCGCACGTCGCGTGGTGTCCGGTGCGCCCCCGGCGGCCCTTGAAAATCCGGAGGACCGAAT
GCCAACTGTGCCCGGTCGTACTCATAACCGCATCAGGTCTCCAAGGTGAACAGCCTCTGGTCGATGGAACAATGTA
GGCAAGGGAAGTCGGCAAAATGGATCCGTAACCTCGGGAAAAGGATTGGCTCTGAGGGCTGGGCACGGGGGTC
CCAGTCCCGAACCCGTTGGCTGCCGGCGGACTGCTCGAGCTGCTTCCGTGGCGAGAGCGGGTCGCCGCGTGCCG
GCCGGGGGATGGACTGGGAACGGCTCCTTCGGGGACCTTCCCCGGGCGTCGAACAGCCAACTCAGAACTGGTAC
GGACAAGGGGAATCCGACTGTTTAATTAAAACAAAGCATTGCGATGGTCCCTGCGGATGCTAACGCAATGTGATT
TCTGCCCAGTGCTCTGAATGTCAAAGTGAAGAAATTCAACCAAGCGCGGGTAAACGGCGGGAGTAACTATGACTC
TCTTAAGGTAGCCAAATGCCTCGTCATCTAATTAGTGACGCGCATGAATGGATTAACGAGATTCCCACTGTCCCTGT
CTACTATCCAGCGAAACCACAGCCAAGGGAACGGGCTTGGCAGAATCAGCGGGGAAAGAAGACCCTGTTGAGCT
TGACTCTAGTCCGACTTTGTGAAATGACTTGAGAGGTGTAGGATAAGTGGGAGCCGACTCGTGCGGCGAAAGTG

AAATACCACTACTTTTAACGTTATTTTACTTATTCCGTGAATCGGAAGCGGGGCACTGCTCCTCTTTTTGGATCCAA
GTTCGGTCTCGACGGGACAATCCGGGCGGAAGACATTGTCAGGTGGGGAGTTTGGCTGGGGCGGCACATCTGTT
AAAAGATAACGCAGGTGTCCTAAGATGAGCTCAACGAGAACAGAAATCTCGTGTGGAACAAAAGGGTAAAAGCT
CGTTTGATTCTGATTTCCAGTACGAATACGAACCGTGAAAGCGTGGCCTATCGATCCTTTAGACCTTCGGAATTTGA
AGCTAGAGGTGTCAGAAAAGTTACCACAGGGATAACTGGCTTGTGGCAGCCAAGCGTTCATAGCGACGTTGCTTT
TTGATCCTTCGATGTCGGCTCTTCCTATCATTGTGAAGCAGAATTCACCAAGTGTTGGATTGTTCACCCACCAATAG
GGAACGTGAGCTGGGTTTAGACCGTCGTGAGACAGGTTAGTTTTACCCTACTGATGATAGTGTCGCGATAGTAATT
CAACCTAGTACGAGAGGAACCGTTGATTCACACAATTGGTCATCGCGCTTGGTTGAAAAGCCAGTGGCGCGAAGC
TACCGTGTGCAGGATTATGACTGAACGCCTCTAAGTCAGAATCCAGGCTAGAGAAGCGACGCATGCGCCCGTCGC
CCGATTGCCGACCTGCAGTAGGGGTCCTTGGACCCCCAAAGGCACGTGTCATTGGCGTAGCTCCCGTGGCCGATG
AGTCGCGTGGTGCCGCCTTGAAGTATAATTCCTACCGAGCGGCGGGCTGAATCCTTTGCAGACGACTTAAATACGC
GACGGGGTATTGTAAGTGGCAGAGTGGCCTTGCTGCCACGATCCACTGAGATTCAGCCCCATGTCGCATCGATTC
GTCCCTCCCCATCCAAAAAA

>A_multifida/1-10715

CAACAACTCACCAATTGCCCCGTATACCCCTCGAGGTTACAACCTGCCCCGATAATATTCTACGCCAAGTGCGAGG
GATGCTTACTTAGAGAAGAAAAAAAAACTCTAAGTATGGGATACATACAAAAACTCCACGTAGACACAGAGCCAA
GTGCCGGGGCATCTCCCGGACAAATACAAATCGAGTTTGTCGCCGGGGCAAGGATGCATGCCCATCACCCCGCGG
CAACGATGATGCCTATGTACAACCGACGGTGCTTCTGCAGACGAGGCAAGTGCATATGCATCGCTATCTATCACTT
GGGCAAGATCAACTTGCAAGGACGCATACGCATCACCGGGGCAATGATGGATAATCGATTATCACCCGGGGTAAT
ACGCCCCGACGTAGCATACCGTCCTGGCAAATATCCCCCGGCAACCCCGACGGTGCTTCTGAGATGGATCACCAAC
CCCGACATTCCATATCACCCGGGCGATATTATTCTACGGAGCCCCGACTGGGCTTCTGAGAGGTATCACCCCGAAG
GAAAAATGCCCCGGCGTAGCATACCGTCCGGGCAAATATCCCCCGGCAATGGTGACGGTGCTTCTGAGATGGATC
ACCAACCCCGACATTCCATATCACCCGGGCGATATTATTCTACGGAGCCCCGACTGGGCTTCTGAGAGGTATCACC
CCGAAGGAAAAATGCCCCGACGTAGCATACCGTCCGGGCAAATATGCCTCGGTATCTCCTACGATGCTTGTGAGA
TGGATCACACAGGGGAATATGCCCCAGCAGATATATCGACGGTGCTTTTGAGATGGATCAACCCGGGGTAATATG
TCCCGGGTAGCATATCCCCAAGCAAATACGCTCACCCGGGGGTGCTGTGAGTTTGATCACCCGGGGTAATATGCC
CCGGCGTAGCATATCACCCGGAGGAATATCCACCTAGAGTCTTAACAGTGCTTGTGAGAAGGATCAGATGGGAAA
ATATGCCCGTATCGTACCGCCGTGTACCGCATGGCCATGCACTAGCCCCCATAGCGTATCGCCGTGTACCGCATGG
CCATGCACTAGCCCCCGTAGCGTATCGCCGTGTAACGCATGGCTTGCACTAGCCCCCGTAGCGTATCGCCGTGTAC
CGCATGGCTATACGCGCCGCCGTAGCGCCCGTCGTGTACCGCATGGCTTGCACTAGTGCCCCGTAGCGTATGGCC
ATGCACTAGCCCTGGTAGCGTATCCCCGTGTACCGCATGGCCGGGGGCTAGCGCGTACCGTATGGCCATGCACTA
GCCCCCGTAGCGTATCGCCGTGTACTAGCATGGCCATCCACTAGCGCCCGATGCGTATCGCCGTGTACCACATGGC
CATGCACTTGCGCCCGATGCGTATCGCCGTGTACCGCATGGCCATGCACTAGCCCCCGCAGCGTATCGCCGTGTAC
CGCATGGCCATGCACTGGCGCCCGATGCGTATCGCCGTGTACCGCATGGCCATGCACTAGCCCCCGCAGCGTATC
GCCGTGTACCGCATGGCCATGCACTAGCGCGCGTAGCGTATCGCCATGTACCGCATGGCCATGCACTAGCGCCCG
ATGCGTATCGCCGTGTACCGCATGGCCATGCACTAGCGCCCATTGCGTATCGCCGTGTCGAGGGCCATGTATCGCC
CGGGCAAGATCATCCGGTGAATGAGGCCTATGCATCGTCGGGTTAAATATGGCTTGAGTTCTCTCGGGCAACCTC
AACCATGCATCACCATGCTACAGCTACTGAGGCTGCGTGGGTGATGCTACTGAGGCTGCGTGGGCAATGCATGGC
CCGGTCGGCGTGCGCCCGGTAGGCATTTTCTTAAAAACACACCGAAATGCTCCAATTTTTTTTTAACAACTTGGCAG
TGGTTAGTACTCATAAAAACACACCCAAGGAAAAAAGATTGACAAAAAAAGAGGTTCGTACAAAAATTTTCGAAA
AAAATCGTTTTCGTCAATTCTGAGTATGAATACCCCATTATCCGCTTCCAAAACCTGTCTAGATGTGTAGTACGTTC
GAAAACACCCCAAATGAAAAAAAAAAGTGTCAAAAAACGATACCGGAGTGAGAAGTTATGTCATTTTCAATTTCA

GTGCGCCCCAATGTTCCCTTTTCAGTACCAGGGAAAAATTTTAGTGGCTATATTAGGGGGGAGGTGTTGGTTGAA
GCTCCCAGGAAAAAAAAAAATTCCCCCCCGAGCTGGTGAAAGGCCAACAATGCCCACACGTCTCCCATGCATCCCCC
AATGCATGTGAGCGTGTGGGCATGTCGCAATGAGGTCCCTTTCGAGTTTACAACACCGAACAAACCACGTGGGGG
CGTGGGAATGGCTCTCGATCACCCTATTGGCACGGGTGATGGCTGGGCGATGGATGGGCGATGATGGGCGTTCT
GTGGGCGTTCTGTGGGCGATACATGGTCCGGTGATTCGACGTATCGCCATAAGAGAATTCCTTACCATCGTCCATG
GCACGTCACGAGATGGTGTTGGAAATAGTTGGCTACTAGGAAAAAAACCATTGGATGGACCGTCGTGAGAACGG
GTTGGTTGGATGGTTGGGCGCATGGGCGCATCCTGGGAGATCCCCGGGCAATCGATGGGCATTGGATGGTTATC
GGACATCTTGTGAACGTATGTGATCACCATGTGGAGCGTTGCTCGGGCAGGTGCTGAGCAACCCCAAATATGCGA
GCTGGGTACTTTTTGTGGACGGTTGTCAATCCTCATGCTTGGTAGCCATGAAATGAAGGGGGTTCACCATGCCTCT
CCCTCGGATAACCTTTTGAGCCCATACCACATGGTCATTTGACATGTTTAATCTGCTTCATGTGCGGAAAAAAACAT
GGTCTTCTCAACGAGTGGCTTAGGCTTGGGGGTGTGAGGGGGGAGGAGTGTGGTGCTGCCCACGATGCATCAAC
TTGCCCCGATACTATTAGATGATCGTGTTCGTCTCCCATGCAACATCTTAGTCCGTATATCGCTCATCCTAATTACAT
TCGTATGTAACGCTGCGGCGCAGGTGGCCAAAGCGGGCGATGGAATAAGGTGTTGCATGGGGGCTTTATAGGTC
TCCCATGCACCATCTTAGGCCTTTTGATCGCTCAATCCGGTTACATTCATTTGTAACGCTGCGGCGCTGGTGGCCAA
AGCGAGCGATGGAATAAGGTGTTGCATGGGGGAAATGTAGGGCTCCCATGCAACATTTTAGGCCGTGATCGCTCA
ATTCGGTTACATTCGTTTGTAATGCTGCGGTGCTGGTGGCCAAAGCGGGGCGATGGAATGAGGTGTTGCATGGG
GGTACTCTAGGTCTCACATGCAACATCTTAGTCCGTATATCGCTCATCCCGATTACATTCGTTTGTAACGCTGTGGT
GCTGGTGGCCAAAGCGAGCGATGGAATAAGGTGTTGCATGGTGGATCATGCGTACGTCTGCTCCCATGCACCATC
TTAGGCCTTGATCGCTCAATCCGGTTACACATTGGCTTGTAACGCTGCGGCGCTGGTGGCCAAAGCGGGCGATGG
AATAAGGTGTTGCATTGGGGAATATCATGGGCTACTACTTTGATGCTTGGGGGGTTCACTATGCCTCTCCCCCGGA
TAACCTTCCCGGCCCATTCCACACGGCTATTTGACTTGTTTAAGCCACTTCATGTGCGAAAAAAACATGGTCATCTC
AACGAGTGGTTTAGGATAGGGGGTGTGAGGGGGGAGGAGTGTGTTGCTACCCACTATGCATCAACTTGCCCTGA
TACTATCAGACGACCGTGTTAGTCTCCCATGCAGCATCTTAGTCCGTATATCGCTCAATCCGGTTACATTTGTTTGTA
ACGCTGCGGCGCTGGTGGCCAAAGTGGGGCGATGGAATGAGGTGTTGCATGGGGTACTCCAGGTCTCCCATGCA
ACATCTTAGTCCGTACATCGCTCAATCCGGTTACATTCGTTTGTAACGCTACGGCGCTGGTGGCCAAAGCGGGCGA
TGGAATAGGTTGTTGCATGGGGGTACGCATGGTCTCCCATGCAACATCTTAGTCCGTATATCGCTTATCCTGGTTAC
ATTCGTTTGTAACGCTGCGGTGCTGGTGGCCAAAGCGGGCGATGGAATAAGGTGTTGCATGGTGGATCATGCGTA
CGTTTGCTCCCATGTACCATCATGGGGTGTTACTTTGATGCTGGGACCTTCAGGGCATTGCTACTAGATTGCGTGTT
GGGGACACTCCCGTGCCCAATAACCCAGTTTGCACCCATCCGGCTCCACGTAGTGCAATGAAAAAATGTTCACGAG
ATGCCCCGATACTGTTCTGACGCTTTTCATGTTGGTTTGAAACTTGCCCCGACAGTGTGTCCCGATCGTTACAACTA
GATTTTGCGCACAGCGAGCGTTTTGAACGACGCTTCGTTCTACCATATGTATTGCCCTTTGCGGGGTGATGCACAC
ATATGGGTGAGCAGGCATTGTTCCCAACGCAACGTGTTGGCGTGTGAGTGGTAGTAGAGACATCCCTGCTTTTGG
GCTCCGTGCTTCGCGCATCGAACCATAAGCATATGTTTCCCTCATTGGCATCCGGCGTACTTGATTGTACGTCGGCA
AGTAAAATGGTTCCTGTGCTCCCTACCCGAAGAAAGTGGAAAAGATCATTGCTAACGAGATTTATTGCCTTTGGTC
GGCCAAAAGCTGGCCGAGGGCAACATGTTAGTGTGGCTTGTGAAACCAACCGCGGCGTTTGCCCGCGTGTCTGGT
TAGCGAGCTCGCACGTGCTTGGGGATATGGAAAACATAGATGGGTCAGGGTTCTGAACTCGACCGACGCAATCGT
AGCTAATGAGTGACCGCCGGACTGCAATGGTAAGTCCCACTCCCTGCGTGACCAGGTGTAGTTGGGCACGGCGCG
GAGAACCGGCGACATGAAGGAATGCTACCTGGTTGATCCTGCCAGTAGTCATATGCTTGTCTCAAAGATTAAGCC
ATGCATGTGTAAGTATGAACTAATTCAGACTGTGAAACTGCGAATGGCTCATTAAATCAGTTATAGTTTGTTTGAT
GGTATATGCTACTCGGATAACCGTAGTAATTCTAGAGCTAATACGTGCAACAAACCCCGACTTCTGGAAGGGATGC
ATTTATTAGATAAAAGGTCAACGCGGGCTTGTCCCGTTGCTCTGATGATTCATGATAACTCGACGGATCGCACGGC
CTTTGTGCCGGCGACGCATCATTCAAATTTCTGCCCTATCAACTTTCGATGGTAGGATAGTGGCCTACTATGGTGGT
GACGGGTGACGGAGAATTAGGGTTCGATTCCGGAGAGGGAGCCTGAGAAACGGCTACCACATCCAAGGAAGGC
AGCAGGCGCGCAAATTACCCAATCCTGACACGGGGAGGTAGTGACAATAAATAACAATACCGGGCTCTTAGAGTC

TGGTAATTGGAATGAGTACAATCTAAATCCCTTAACGAGGATCCATTGGAGGGCAAGTCTGGTGCCAGCAGCCGC
GGTAATTCCAGCTCCAATAGCGTATATTTAAGTTGTTGCAGTTAAAAAGCTCGTAGTTGGACTTTGGGTTTGGTAG
GCCGGTCCGCCCTTGTGGTGTGCACCGGTCGACCAGTCCCTTCTACCGGCGATACGCTCCTGTCCTTAATTGGCCG
GGTCGTGCCACCGGTGCTGTTACTTTGAAGAAATTAGAGTGCTCAAAGCAAGCCCAAGCTCTGGATATATTAGCAT
GGGATAACATCATAGGATTTCGGTCCTATTGCGTTGGCCTTCGGGATCGGAGTAATGATTAACAGGGACAGTCGG
GGGCATTCGTATTTCATAGTCAGAGGTGAAATTCTTGGATTTATGAAAGACGAACCACTGCGAAAGCATTTGCCAA
GGATGTTTTCATTAATCAAGAACGAAAGTTGGGGGCTCGAAGACGATCAGATACCGTCCTAGTCTCAACCATAAAC
GATGCCGACCAGGGATCGGCGGATGTTGCTTTTAGGACTCCGCCGGCACCTTATGAGAAATCAAAGTTTTTGGGTT
CCGGGGGGAGTATGGTCGCAAGGCTGAAACTTAAAGGAATTGACGGAAGGGCACCACCAGGAGTGGAGCCTGC
GGCTTAATTTGACTCAACACGGGGAAACTTACCAGGTCCAGACATAGTAAGGATTGACAGACTGAGAGCTCTTTCT
TGATTCTATGGGTGGTGGTGCATGGCCGTTCTTAGTTGGTGGAGCGATTTGTCTGGTTAATTCCGTTAACGAACGA
GACCTCAGCCTGCTAACTAGCTATGTGGAGGTACCCTCCACGGCCAGCTTCTTAGAGGGACTATGGCCGTTTAGGC
CACGGAAGTTTGAGGCAATAACAGGTCTGTGATGCCCTTAGATGTTCTGGGCCGCACGCGCGCTACACTGATGTA
TTCAACGAGTCTATAGCCTTGGCCGACAGGCCTGGGTAATCTTTGAAAATTTCATCGTGATGGGGATAGATCATTG
CAATTGTTGGTCTTCAACGAGGAATTCCTAGTAAGCGCGAGTCATCAGCTCGCGTTGACTACGTCCCTGCCCTTTGT
ACACACCGCCCGTCGCTCCTACCGATTGAATGGTCCGGTGAAATGTTCGGATCGCGGCAACGTGGGTGGTTCGCC
GCCGGCGACGCCGCGAGAAGTCCATTGAACCTTATCATTTAGAGGAAGGAGAAGTCGTAACAAGGTTTCCGTAGG
TGAACCTGCGGAAGGATCATTGTCGATACCTGCTCAGCAGAACGACCTGCGAACACGTGAAAACAACTTACCATG
CCCGGGGAGCGGGCTACGGCCAGCGACCCGGTGCAACAACAAAATTCGGCGCAACTGGCGTCAAGGAAAACTTA
TCGGAAGCAAGGCGTCGGCTCGTTCGGCGTCGCGTATCCAAATACTCAAACGACTCTCGGCAATGGATATCTCGG
CTCTTGCATCGATGAAGAACGTAGCGAAATGCGATACTTGGTGTGAATTGCAGAATCCCGTGAACCATCGAGTCTT
TGAACGCAAGTTGCGCCCGAAGCCTTTTTGGCCGAGGGCACGCCTGCCTGGGCGTCACACACAGCGTCGCCCCCA
CTAATCCCTCTGGATTGGATGGGGGCGGAGATTGGCCCCCCGAGCCCTCCGGGGCACGGTCGGCATAAATGTGG
GTCCTCGGCGATGAGCGTCGCGGTCAGCGGTGGTTGTACACTCATCCCCCAAAGACGAAATGACGCGCACGCCTC
GTCGCTCGGCGGACAGAGAAAACCCCAGGAAGCCGGGCTTCCACCTGCGACCCCAGGTCAGGCGGGATTACCCG
CTGAGTTTAAGCATATCAATAAGCGGAGGAAAAGAAACTTACGAGGATTCCCCTAGTAACGGCGAGCGAACCGG
GAACAGCCCAGCTTGGGAATCGGGCGACTTCGTCGTCCGAATTGTAGTCTGTAGAAGCGTCCTCAGCGGCGGACC
GGGCCCAAGTCCCCTGGAAGGTGGCGCCAGAGAGGGTGAGAGCCCCGTCGTGCCCGGACCCTGTTGCACCACGA
GGCGCTGTCGGCGAGTCGGGTTGTTTGGGAATGCAGCCCCAATCGGGCGGTAAATTCCGTCCAAGGCTAAATACT
GGCGAGAGACCGATAGCGAACAAGTACCGCGAGGGAAAGATGAAAAGGACTTTGAAAAGAGAGTCAAAGAGTG
CTTGAAATTGTCGGGAGGGAAGCGGATGGGGGCCGGCGATGTGCACCGGTCGGATGCGGAACGGCGTTAGCTG
GTCCGCCGCTCGGCTCGGTGCGCGGACCGACGCGGATTGTGGAGGCGACCCAAGCCTGGGCCTCTTCGGAGGCC
CGTGGAGATGTCGTCCCCGCGATTGTGGTGGGCAGCACGCGCCTCACGGCGTGCCTCGGCAACTGCGTGCTCCCG
GCGTCGGCCTGTGGGCTCCCCATTCGGCCCGTCTTGAAACACGGACCAAGGAGTCTGACATGTGTGCAAGTCAGC
GGGTGAGTAAACCCGTGAGGCGCAAGGAAGCTAATTGGCGGGATCCCCCTGTGGGTTGCACCGCCGACCGACCT
AGATCTTCTGTGAAGGGTTCGAGTGTGAGCATACCTGTCGGGACCCGAAAGATGGTGAACTATGCCTGAGCGGG
GCGAAGCCAGAGGAAACTCTGGTGGAGGCCCGCAGCGATACTGACGTGCAAATCGTTCGTCTGACTTGGGTATA
GGGGCGAAAGACTAATCGAACCATCTAGTAGCTGGTTCCCTCCGAAGTTTCCCTCAGGATAGCTGGAGCTCGCGG
GCGAGTTCTATCAGGTAAAGCCAATGATTAGAGGCATCGGGGGCGCAACGCCCTCGACCTATTCTCAAACTTTAAA
TAGGTAGGACGGTGCGGCTACTTTGCTGAGCCGTGCCACGGAATCGAGTGCTCCAAGTGGGCCATTTTTGGTAAG
CAGAACTGGCGATGCGGGATGAACCGGAAGCCGGGTTACGGTGCCCAACTGCGCGCTAACCCAGAACCCACAAA
GGGTGTTGGTCGATTAAGACAGCAGGACGGTGGTCATGGAAGTCGAAATCCGCTAAGGAGTGTGTAACAACTCA
CCTGCCGAATCAACTAGCCCCGAAAATGGATGGCGCTGAAGCGCGCGACCCACACCCGGCCGTCAGGGCAATTGT
TAGGCCCTGATGAGTAGGAGGGCGCAGCGGTGGCCGCGAAACCCAGGGCGCAAGCCCGGGTGGAGCCGCCGTT

GGTGCAGATCTTGGTGGTAGTAGCAAATATTCAAATGAGAACTTTGAAGGCCGAAGAGGGAAAGGTTCCATGT
GAACGGCACTTGCACATGGGTTAGTCGATCCTAAGAGACGGCCGAAAGGCGTCAGAGAGCGTGCATCACGCGAG
CTTCGAAAGGGAATCGGGTTAAAATTCCTGAACCGGGACACAGTGGTCGACGGCAACGTTAGGAAGTCCGGAGA
CGTCGGCGGGGGCCTCGGGAAGAGTTATCTTTTCTGTTTAACAGCCTGCCCACCCTGGAAACGGCTCAGCCGGAG
GTAGGGTCCAGCGGCTGGAAGAGCACCGCACGTCGCGTGGTGTCCGGTGCGCCCCCGGCGGCCCTTGAAAATCC
GGAGGACCGAATGCCAACTGTGCCCGGTCGTACTCATAACCGCATCAGGTCTCCAAGGTGAACAGCCTCTGGTCG
ATGGAACAATGTAGGCAAGGGAAGTCGGCAAAATGGATCCGTAACCTCGGGAAAAGGATTGGCTCTGAGGGCTG
GGCACGGGGGTCCCAGTCCCGAACCCGTTGGCTGCCGGCGGACTGCTCGAGCTGCTTCCGTGGCGAGAGCGGGT
CGCCGCGTGCCGGCCGGGGGATGGACTGGGAACGGCCCCTTCGGGGGCCTTCCCCGGGCGTCGAACAGCCAACT
CAGAACTGGTACGGACAAGGGGAATCCGACTGTTTAATTAAAACAAAGCATTGCGATGGTCCCTGCGGATGCTAA
CGCAATGTGATTTCTGCCCAGTGCTCTGAATGTCAAAGTGAAGAAATTCAACCAAGCGCGGGTAAACGGCGGGAG
TAACTATGACTCTCTTAAGGTAGCCAAATGCCTCGTCATCTAATTAGTGACGCGCATGAATGGATTAACGAGATTC
CCACTGTCCCTGTCTACTATCCAGCGAAACCACAGCCAAGGGAACGGGCTTGGCAGAATCAGCGGGGAAAGAAG
ACCCTGTTGAGCTTGACTCTAGTCCGACTTTGTGAAATGACTTGAGAGGTGTAGGATAAGTGGGAGCCGACTCGT
GCGGCGAAAGTGAAATACCACTACTTTTAACGTTATTTTACTTATTCCGTGAATCGGAAGCGGGGCACTGCTCCTCT
TTTTGGATCCAAGTTCGGTCTCGACGGGACAATCCGGGCGGAAGACATTGTCAGGTGGGGAGTTTGGCTGGGGC
GGCACATCTGTTAAAAGATAACGCAGGTGTCCTAAGATGAGCTCAACGAGAACAGAAATCTCGTGTGGAACAAAA
GGGTAAAAGCTCGTTTGATTCTGATTTCCAGTACGAATACGAACCGTGAAAGCGTGGCCTATCGATCCTTTAGACC
TTCGGAATTTGAAGCTAGAGGTGTCAGAAAAGTTACCACAGGGATAACTGGCTTGTGGCAGCCAAGCGTTCATAG
CGACGTTGCTTTTTGATCCTTCGATGTCGGCTCTTCCTATCATTGTGAAGCAGAATTCACCAAGTGTTGGATTGTTC
ACCCACCAATAGGGAACGTGAGCTGGGTTTAGACCGTCGTGAGACAGGTTAGTTTTACCCTACTGATGACAGTGT
CGCGATAGTAATTCAACCTAGTACGAGAGGAACCGTTGATTCACACAATTGGTCATCGCGCTTGGTTGAAAAGCCA
GTGGCGCGAAGCTACCGTGTGCAGGATTATGACTGAACGCCTCTAAGTCAGAATCCAGGCTAGAGAAGCGACGC
ATGCGCCCGCCGCCCAATTGCCGACCTGCAGTAGGGGTCCTCGGACCCCCAAAGGCACGTGTCATTGGCGTAGCT
CCCGCGGCCGACGAGCCGCGTGGTGCCGCCTTGAAGTATAATTCCTACTGAGCGGCGGGCTGAATCCTTTGCAGA
CGACTTAAATACGCGACGGGGTATTGTAAGTGGCAGAGTGGCCTTGCTGCCACGATCCACTGAGATTCAGCCCCA
TGTCGCATCGATTCGTCCCTCCCCATCCAAAAAA

>A_baldensis_LC/1-10761

CAACAACTCACCAATTGCCCCGTATACCCCTCGAGGTTACAACCTGCCCCGATAATATTCTACGCCAAGTGCGAGG
GATGCTTACTTAGAGAAGAAAAAAAAACTCTAAGTATGGGATACATACAAAAACTCCACGTAGACACAGAGCCAA
GTGCCGGGGCATCTCCCGGACAAATACAAATCGAGTTTGTCGCCGGGGCAAGGATGCATGCCCATCACCCCGCGG
CAACGATGATGCCTATGTACAACCGACGGTGCTTCTGCAGACGAGGTAAGTGCATATGCATCGCTATCTATCACTT
GGGCAAGATCAACTTGCAAGGACGCATACGCATCACCGCGGCAATGATGGATAATCGATTATCACCCGGGGAAAT
ATGCCCCTGGAGTAGCGAGGGTGCTGTGAGATGGATCACCCGGGGTAATACGCCCCGACGTAGCATACCGTCCTG
GCAAATATCCCCCGGCAACCCCGACGGTGCTTCTGAGATGGATCACCAACCCCGACATTCCATATCACCCGGGCGA
TATTATTCTACGGAGCCCCGACTGGGCTTCTGAGAGGTATCACCCCGAAGGAAAAATGCCCCGGCGTAGCATACC
GTCCGGGCAAATATCCCCCGGCAATGGTGACGGTGCTGGTGAGATGGATCACCAACCCCGACGTAGCATATCACC
CGGGCAAATATGCAACGGAGCCCCGACTGGGCTTCTGAGATGTATCACCCCGAAAAATATGCCCCGGCGTAGCAT
ATCGCCCGGGAAAATATGCCTCGGTATCTCCTACGATGCTTGTGAGATGGATCACACAGGGGAATATGCCCCAGC
AGATATATCGACGGTGCTTTTGAGATGGATCAACCCGGGGTAATATGTCCCGGGTAGCATATCCCCAAGCAAATA
CGCTCACCCGGGGGTGCTGTGAGTTTGATCACCCGGGGTAATATGCCCCGGCGTAGCATATCACCCGGAGGAATA
TCCACCTAGAGTCTTAACAGTGCTTGTGAGAAGGATCAGATGGGAAAATATGCCCGTATCGTACCGCCGTGTACC

GCATGGCCATGCACTAGCCCCATAGCGTATCGCCGTGTACCGCATGGCCATGCACTAGCCCCGTAGCGTATCGC
CGTGTAACGCATGGCTTGCACTAGCCCCGTAGCGTATCGCCGTGTACCGCATGGCTATACGCGCCGCCGTAGCGC
CCGTCGTGTACCGCATGGCTTGCACTAGTGCCCCGTAGCGTATGGCCATGCACTAGCCCTGGTAGCGTATCCCCGT
GTACCGCATGGCCGGGGGCTAGCGCGTACCGTATGGCCATGCAGTAGCCCCCGTAGCGTATCGCCGTGTACTAGC
ATGGCCATCCACTAGCGCCCGATGCGTATCGCCGTGTACCACATGGCCATGCACTTGCGCCCGATGCGTATCGCCG
TGTACCGCATGGCCATGCACTAGCCCCCGCAGCGTATCGCCGTGTACCGCATGGCCATGCACTGGCGCCCGATGC
GTATCGCCGTGTACCGCATGGCCATGCACTAGCCCCCGCAGCGTATCGCCGTGTACCGCATGGCCATGCACTAGCG
CGCGTAGCGTATCGCCATGTACCGCATGGCCATGCACTAGCGCCCGATGCGTATCGCCGTGTACCGCATGGCCAT
GCACTAGCGCCCATTGCGTATCGCCGTGTCGAGGGCCATGTATCGCCCGGGCAAGATCATCCGGTGAATGAGGCC
TATGCATCGTCGGGTTAAATATGGCTTGAGTTCTCTCGGGCAACCTCAACCATGCATCACCATGCTACAGCTACTGA
GGCTGCGTGGGTGATGCTACTGAGGCTGCGTGGGCAATGCATGGCCCGGTCGGCGTGCGCCCGATAGGCATTTT
CTTAAAAACACACCGAAATGCTCCAATTTTTTTTTAACAACTTGGCAGTGGTTAGTACTCATAAAAACACACCCAAG
GAAAAAAGATTGACAAAAAAAGAGGTTCGTACAAAAATTTTCGAAAAAAATCGTTTTCGTCAATTCTGAGTATGAA
TACCCCATTATCCGCTTCCAAAACCTGTCTAGATGTGTAGTACGTTCGAAAACACCCCAAATGAAAAAAAAAAGTG
TCAAAAAACGATACCGGAGTGAGAAGTTATGTCATTTTCAATTTCAGTGCGCCCCAATGTTCCCTTTTCAGTACCAG
GGAAAAATTTTAGTGGCTATATTAGGGGGGAGGTGTTGGTTGAAGCTCCCAGGAAAAAAAAAATTCCCCCCCGAG
CTGGTGAAAGGCCAACAATGCCCACACGTCTCCCATGCATCCCCCAATGCATGTGAGCGTGTGGGCATGTCGCAAT
GAGGTCCCTTTCGAGTTTACAACACCGAACAAACCACGTGGGGGCGTGGGAATGGCTCTCGATCACCCTATTGGC
ACGGGTGATGGATGGGCGATGGATGGGCGATGATGGGCGTTCTGTGGGCGTTCTGTGGGCGATAGATGGTCCG
GTGATTCGACGTATCGCCATAAGAGAATTCCTTACCATCGTCCATGGCACGTCACGAGATGGTGTTGGAAATAGTT
GGCTACTAGGAAAAAAACCATTGGATGGACCGTCGTGAGAACGGGTTGGTTGGATGGTTGGGCGCATGGGCGCA
TCCTGGGAGATCCCCGGGCAATCGATGGGCATTGGATGGTTATCGGACATCTTGTGAACGTATGTGATCACCATGT
GGAGCGTTGCTCGGGCAGGTGCTGAGCAACCCCAAATATGCGAGCTGGGTACTTTTTGTGGACGGTTGTCAATCC
TCATGCTTGGTAGCCATGAAATGAAGGGGGTTCACCATGCCTCTCCCTCGGATAACCTTTTGAGCCCATACCACAC
GGTCATTTGACATGTTTAATCTGCTTCATGTGCGGAAAAAAACATGGTCTTCTCAACGAGTGGCTTAGGCTTGGGG
GTGTGAGGGGGGAGGAGTGTGGTGCTGCCCACGATGCATCAACTTGCCCCGATACTATTAGATGATCGTGTTCGT
CTCCCATGCAACATCTTAGTCCGTATATCGCTCATCCTAATTACATTCGTATGTAACGCTGCGGCGCAGGTGGCCAA
AGCGGGCGATGGAATAAGGTGTTGCATGGGGGCTTTATAGGTCTCCCATGCACCATCTTAGGCCTTTTGATCGCTC
AATCCGGTTACATTCATTTGTAACGCTGCGGCGCTGGTGGCCAAAGCGAGCGATGGAATAAGGTGTTGCATGGGG
GAAATGTAGGGCTCCCATGCAACATTTTAGGCCGTGATCGCTCAATTCGGTTACATTCGTTTGTAATGCTGTGGTG
CTGGTGGCCAAAGCGGGGCGATGGAATGAGGTGTTGCATGGGGGTACTCTAGGTCTCACATGCAACATCTTAGTC
CGTATATCGCTCATCCCGATTACATTCGTTTGTAACGCTGTGGTGCTGGTGGCCAAAGCGAGCGATGGAATAAGGT
GTTGCATGGTGGATCATGCGTACGTCTGCTCCCATGCACCATCTTAGGCCTTGATCGCTCAATCCGGTTACACATTG
GCTTGTAACGCTGCGGCGCTGGTGGCCAAAGCGGGCGATGGAATAAGGTGTTGCATTGGGGAATATCATGGGCT
ACTACTTTGATGCTTGGGGGGTTCACTATGCCTCTCCCCCGGATAACCTTCCCGGCCCATTCCACACGGCTATTTGA
CTTGTTTAAGCCACTTCATGTGCGAAAAAAACATGGTCATCTCAACGAGTGGTTTAGGATAGGGGGTGTGAGGGG
GGAGGAGTGTGTTGCTACCCACTATGCATCAACTTGCCCTGATACTATCAGACGACCGTGTTAGTCTCCCATGCAG
CATCTTAGTCCGTATATCGCTCAATCCGGTTACATTTGTTTGTAACGCTGCGGCGCTGGTGGCCAAAGTGGGGCGA
TGGAATGAGGTGTTGCATGGGGTACTCCAGGTCTCCCATGCAACATCTTAGTCCGTACATCGCTCAATCCGGTTAC
ATTCGTTTGTAACGCTACGGCGCTGGTGGCCAAAGCGGGCGATGGAATAGGTTGTTGCATGGGGGTACGCATGG
TCTCCCATGCAACATCTTAGTCCGTATATCGCTTATCCTGGTTACATTCGTTTGTAACGCTGCGGTGCTGGTGGCCA
AAGCGGGCGATGGAATAAGGTGTTGCATGGTGGATCATGCGTACGTTTGCTCCCATGTACCATCATGGGGTGTTA
CTTTGATGCTGGGACCTTCAGGGCATTGCTACTAGATTGCGTGTTGGGGACACTCCCGTGCCCAATAACCCAGTTT
GCACCCATCCGGCTCCACGTAGTGCAATGAAAAAATGTTCACGAGATGCCCCGATACTGTTCTGACGCTTTTCATG

TTGGTTTGAAACTTGCCCCGACAGTGTGTCCCGATCGTTACAACTAGATTTTGCGCACAGCGAGCGTTTTGAACGA
CGCTTCGTTCTACCATATGTATTGCCCTTTGCGGGGTGATGCACACATATGGGTGAGCAGGCATTGTTCCCAACGC
AACGTGTTGGCGTGTGAGTGGTAGTAGAGACATCCCTGCTTTTGGGCTCCGTGCTTCGCGCATCGAACCATAAGCA
TATGTTTCCCTCATTGGCATCCGGCGTACTTGATTGTACGTCGGCAAGTAAAATGGTTCCTGTGCTCCCTACCCGAA
GAAAGTGGAAAAGATCATTGCTAACGAGATTTATTGCCTTTGGTCGGCCAAAAGCTGGCCGAGGGCAACATGTTA
GTGTGGCTTGTGAAACCAACCGCGGCGTTTGCCCGCGTGTCTGGTCAGCGAGCTCGCACGTGCTTGGGGATATGG
AAAACATAGATGGGTCAGGGTTTTGAACTCGACCGACGCAATCGTAGCTAATGAGTGACCGCCGGACTGCAATGG
TAAGTCCCACTCCCTGCGTGACCAGGTGTAGTTGGGCACGGCGCGGAGAACCGGCGACATGAAGGAATGCTACCT
GGTTGATCCTGCCAGTAGTCATATGCTTGTCTCAAAGATTAAGCCATGCATGTGTAAGTATGAACTAATTCAGACT
GTGAAACTGCGAATGGCTCATTAAATCAGTTATAGTTTGTTTGATGGTATATGCTACTCGGATAACCGTAGTAATTC
TAGAGCTAATACGTGCAACAAACCCCGACTTCTGGAAGGGATGCATTTATTAGATAAAAGGTCAACGCGGGCTTG
TCCCGTTGCTCTGATGATTCATGATAACTCGACGGATCGCACGGCCTTTGTGCCGGCGACGCATCATTCAAATTTCT
GCCCTATCAACTTTCGATGGTAGGATAGTGGCCTACTATGGTGGTGACGGGTGACGGAGAATTAGGGTTCGATTC
CGGAGAGGGAGCCTGAGAAACGGCTACCACATCCAAGGAAGGCAGCAGGCGCGCAAATTACCCAATCCTGACAC
GGGGAGGTAGTGACAATAAATAACAATACCGGGCTCTTAGAGTCTGGTAATTGGAATGAGTACAATCTAAATCCC
TTAACGAGGATCCATTGGAGGGCAAGTCTGGTGCCAGCAGCCGCGGTAATTCCAGCTCCAATAGCGTATATTTAA
GTTGTTGCAGTTAAAAAGCTCGTAGTTGGACTTTGGGTTTGGTAGGCCGGTCCGCCCTTGTGGTGTGCACCGGTCG
ACCAGTCCCTTCTACCGGCGATACGCTCCTGTCCTTAATTGGCCGGGTCGTGCCACCGGTGCTGTTACTTTGAAGAA
ATTAGAGTGCTCAAAGCAAGCCCAAGCTCTGGATATATTAGCATGGGATAACATCATAGGATTTCGGTCCTATTGC
GTTGGCCTTCGGGATCGGAGTAATGATTAACAGGGACAGTCGGGGGCATTCGTATTTCATAGTCAGAGGTGAAAT
TCTTGGATTTATGAAAGACGAACCACTGCGAAAGCATTTGCCAAGGATGTTTTCATTAATCAAGAACGAAAGTTGG
GGGCTCGAAGACGATCAGATACCGTCCTAGTCTCAACCATAAACGATGCCGACCAGGGATCGGCGGATGTTGCTT
TTAGGACTCCGCCGGCACCTTATGAGAAATCAAAGTTTTTGGGTTCCGGGGGGAGTATGGTCGCAAGGCTGAAAC
TTAAAGGAATTGACGGAAGGGCACCACCAGGAGTGGAGCCTGCGGCTTAATTTGACTCAACACGGGGAAACTTAC
CAGGTCCAGACATAGTAAGGATTGACAGACTGAGAGCTCTTTCTTGATTCTATGGGTGGTGGTGCATGGCCGTTCT
TAGTTGGTGGAGCGATTTGTCTGGTTAATTCCGTTAACGAACGAGACCTCAGCCTGCTAACTAGCTATGTGGAGGT
ACCCTCCACGGCCAGCTTCTTAGAGGGACTATGGCCGTTTAGGCCACGGAAGTTTGAGGCAATAACAGGTCTGTG
ATGCCCTTAGATGTTCTGGGCCGCACGCGCGCTACACTGATGTATTCAACGAGTCTATAGCCTTGGCCGACAGGCC
CGGGTAATCTTTGAAAATTTCATCGTGATGGGGATAGATCATTGCAATTGTTGGTCTTCAACGAGGAATTCCTAGT
AAGCGCGAGTCATCAGCTCGCGTTGACTACGTCCCTGCCCTTTGTACACACCGCCCGTCGCTCCTACCGATTGAATG
GTCCGGTGAAATGTTCGGATCGCGGCAACGTGGGTGGTTCGCCGCCGGCGACGCCGCGAGAAGTCCATTGAACC
TTATCATTTAGAGGAAGGAGAAGTCGTAACAAGGTTTCCGTAGGTGAACCTGCGGAAGGATCATTGTCGATACCT
GCTCAGCAGAACGACCCGCGAACACGTGAAAACAACCTACCATGCCCGGGAGCGGGCTCCGGCCAGCGACCCGG
TGCTGCAACAAAATCCGGCGCAACTGGCGTCAAGGAAAACTCATCGGAAGCAAGGCGTCGGCTCGTTCGGCGCC
GCGTGTCCGAATACTCAAACGACTCTCGGCAACGGATATCTCGGCTCTTGCATCGATGAAGAACGTAGCGAAATG
CGATACTTGGTGTGAATTGCAGAATCCCGTGAACCATCGAGTCTTTGAACGCAAGTTGCGCCCGAAGCCTTTTTGG
CCGAGGGCACGCCTGCCTGGGCGTCACACACAGCGTCGCCCCCCACCAATCCCTCTGGATGGGATGGGGGGCGG
AGATTGGCCCCCCGAGCCCTCTGGGGCACGGTCGGCATAAATGTTGGTCCCCGGCGGCGAGCGTCGCGGTCAGC
GGTGGTTGTATACTCATCCCCCGAGGACAAAATGACGCGCACGCCTCGTCGCTCGTCGGCAGAGCAAACCCCAG
GGAGCACGGCTTCCACCTGCGACCCCAGGTCAGGCGGGATTACCCGCTGAGTTTAAGCATATCAATAAGCGGAGG
AAAAGAAACTTACGAGGATTCCCCTAGTAACGGCGAGCGAACCGGGAATAGCCCAGCTTGGGAATCGGGCGACC
CCGTCGTCCGAATTGTAGTCTGTAGAAGCGTCCTCAGCGGCGGACCGGGCCCAAGTCCCCTGGAAGGTGGCGCCA
GAGAGGGTGAGAGCCCCGTCGTGCCCGGACCCTGTCGCACCACGAGGCGCTGTCGGCGAGTCGGGTTGTTTGGG
AATGCAGCCCCAATCGGGCGGTAAATTCCGTCCAAGGCTAAATACTGGCGAGAGACCGATAGCGAACAAGTACCG

CGAGGGAAAGATGAAAAGGACTTTGAAAAGAGAGTCAAAGAGTGCTTGAAATTGTCGGGAGGGAAGCGGATGG
GGGCCGGCGATGTGCACCGGTCGGATGCGGAACGGCTTCAGCTGGTCCGCCGCTCGGCTCGGTGCGCGGACCGA
CGCGGATTGTGGAGGCGACCCAAGCCTGGGCCTCTTCGGAGGCCCGTGGAGATGTCGTCCCCGCGATTGTGGTG
GGCAGCACGCGCCTCACGGCGTGCCTCGGCAACTGCGTGCTCCCGGCGTCGGCCTGTGGGCTCCCCATTCGGCCC
GTCTTGAAACACGGACCAAGGAGTCTGACATGTGTGCAAGTCAGCGGGTGAGTAAACCCGTGAGGCGCAAGGAA
GCTAATTGGCGGGATCCCCCTGTGGGTTGCACCGCCGACCGACCTAGATCTTCTGTGAAGGGTTCGAGTGTGAGC
ATACCTGTCGGGACCCGAAAGATGGTGAACTATGCCTGAGCGGGGCGAAGCCAGAGGAAACTCTGGTGGAGGCC
CGCAGCGATACTGACGTGCAAATCGTTCGTCTGACTTGGGTATAGGGGCGAAAGACTAATCGAACCATCTAGTAG
CTGGTTCCCTCCGAAGTTTCCCTCAGGATAGCTGGAGCTCGCGGGCGAGTTCTATCAGGTAAAGCCAATGATTAGA
GGCATCGGGGGCGCAACGCCCTCGACCTATTCTCAAACTTTAAATAGGTAGGACGGCGCGGCTACTTCGCTGAGC
CGCGCCACGGAATCGAGTGCTCCAAGTGGGCCATTTTTGGTAAGCAGAACTGGCGATGCGGGATGAACCGGAAG
CCGGGTTACGGTGCCCAACTGCGCGCTAACCCAGAACCCACAAAGGGTGTTGGTCGATTAAGACAGCAGGACGG
TGGTCATGGAAGTCGAAATCCGCTAAGGAGTGTGTAACAACTCACCTGCCGAATCAACTAGCCCCGAAAATGGAT
GGCGCTGAAGCGCGCGACCCACACCCGGCCGTCAGGGCAATTGTTAGGCCCTGATGAGTAGGAGGGCGCAGCGG
TGGCCGCGAAACCCAGGGCGCGAGCCCGGGTGGAGCCGCCGTTGGTGCAGATCTTGGTGGTAGTAGCAAATATT
CAAATGAGAACTTTGAAGGCCGAAGAGGGGAAAGGTTCCATGTGAACGGCACTTGCACATGGGTTAGTCGATCCT
AAGAGACGGCCGAAAGGCGTCAGAGAGCGTGCATCACGCGAGCTTCGAAAGGGAATCGGGTTAAAATTCCTGAA
CCGGGACACAGTGGTCGACGGCAACGCTAGGAAGTCCGGAGACGTCGGCGGGGGCCTCGGGAAGAGTTATCTTT
TCTGTTTAACAGCCTGCCCACCCTGGAAACGGCTCAGCCGGAGGTAGGGTCCAGCGGCTGGAAGAGCACCGCAC
GTCGCGTGGTGTCCGGTGCGCCCCCGGCGGCCCTTGAAAATCCGGAGGACCGAATGCCAACTGTGCCCGGTCGTA
CTCATAACCGCATCAGGTCTCCAAGGTGAACAGCCTCTGGTCGATGGAACAATGTAGGCAAGGGAAGTCGGCAAA
ATGGATCCGTAACCTCGGGAAAAGGATTGGCTCTGAGGGCTGGGCACGGGGGTCCCAGTCCCGAACCCGTTGGC
TGCCGGCGGACTGCTCGAGCTGCTTCCGTGGCGAGAGCGGGTCGCCGCGTGCCGGCCGGGGGATGGACTGGGA
ACGGCTCCTTCGGGGGCCTTCCCCGGGCGTCGAACAGCCAACTCAGAACTGGTACGGACAAGGGGAATCCGACT
GTTTAATTAAAACAAAGCATTGCGATGGTCCCTGCGGATGCTAACGCAATGTGATTTCTGCCCAGTGCTCTGAATG
TCAAAGTGAAGAAATTCAACCAAGCGCGGGTAAACGGCGGGAGTAACTATGACTCTCTTAAGGTAGCCAAATGCC
TCGTCATCTAATTAGTGACGCGCATGAATGGATTAACGAGATTCCCACTGTCCCTGTCTACTATCCAGCGAAACCAC
AGCCAAGGGAACGGGCTTGGCAGAATCAGCGGGGAAAGAAGACCCTGTTGAGCTTGACTCTAGTCCGACTTTGT
GAAATGACTTGAGAGGTGTAGGATAAGTGGGAGCCGACTCGTGCGGCGAAAGTGAAATACCACTACTTTTAACGT
TATTTTACTTATTCCGTGAATCGGAAGCGGGGCACTGCTCCTCTTTTTGGATCCAAGTTCGGTCTCGACGGGACAAT
CCGGGCGGAAGACATTGTCAGGTGGGGAGTTTGGCTGGGGCGGCACATCTGTTAAAAGATAACGCAGGTGTCCT
AAGATGAGCTCAACGAGAACAGAAATCTCGTGTGGAACAAAAGGGTAAAAGCTCGTTTGATTCTGATTTCCAGTA
CGAATACGAACCGTGAAAGCGTGGCCTATCGATCCTTTAGACCTTCGGAATTTGAAGCTAGAGGTGTCAGAAAAG
TTACCACAGGGATAACTGGCTTGTGGCAGCCAAGCGTTCATAGCGACGTTGCTTTTTGATCCTTCGATGTCGGCTCT
TCCTATCATTGTGAAGCAGAATTCACCAAGTGTTGGATTGTTCACCCACCAATAGGGAACGTGAGCTGGGTTTAGA
CCGTCGTGAGACAGGTTAGTTTTACCCTACTGATGATAGTGTCGCGATAGTAATTCAACCTAGTACGAGAGGAACC
GTTGATTCACACAATTGGTCATCGCGCTTGGTTGAAAAGCCAGTGGCGCGAAGCTACCGTGTGCAGGATTATGAC
TGAACGCCTCTAAGTCAGAATCCAGGCTAGAGAAGCGACGCATGCGCCCGTCGCCCGATTGCCGACCTGCAGTAG
GGGTCCTTGGACCCCCAAAGGCACGTGTCATTGGCGTAGCTCCCGTGGCCGATGAGTCGCGTGGTGCCGCCTTGA
AGTATAATTCCTACCGAGCGGCGGGCTGAATCCTTTGCAGACGACTTAAATACGCGACGGGGTATTGTAAGTGGC
AGAGTGGCCTTGCTGCCACGATCCACTGAGATTCAGCCCCATGTCGCATCGATTCGTCCCTCCCCATCCAAAAAA

>A_baldensis_HC/1-10939

AACTACAACACACCAATTGCCTCGTATACCACTCGAGGTTACAACTATTTTACGAGAGAGTGATACTTGGAAAAAA
TTCTAAGTCTGGGAAATAAATACTCCACTTCATATGTAAGTGCCATCGCCCGGACAAACCATGGTCGAGTATCATC
GCCAGTAGGGCTTGTACGAGCGCCCGTAGCGTATCACTATGTATAGTGAACATGGCTTGCACTAGCTCCCGTAGC
GTATTGCCATGTATCACCCGCATGGCTTGCACTAGCCCCTGTAACGTATCGCCATGTATCGCCCGTAGCGTATCGCC
ATGTATCGCCCGCATGGCTTGCACTAGCCTGTGGGCAAATATGCATCGACTTCCCCGACGATGTTTGTGAGATGGA
TCAACCGAGGAAATATGTCACGGGATAGCATATCCCCAAATAAATATGCACAATGGCATGAATCACCTATGCAAAT
AACCCCTGTCATGATGATGCTTGTGAAATGGGCAACTATTGGGCATTTCATAGGCATCGTTTGGGCATGAACTGGG
CATTTGCTGGCCACCATGTATCGCCCGCATGCCTTGTACTAGCGCCCGTAGCGTATCGCCATGTATCACCCGCATGG
CTTGCACTAGCTCCCGTCGCGTATCGCCATGTATCGCCCGTAGTGTATCGCCATGTATCACCCGCATGGCTTGCACC
AGCTCCCGTAGCGTATCGCCATGTATCGACCGTAGTGTATCGCCATGTATCACCCGCATGGCTTGCACCAGCTCCC
GTAGCGTATCGCCATGTATCGCCCGTAGCGTATCGCCATGTATTGCCCGCATAGCTTGGGCAATTCCTAGGCATCTT
TTGGGCGTGTGCTGGGCATTTGATAGCCGTCATGTATCGCCCGCATGGCTTGCACTAGCTCCCGTAGCGTATCGCC
ATGTATCGCCCGTAGCGTATCGCCATGTATCACCCGCATGGCTTGCACCAGCTCCCGTAGCGTATCGCCATGTATC
GCCCGCATGGCTTACACCAGCGCCCGTAGCGTATCGCCATGTATCGCCCGCATGGCTTGCACCAGCGCCCGTAGCG
TATCGCCATGTATCGCCCGTAGCGTATCGCCATGTATCACCCGCATGGCTTGCACCACCTCCCGTAGCGTATCGCCA
TGTATCGCACACATGGCTTGCACTAGCGCCCGTAGCGTATCGCCATGTATTGCCTGCATGGCTTGGGCATTTCATA
GGCATCTCTTGGGCGTGAGCTGGGCATTTGATGGCCGCCATGTATCGCCCGCATGGTTGCACCCGCGCCCGTAGC
GTATCGCCATGTATCGCCCGTAGCGCCCTCGCCATGTATCACCCGCATGGCTTGCACCAGCTCCCGTAGCGTATCG
CCATGTATCGCACGCATGGCTTGCACTAGCGCCCGTAGCGTATCGCCATGTATTGCCCGCATGGCTTGGGCATTTC
ATAGGCATCTTTTGGGCGTGAGTTGGGCATTTGATGGCCGCCATGTATCGCCCGCATGGCTTGCACCAGCTCCCGT
AGCGTATCACCATGTATCGCCCGCATGGCTTGCACTAGCGCCCGTAGCGTATCACCATGTATCGCCCGTAGCGTAT
CGCCATGTATCGACCGCATGGCTTGCACTAGCGCCCATAGCGTATCGCCATGTATCGCCCGTAGCGTATCGCCATG
TATCACCCGCATGGCTTGCACCAGCTCCCGTAGCGTATCGCCATGTATCGCCCGCATGGCTTGCACTAGCGCCCGT
AGCGTATCGCCATGTATCGCCCGCATGGCTTGGGCATTTGCTGGGCATCCTTGGGCATTGGCTAGGCGTTGCTTGG
GCATTGGCTGGGCGCTATGCATCCCCGGGCAAGATCACTCGGGGAATGAGACCTATGCATCGTCTGGTCAAGTAT
GGTTATGGTTCTCTCGGGGAACCTCAACCATGCATCACCACATAAGAGATATTGAGGTTGCATGGGTGATTCTATT
GAGGCTGCGTGGGCGATGCATGGCTAGGCCGACGTGCATCCGGGAGGAAAATTCTTGAAAACACATCTAAATGG
TCCAATTTTTTTGTGACAACTTAGTAGTGGCTATTACTCATAAAAACACACCTAAGGCAAAAAGATTGAGAAAAAA
AGAGGTCCGGGGAAAAAAAATCGTAAAAAATCATTTTCGTCAATTCTGAGTATGAATACTCCATTATCCGCTTCCA
AAACATGTCTAGATGTGTAGTACGTTCGAAAACACCCCAAATGAAAAAAAAAAGTGTCAAAAAACGACACCGGAG
TGATAAGTTATGACATTTTGAATTTCAGTGTGCCCCAATGTTCCCTTTTCAGTACCCTGGAAAAATTTTAGTGGCTAT
ATTAGGGGGGAGGTGTTGGTTGGATCCCTGGGAAAAAAAAAATTTCCCCCCCGAGTTGGTGAAAGGCCAGCAAT
GCCCACACGATGCATCGACCAATGCATGTGAGCGTGTGGGCATGTCCCGATGAGGTCCCTTTTGAGTTTCCAACAC
CGAACAAACCACGTGGGGGCGTGGGAATGGCTCTCGATCACCCTATTGGTACGGGCGATCCTGGGCGACGGCTG
GGCAATGGCTGGGCGTTTGCTGGGCGTTTGCTGGGCATTCGCTGGGCGTTCGCTGGGCGTTCTCTGGGCGATACA
TGGGCCGGTGATTCGGCGTATCACCATAGGAGAATTCCTTACCATCGTCCATGGCACGTCACGAGATGGCATTGG
AAATATGGGGGATGCGTTGGTTGATGGCTTCTTGGACAAGAAAATCCCCCCGAGCTGGTGAAAGGTCGGCAAAG
ACCCACACGTCTCCCTTGTAGCGCCCAATGCATGAGAGCGTGTGGGTATGTCCCGATGAGGCCCCTTCGAGATCAC
AACACCACGTAGGGGCGGAGGGATTGGCTCTCGATCACCCTATTGGCACGGGTGATCGTGGGCGACGGCTGGGC
GATGGCTGGGCGCTCTCTGGGCGTTCTCTGGGCGATACATGGGCCGGTGATTCGGCGTATCACCATGAGATAATT
CCTTGCCATCGTCCATGGCGCGTCACGAGATGGTGTTGGCTGGATGGTTGGGCACATGGGCATAGCTTGGGAGAT
CCCCGGGCAATATGTGGGCATTGGATGGTTGGCGGATATCTTGTGAATGTTTTGAGATCAGCACGTGGAGCGTTG
CTCGGGCTGGTGCTGAGCAACCCCCAAATATGTGAGCTGGGTACTTTTTGTGGACGGTTGTCAATCCCTCATGCTC
CGTAGCTATGAAATGATATGTTCTCCATGCCTCTCCCTCGTATAACTTGCCCCGATACTATCAGGCGACTGTGTTTG

TCTCCCATGCAGCATCTTAGTCCGTATATCGCTCATCCTAATTACATTCGTTTGTAATGCTGCGGCGCAGGTGGCCA
AAGCGGGCGATGGAATAAGGTGTTGCATGGGGGTACTATAGGTCTCCCATGCAACATCTTAGTTCGTATATCGCTC
AATCCGGTTACATTCGTTTGTAACGCTGCGGCGCTGGTGGCCAAAGCGGGCGACGGAATAGGTTGTTGCATGGGG
GTACGCATGGTCTTCCATGCAGCATCTTAGTCCGTATATTGCTCACCCCGGTTACATTTGTTTGTAACGCTGCGGCG
CTGGTGGCCAAAGCGAGCGATGGAAAAAGTTGTTGCATGGTGGATCATGCGTACGTCTGCTCGCATGCACCATCT
TAGGCCTTGATTGCTCAGTCCGGTTACATTCGTTTGTAACGCTGCGGCGCTGGTGGCCAAAGCGGGCGATGGAAT
AAGGTGTTGCATGTGGGAAGGTCGTGGGCTGTTACTTTGATGCTGGGGGTTCACCATGCCTCTCTCCTGGATAACC
TTCCCGTTCCATTCCACGCGGCCATTTGACTTGTTTAAGGCGCTTCATGTGCGAAAAAAACATGGTCATCTAAACGA
GTGGTTTAGGATAGGGGGTGTGATGGGGGAGGAATGTGTTGCTGCCCACCATACATCAGCTTGGCCCGATACTAT
CAGGCGACCGTGTTAGTCTCCTATGCAGCATCTTAGTCCGTATATCGCTCAATCCGGTTACATTCGTTTGTAACGCT
GCGGCGCTGGTGGCCAAAGCGGGCGGTGGAATAGGTTGTTGCATGGGGGTGCGCATGGTCTCCCATGCAACATC
TTAGTCCGTATATTGCTCATCCTGGTTACATTCGTTTGTAACGCTGTGGTGCTGGTGGCCAAAGCGGGCGATGATG
GAATAAGGTGTTGCATGGGGGATCATGCGTACGTCTGCTCCCATGCACCATTTTAGGCCTTGATCGCTCAAATCGG
TTACATTTGTTTGTAACGCTGCGGCGCTGGTGGCCAAAGCGGGCGATGGAATAAGGTGTTGCATGGGGGAAATCA
TGGGCTGTTACTTTGATGCGGGGGGAACATTCAGGATATTGCTACTAGATTGCGTGTTGGGAAGACTACCGTGCC
CAATAACCCAGTTTGCACCCTTCCGACTCCACGTAGTGCAAGCCCAAAATGTCCACGAGATGCCTGATACTGTTCC
GAAGCTTTTCATGTTGGTTTGAAACTTTTGCCCCGATAGTGTGTCCTGACCGTTGCAGCAAGATTTTGCGCACAGCG
AGCGTTTCGAATGGCGCCTCGTTCTACCATATGTATTGCCCTTTGCGGGGTGATGCACACATGGGTGAGCGGGCAT
TGTTCCCAACGCAACGTGTTGGCGTGTGAGTGGTAGTAGAGACATCTCTGCTTTTGGGCTCCGTGCTTCGCGCATC
GAACCATAAGCACATGTTTCCCTCATTGGCGTCCGGCGTACTTGACTGTATGCCGGCAAGTAAAATGGTTCCTGTG
TTGCCTACTCGGAGAAATTGGAAAATATCATTGCTAACGAGATTCATTGCCTTTGGTCGGCCAAAAGCTGGCCGAG
GGCAACATGTTAGTGTGGCTTGTGAAACCTACCGTGGCATTCGCCCGCGTGTCTGGCTAGCAAGCTCGCACGTGCT
TGGGGATATGGAAAACATAGATGGGTCAGGGTTTCAAACTCGGCCGATGCAATTGTAGCTAATTTTGTGACCGCC
GGACCGTAATGATAAGTCCTACTCCCTGCGTGACCAGGTGTAGTTAGGCGCGGCGCGGACAACCGGCGACATAAA
GGAATGCTACCTGGTTGATCCTGCCAGTAGTCATATGCTTGTCTCAAAGATTAAGCCATGCATGTGTAAGTATGAA
CTAATTCAGACTGTGAAACTGCGAATGGCTCATTAAATCAGTTATAGTTTGTTTGATGGTATATGCTACTCGGATAA
CCGTAGTAATTCTAGAGCTAATACGTGCAACAAACCCCGACTTCTGGAAGGGATGCATTTATTAGATAAAAGGTCA
ACGCGGGCTTGTCCCGTTGCTCTGATGATTCATGATAACTCGACGGATCGCACGGCCTTTGTGCCGGCGACGCATC
ATTCAAATTTCTGCCCTATCAACTTTCGATGGTAGGATAGTGGCCTACTATGGTGGTGACGGGTGACGGAGAATTA
GGGTTCGATTCCGGAGAGGGAGCCTGAGAAACGGCTACCACATCCAAGGAAGGCAGCAGGCGCGCAAATTACCC
AATCCTGACACGGGGAGGTAGTGACAATAAATAACAATACCGGGCTCTTAGAGTCTGGTAATTGGAATGAGTACA
ATCTAAATCCCTTAACGAGGATCCATTGGAGGGCAAGTCTGGTGCCAGCAGCCGCGGTAATTCCAGCTCCAATAG
CGTATATTTAAGTTGTTGCAGTTAAAAAGCTCGTAGTTGGACTTTGGGTTTGGTAGGCCGGTCCGCCCTTGTGGTG
TGCACCGGTCGACCAGTCCCTTCTACCGGCGATACGCTCCTGTCCTTAATTGGCCGGGTCGTGCCACCGGTGCTGT
TACTTTGAAGAAATTAGAGTGCTCAAAGCAAGCCCAAGCTCTGGATATATTAGCATGGGATAACATCATAGGATTT
CGGTCCTATTGCGTTGGCCTTCGGGATCGGAGTAATGATTAACAGGGACAGTCGGGGGCATTCGTATTTCATAGTC
AGAGGTGAAATTCTTGGATTTATGAAAGACGAACCACTGCGAAAGCATTTGCCAAGGATGTTTTCATTAATCAAGA
ACGAAAGTTGGGGGCTCGAAGACGATCAGATACCGTCCTAGTCTCAACCATAAACGATGCCGACCAGGGATCGGC
GGATGTTGCTTTTAGGACTCCGCCGGCACCTTATGAGAAATCAAAGTTTTTGGGTTCCGGGGGGAGTATGGTCGC
AAGGCTGAAACTTAAAGGAATTGACGGAAGGGCACCACCAGGAGTGGAGCCTGCGGCTTAATTTGACTCAACAC
GGGGAAACTTACCAGGTCCAGACATAGTAAGGATTGACAGACTGAGAGCTCTTTCTTGATTCTATGGGTGGTGGT
GCATGGCCGTTCTTAGTTGGTGGAGCGATTTGTCTGGTTAATTCCGTTAACGAACGAGACCTCAGCCTGCTAACTA
GCTATGTGGAGGTACCCTCCACGGCCAGCTTCTTAGAGGGACTATGGCCGTTTAGGCCACGGAAGTTTGAGGCAA
TAACAGGTCTGTGATGCCCTTAGATGTTCTGGGCCGCACGCGCGCTACACTGATGTATTCAACGAGTCTATAGCCT

TGGCCGACAGGCCCGGGTAATCTTTGAAAATTTCATCGTGATGGGGATAGATCATTGCAATTGTTGGTCTTCAACG
AGGAATTCCTAGTAAGCGCGAGTCATCAGCTCGCGTTGACTACGTCCCTGCCCTTTGTACACACCGCCCGTCGCTCC
TACCGATTGAATGGTCCGGTGAAATGTTCGGATCGCGGCAACGTGGGTGGTTCGCCGCCGGCGACGCCGCGAGA
AGTCCATTGAACCTTATCATTTAGAGGAAGGAGAAGTCGTAACAAGGTTTCCGTAGGTGAACCTGCGGAAGGATC
ATTGTCGATACCTGCTCAGCAGAACGACCCGCGAACACGTGAAAACAACCTACCATGCCCGGGAGCGGGCTCCGG
CCAGCGACCCGGTGCTGCAACAAAATCCGGCGCAACTGGCGTCAAGGAAAACTCATCGGAAGCAAGGCGTCGGC
TCGTTCGGCGCCGCGTGTCCGAATACTCAAACGACTCTCGGCAACGGATATCTCGGCTCTTGCATCGATGAAGAAC
GTAGCGAAATGCGATACTTGGTGTGAATTGCAGAATCCCGTGAACCATCGAGTCTTTGAACGCAAGTTGCGCCCG
AAGCCTTTTTGGCCGAGGGCACGCCTGCCTGGGCGTCACACACAGCGTCGCCCCCCACCAATCCCTCTGGATGGG
ATGGGGGGCGGAGATTGGCCCCCCGAGCCCTCTGGGGCACGGTCGGCATAAATGTTGGTCCCCGGCGGCGAGCG
TCGCGGTCAGCGGTGGTTGTATACTCATCCCCCGAGGACAAAATGACGCGCACGCCTCGTCGCTCGTCGGGCAGA
GCAAACCCCAGGGAGCACGGCTTCCACCTGCGACCCCAGGTCAGGCGGGATTACCCGCTGAGTTTAAGCATATCA
ATAAGCGGAGGAAAAGAAACTTACGAGGATTCCCCTAGTAACGGCGAGCGAACCGGGAATAGCCCAGCTTGGGA
ATCGGGCGACCCCGTCGTCCGAATTGTAGTCTGTAGAAGCGTCCTCAGCGGCGGACCGGGCCCAAGTCCCCTGGA
AGGTGGCGCCAGAGAGGGTGAGAGCCCCGTCGTGCCCGGACCCTGTCGCACCACGAGGCGCTGTCGGCGAGTCG
GGTTGTTTGGGAATGCAGCCCCAATCGGGCGGTAAATTCCGTCCAAGGCTAAATACTGGCGAGAGACCGATAGCG
AACAAGTACCGCGAGGGAAAGATGAAAAGGACTTTGAAAAGAGAGTCAAAGAGTGCTTGAAATTGTCGGGAGG
GAAGCGGATGGGGGCCGGCGATGTGCGCCGGTCGGATGCGGAACGGCGAGAGCTGGTCCGCCGCTCGGCTCGG
TGCGCGGACCGACGCGGATTGTGGAGGCGACCCAAGCCTGGGCCTCCTCGGAGGCCTGCGGAGATGTCGTCCCC
GCGATTGTGGTGGGCAGCACGCGCCTCACGGCGTGCCTCGGCAACTGCGTGCTCCCGGCGTCGGCCAGTGGGCTC
CCCATTCGGCCCGTCTTGAAACACGGACCAAGGAGTCTGACATGTGTGCGAGTCAGCGGGTGAGTAAACCCGTGA
GGCGCAAGGAAGCTAATTGGCGGGATCCCCCTGTGGGTTGCACCGCCGACCGACCTAGATCTTCTGTGAAGGGTT
CGAGTGTGAGCATACCTGTCGGGACCCGAAAGATGGTGAACTATGCCTGAGCGGGGCGAAGCCAGAGGAAACTC
TGGTGGAGGCCCGCAGCGATACTGACGTGCAAATCGTTCGTCTGACTTGGGTATAGGGGCGAAAGACTAATCGA
ACCATCTAGTAGCTGGTTCCCTCCGAAGTTTCCCTCAGGATAGCTGGAGCTCGCGGGCGAGTTCTATCAGGTAAAG
CCAATGATTAGAGGCATCGGGGGCGCAACGCCCTCGACCTATTCTCAAACTTTAAATAGGTAGGACGGCGCGGCT
ACTTCGCTGAGCCGCGCCACGGAATCGAGTGCTCCAAGTGGGCCATTTTTGGTAAGCAGAACTGGCGATGCGGGA
TGAACCGGAAGCCGGGTTACGGTGCCCAACTGCGCGCTAACCCAGAACCCACAAAGGGTGTTGGTCGATTAAGAC
AGCAGGACGGTGGTCATGGAAGTCGAAATCCGCTAAGGAGTGTGTAACAACTCACCTGCCGAATCAACTAGCCCC
GAAAATGGATGGCGCTGAAGCGCGCGACCCACACCCGGCCGTCAGGGCAATTGTTAGGCCCTGATGAGTAGGAG
GGCGCAGCGGTGGCCGCGAAACCCAGGGCGCGAGCCCGGGTGGAGCCGCCGTTGGTGCAGATCTTGGTGGTAG
TAGCAAATATTCAAATGAGAACTTTGAAGGCCGAAGAGGGGAAAGGTTCCATGTGAACGGCACTTGCACATGGGT
TAGTCGATCCTAAGAGACGGCCGAAAGGCGTCAGAGAGCGTGCATCACGCGAGCTTCGAAAGGGAATCGGGTTA
AAATTCCTGAACCGGGACACAGTGGTCGACGGCAACGCTAGGAAGTCCGGAGACGTCGGCGGGGGCCTCGGGA
AGAGTTATCTTTTCTGTTTAACAGCCTGCCCACCCTGGAAACGGCTCAGCCGGAGGTAGGGTCCAGCGGCTGGAA
GAGCACCGCACGTCGCGTGGTGTCCGGTGCGCCCCCGGCGGCCCTTGAAAATCCGGAGGACCGAATGCCAACTGT
GCCCGGTCGTACTCATAACCGCATCAGGTCTCCAAGGTGAACAGCCTCTGGTCGATGGAACAATGTAGGCAAGGG
AAGTCGGCAAAATGGATCCGTAACCTCGGGAAAAGGATTGGCTCTGAGGGCTGGGCACGGGGGTCCCAGTCCCG
AACCCGTTGGCTGCCGGCGGACTGCTCGAGCTGCTTCCGTGGCGAGAGCGGGTCGCCGCGTGCCGGCCGGGGGA
TGGACTGGGAACGGCTCCTTCGGGGGCCTTCCCCGGGCGTCGAACAGCCAACTCAGAACTGGTACGGACAAGGG
GAATCCGACTGTTTAATTAAAACAAAGCATTGCGATGGTCCCTGCGGATGCTAACGCAATGTGATTTCTGCCCAGT
GCTCTGAATGTCAAAGTGAAGAAATTCAACCAAGCGCGGGTAAACGGCGGGAGTAACTATGACTCTCTTAAGGTA
GCCAAATGCCTCGTCATCTAATTAGTGACGCGCATGAATGGATTAACGAGATTCCCACTGTCCCTGTCTACTATCCA
GCGAAACCACAGCCAAGGGAACGGGCTTGGCAGAATCAGCGGGGAAAGAAGACCCTGTTGAGCTTGACTCTAGT

CCGACTTTGTGAAATGACTTGAGAGGTGTAGGATAAGTGGGAGCCGACTCGTGCGGCGAAAGTGAAATACCACT
ACTTTTAACGTTATTTTACTTATTCCGTGAATCGGAAGCGGGGCACTGCTCCTCTTTTTGGATCCAAGTTCGGTCTC
GACGGGACAATCCGGGCGGAAGACATTGTCAGGTGGGGAGTTTGGCTGGGGCGGCACATCTGTTAAAAGATAAC
GCAGGTGTCCTAAGATGAGCTCAACGAGAACAGAAATCTCGTGTGGAACAAAAGGGTAAAAGCTCGTTTGATTCT
GATTTCCAGTACGAATACGAACCGTGAAAGCGTGGCCTATCGATCCTTTAGACCTTCGGAATTTGAAGCTAGAGGT
GTCAGAAAAGTTACCACAGGGATAACTGGCTTGTGGCAGCCAAGCGTTCATAGCGACGTTGCTTTTTGATCCTTCG
ATGTCGGCTCTTCCTATCATTGTGAAGCAGAATTCACCAAGTGTTGGATTGTTCACCCACCAATAGGGAACGTGAG
CTGGGTTTAGACCGTCGTGAGACAGGTTAGTTTTACCCTACTGATGATAGTGTCGCGATAGTAATTCAACCTAGTA
CGAGAGGAACCGTTGATTCACACAATTGGTCATCGCGCTTGGTTGAAAAGCCAGTGGCGCGAAGCTACCGTGTGC
AGGATTATGACTGAACGCCTCTAAGTCAGAATCCAGGCTAGAGAAGCGACGCATGCGCCCGTCGCCCGATTGCCG
ACCTGCAGTAGGGGTCCTTGGACCCCCAAAGGCACGTGTCATTGGCGTAGCTCCCGTGGCCGATGAGTCGCGTGG
TGCCGCCTTGAAGTATAATTCCTACCGAGCGGCGGGCTGAATCCTTTGCAGACGACTTAAATACGCGACGGGGTA
TTGTAAGTGGCAGAGTGGCCTTGCTGCCACGATCCACTGAGATTCAGCCCCATGTCGCATCGATTCGTCCCTCCCC
ATCCAAAAAA

>A_sylvestris_1/1-12084

ACTACAACACACCAATTGCCTCGTATACCACTCGAGGTTACAACTATTTTACGAGAGAGTGATACTTGGAAAAAAT
TCTAAGTCTGGGAAATAAATACTCCACTTCATATGTAAGTGCCATCGCCCGGACAAACCATGGTCGAGTATCATCG
CCAGTAGGGCTTGTACGAGCGCCCGTAGCGTATCACTATGTATAGTGAACATGGCTTGCACTAGCTCCCGTAGCGT
ATTGCCATGTATCACCCGCATGGCTTGCACTAGCCCCTGTAACGTATCGCCATGTATCGCCCGTAGCGTATCGCCAT
GTATCGCCCGCATGGCTTGCACTAGCCTGTGGGCAAATATGCATCGACTTCCCCGACGATGTTTGTGAGATGGATC
AACCGAGGAAATATGTCACGGGATAGCATATCCCCAAATAAATATGCACAATGGCATGAATCACCTATGCAAATA
ACCCCTGTCATGATGATGCTTGTGAAATGGGCAACTATTGGGCATTTCATAGGCATCGTTTGGGCATGAACTGGGC
ATTTGCTGGCCACCATGTATCGCCCGCATGCCTTGTACTAGCGCCCGTAGCGTATCGCCATGTATCACCCGCATGGC
TTGCACTAGCTCCCGTCGCGTATCGCCATGTATCGCCCGTAGTGTATCGCCATGTATCACCCGCATGGCTTGCACCA
GCTCCCGTAGCGTATCGCCATGTATCGACCGTAGTGTATCGCCATGTATCACCCGCATGGCTTGCACCAGCTCCCGT
AGCGTATCGCCATGTATCGCCCGTAGCGTATCGCCATGTATTGCCCGCATAGCTTGGGCATTTCCTAGGCATCTTTT
GGGCGTGTGCTGGGCATTTGATAGCCGTCATGTATCGCCCGCATGGCTTGCACTAGCTCCCGTAGCGTATCGCCAT
GTATCGCCCGTAGCGTATCGCCATGTATCACCCGCATGGCTTGCACCAGCTCCCGTAGCGTATCGCCATGTATCGC
CCGCATGGCTTACACTAGCGCCCGTAGCGTATCGCCATGTATTGCCCGCATGGCTTGGGCATTTCATAGGCATCTG
TTGGGCGTGAGCTGGGCATTTGATGGGCACCATGTATCGCCCGCAAGGCTTGCACCAGTGCCCGTAGCGTATCGC
CATGTATCGCCCGTAGCGTATCGCCATGTATCACCCGCATGGCTTGCACCAGCTCCCGTAGCGTATCGCCATGTATC
GCCCGTAGCGTATCGCCATGTATTGCCCGCATAGCTTGGGCATTTCCTAGGCATCTTTTGGGCGTGTGCTGGGCAT
TTGATAGCCGTCATGTATCGCCCGCATGGCTTGTACCAACCCCCGTAGCGTATCGCCATGTATCGCCCGTAGCGTAT
CGCCATGTATCACCCGCATGGCTTGCACCAACCCCCGTAGCGTATCGCCATGTATCGCCCGTAGCGTATCGCCATG
GCTTGCATTAGCGCCCGTAGCGTATCGCCATGGCTTGCATTAGCGCCCGTTGCGTATCGCCATGTATCGCCCGTAG
CGTATCGCCATGTATCACCCGCATGGCTTGCACCAGCTCCCGTAGCGTATCGCCATGTATCGCCCGCATGGCTTACA
CTAGCGCCCGTAGCGTATCGCCATGTATTGCCCGCATGGCTTGGGCATTTCATAGGCATCTGTTGGGCGTGAGCTG
GGCATTTGATGGGCACCATGTATCGCCCGCAAGGCTTGCACCAGTGCCCGTAGCGTATCGCCATGTATCGCCCGTA
GCGTATCGCCATGTATCACCCGCATGGCTTGCACCAGCTCCCGTAGCGTATCGCCATGAATCGCCCGTAGCGTATC
GCCATGTATTGCCCGCATAGCTTGGGCATTTCCTAGGCATCATTTGGGCGTGAGCTGGGCATTTGATGGCCGTCAT
GTATCGCCCGCATGGCTTGCACCAGCCCCCTAGCGTATCGCCATGTATCGCCCGTAGCGTATCGCCATGTATCACCC
GCATGCTTGCACCAGCTCCCGTAGCGTATCGCCATGTATCGCCCACATGGCTTGCACTAGCTCCCGTAGCGTATCG

CCATGTATCGCCCGTAGCGTATCGCCATGTATCACCCGCATGGCTTGCACCAGCTCCCGTAGCGTATCGCCATGTAT
CGCCCGCATGGCTTACACCAGCGCCCGTAGCGTATCGCCATGTATCGCCCGCATGGCTTGCACCAGCGCCCGTAGC
GTATCGCCATGTATCGCCCGTAGCGTATCGCCATGTATCACCCGCATGGCTTGCACCACCTCCCGTAGCGTATCGCC
ATGTATCGCACACATGGCTTGCACTAGCGCCCGTAGCGTATCGCCATGTATTGCCCGCATGGCTTGGGCATTTCAT
AGGCATCTCTTGGGCGTGAGCTGGGCATTTGATGGCCGCCATGTATCGCCCGCATGGTTGCACCCGCGCCCGTAG
CGTATCGCCATGTATCGCCCGTAGCGTATCGCCATGTATCACCCGCATGGCTTGCACCAGCTCCCGTAGCGTATCG
CCATGTATCGCACGCATGGCTTGCACTAGCGCCCGTAGCGTATCGCCATGTATTGCCCGCATGGCTTGGGCATTTC
ATAGGCATCTTTTGGGCGTGAGTTGGGCATTTGATGGCCGCCATGTATCGCCCGCATGGCTTGCACCAGCGCCCGT
AGCGTATCACCATGTATCGCCCGCATGGCTTGCACTAGCGCCCGTAGCGTATCACCATGTATCGCCCGTAGCGTAT
CGCCATGTATCGACCGCATGGCTTGCACTAGCGCCCATAGCGTATCGCCATGTATCGCCCGTAGCGTATCGCCATG
TATCACCCGCATGGCTTGCACCAGCTCCCGTAGCGTATCGCCATGTATCGCCCGCATGGCTTGCACTAGCGCCCGT
AGCGTATCGCCATGTATCGCCCGCATGGCTTGGGCATTTGCTGGGCATCCTTGGGCATTGGCTAGGCGTTGCTTGG
GCATTGGCTGGGCGCTATGCATCCCCGGGCAAGATCACTCGGGGAATGAGACCTATGCATCGTCTGGTCAAGTAT
GGTTATGGTTCTCTCGGGGAACCTCAACCATGCATCACCACATAAGAGATATTGAGGTTGCATGGGTGATTCTATT
GAGGCTGCGTGGGCGATGCATGGCTAGGCCGACGTGCATCCGGGAGGAAAATTCTTGAAAACACATCTAAATGG
TCCAATTTTTTTGTGACAACTTAGTAGTGGCTATTACTCATAAAAACACACCTAAGGCAAAAAGATTGAGAAAAAA
AGAGGTCCGGGGAAAAAAAATCGTAAAAAATCATTTTCGTCAATTCTGAGTATGAATACTCCATTATCCGCTTCCA
AAACATGTCTAGATGTGTAGTACGTTCGAAAACACCCCAAATGAAAAAAAAAAGTGTCAAAAAACGACACCGGAG
TGATAAGTTATGACATTTTGAATTTCAGTGTGCCCCAATGTTCCCTTTTCAGTACCCTGGAAAAATTTTAGTGGCTAT
ATTAGGGGGGAGGTGTTGGTTGGATCCCTGGGAAAAAAAAAATTTCCCCCCCGAGTTGGTGAAAGGCCAGCAAT
GCCCACACGATGCATCGACCAATGCATGTGAGCGTGTGGGCATGTCCCGATGAGGTCCCTTTTGAGTTTCCAACAC
CGAACAAACCACGTGGGGGCGTGGGAATGGCTCTCGATCACCCTATTGGTACGGGCGATCCTGGGCGACGGCTG
GGCAATGGCTGGGCGTTTGCTGGGCGTTTGCTGGGCATTCGCTGGGCGTTCGCTGGGCGTTCTCTGGGCGATACA
TGGGCCGGTGATTCGGCGTATCACCATAGGAGAATTCCTTACCATCGTCCATGGCACGTCACGAGATGGCATTGG
AAATATGGGGGATGCGTTGGTTGATGGCTTCTTGGACAAGAAAATCCCCCCGAGCTGGTGAAAGGTCGGCAAAG
ACCCACACGTCTCCCTTGTAGCGCCCAATGCATGAGAGCGTGTGGGTATGTCCCGATGAGGCCCCTTCGAGATCAC
AACACCACGTAGGGGCGGAGGGATTGGCTCTCGATCACCCTATTGGCACGGGTGATCGTGGGCGACGGCTGGGC
GATGGCTGGGCGCTCTCTGGGCGTTCTCTGGGCGATACATGGGCCGGTGATTCGGCGTATCACCATGAGATAATT
CCTTGCCATCGTCCATGGCGCGTCACGAGATGGTGTTGGCTGGATGGTTGGGCACATGGGCATAGCTTGGGAGAT
CCCCGGGCAATATGTGGGCATTGGATGGTTGGCGGATATCTTGTGAATGTTTTGAGATCAGCACGTGGAGCGTTG
CTCGGGCTGGTGCTGAGCAACCCCCAAATATGTGAGCTGGGTACTTTTTGTGGACGGTTGTCAATCCTCATGCTTG
GTAGCTATGAAATGAAATGTTCTCCATGCCTCTCCCTCGTATAACTTGCCCCGATACTATCAGGCGACTGTGTTTGT
CTCCCATGCAGCATCTTAGTCCGTATATCGCTCATCCTAATTACATTCGTTTGTAATGCTGCGGCGCAGGTGGCCAA
AGCGGGCGATGGAATAAGGTGTTGCATGGGGGTACTATAGGTCTCCCATGCAACATCTTAGTTCGTATATCGCTCA
ATCCGGTTACATTCGTTTGTAACGCTGCGGCGCTGGTGGCCAAAGCGGGCGACGGAATAGGTTGTTGCATGGGG
GTACGCATGGTCTTCCATGCAGCATCTTAGTCCGTATATTGCTCACCCCGGTTACATTTGTTTGTAACGCTGCGGCG
CTGGTGGCCAAAGCGAGCGATGGAAAAAGTTGTTGCATGGTGGATCATGCGTACGTCTGCTCGCATGCACCATCT
TAGGCCTTGATTGCTCAGTCCGGTTACATTCGTTTGTAACGCTGCGGCGCTGGTGGCCAAAGCGGGCGATGGAAT
AAGGTGTTGCATGTGGGAAGGTCGTGGGCTGTTACTTTGATGCTGGGGGTTCACCATGCCTCTCTCCTGGATAACC
TTCCCGTTCCATTCCACGCGGCCATTTGACTTGTTTAAGGCGCTTCATGTGCGAAAAAAACATGGTCATCTAAACGA
GTGGTTTAGGATAGGGGGTGTGATGGGGGAGGAGTGTGTTGCTGCCCACCATGCATCAGCTTGGCCCGATACTAT
CAGGCGACCGTGTTAGTCTCCTATGCAGCATCTTAGTCCGTATATCGCTCAATCCGGTTACATTCGTTTGTAACGCT
GCGGCGCTGGTGGCCAAAGCGGGCGGTGGAATAGGTTGTTGCATGGGGGTGCGCATGGTCTCCCATGCAACATC
TTAGTCCGTATATTGCTCATCCTGGTTACATTCGTTTGTAACGCTGTGGTGCTGGTGGCCAAAGCGGGCGATGATG

GAATAAGGTGTTGCATGGGGGATCATGCGTACGTCTGCTCCCATGCACCATTTTAGGCCTTGATTGCTCAAATCGG
TTACATTTGTTTGTAACGCTGCGGCGCTGGTGGCCAAAGCGGGCGATGGAATAAGGTGTTGCATGGGGGAAATCA
TGGGCTGTTACTTTGATGCGGGGGGAACATTCAGGATATTGCTACTAGATTGCGTGTTGGGAAGACTACCGTGCC
CAATAACCCAGTTTGCACCCTTCCGACTCCACGTAGTGCAAGCACAAAATGTCCACGAGATGCCTGATACTGTTCC
GAAGCTTTTCATGTTGGTTTGAAACTTTTGCCCCGATAGTGTGTCCTGACCGTTGCAGCAAGATTTTGCGCACAGCG
AGCGTTTCGAATGGCGCCTCGTTCTACCATATGTATTGCCCTTTGCGGGGTGATGCACACATGGGTGAGCGGGCAT
TGTTCCCAACGCAACGTGTTGGCGTGTGAGTGGTAGTAGAGACATCTCTGCTTTTGGGCTCCGTGCTTCGCGCATC
GAACCATAAGCACATGTTTCCCTCATTGGCGTCCGGCGTACTTGACTGTATGCCGGCAAGTAAAATGGTTCCTGTG
TTGCCTACTCGGAGAAATTGGAAAATATCATTGCTAACGAGATTCATTGCCTTTGGTCGGCCAAAAGCTGGCCGAG
GGCAACATGTTAGTGTGGCTTGTGAAACCTACCGTGGCATTCGCCCGCGTGTCTGGCTAGCAAGCTCGCACGTGCT
TGGGGATATGGAAAACATAGATGGGTCAGGGTTTCAAACTCGGCCGATGCAATTGTAGCTAATTTTGTGACCGCC
GGACCGTAATGATAAGTCCTACTCCCTGCGTGACCAGGTGTAGTTAGGCGCGGCGCGGACAACCGGCGACATAAA
GGAATGCTACCTGGTTGATCCTGCCAGTAGTCATATGCTTGTCTCAAAGATTAAGCCATGCATGTGTAAGTATGAA
CTAATTCAGACTGTGAAACTGCGAATGGCTCATTAAATCAGTTATAGTTTGTTTGATGGTATATGCTACTCGGATAA
CCGTAGTAATTCTAGAGCTAATACGTGCAACAAACCCCGACTTCTGGAAGGGATGCATTTATTAGATAAAAGGTCA
ACGCGGGCTTGTCCCGTTGCTCTGATGATTCATGATAACTCGACGGATCGCACGGCCTTTGTGCCGGCGACGCATC
ATTCAAATTTCTGCCCTATCAACTTTCGATGGTAGGATAGTGGCCTACTATGGTGGTGACGGGTGACGGAGAATTA
GGGTTCGATTCCGGAGAGGGAGCCTGAGAAACGGCTACCACATCCAAGGAAGGCAGCAGGCGCGCAAATTACCC
AATCCTGACACGGGGAGGTAGTGACAATAAATAACAATACCGGGCTCTTAGAGTCTGGTAATTGGAATGAGTACA
ATCTAAATCCCTTAACGAGGATCCATTGGAGGGCAAGTCTGGTGCCAGCAGCCGCGGTAATTCCAGCTCCAATAG
CGTATATTTAAGTTGTTGCAGTTAAAAAGCTCGTAGTTGGACTTTGGGTTTGGTAGGCCGGTCCGCCCTTGTGGTG
TGCACCGGTCGACCAGTCCCTTCTACCGGCGATACGCTCCTGTCCTTAATTGGCCGGGTCGTGCCACCGGTGCTGT
TACTTTGAAGAAATTAGAGTGCTCAAAGCAAGCCCAAGCTCTGGATATATTAGCATGGGATAACATCATAGGATTT
CGGTCCTATTGCGTTGGCCTTCGGGATCGGAGTAATGATTAACAGGGACAGTCGGGGGCATTCGTATTTCATAGTC
AGAGGTGAAATTCTTGGATTTATGAAAGACGAACCACTGCGAAAGCATTTGCCAAGGATGTTTTCATTAATCAAGA
ACGAAAGTTGGGGGCTCGAAGACGATCAGATACCGTCCTAGTCTCAACCATAAACGATGCCGACCAGGGATCGGC
GGATGTTGCTTTTAGGACTCCGCCGGCACCTTATGAGAAATCAAAGTTTTTGGGTTCCGGGGGGAGTATGGTCGC
AAGGCTGAAACTTAAAGGAATTGACGGAAGGGCACCACCAGGAGTGGAGCCTGCGGCTTAATTTGACTCAACAC
GGGGAAACTTACCAGGTCCAGACATAGTAAGGATTGACAGACTGAGAGCTCTTTCTTGATTCTATGGGTGGTGGT
GCATGGCCGTTCTTAGTTGGTGGAGCGATTTGTCTGGTTAATTCCGTTAACGAACGAGACCTCAGCCTGCTAACTA
GCTATGTGGAGGTACCCTCCACGGCCAGCTTCTTAGAGGGACTATGGCCGTTTAGGCCACGGAAGTTTGAGGCAA
TAACAGGTCTGTGATGCCCTTAGATGTTCTGGGCCGCACGCGCGCTACACTGATGTATTCAACGAGTCTATAGCCT
TGGCCGACAGGCCCGGGTAATCTTTGAAAATTTCATCGTGATGGGGATAGATCATTGCAATTGTTGGTCTTCAACG
AGGAATTCCTAGTAAGCGCGAGTCATCAGCTCGCGTTGACTACGTCCCTGCCCTTTGTACACACCGCCCGTCGCTCC
TACCGATTGAATGGTCCGGTGAAATGTTCGGATCGCGGCAACGTGGGTGGTTCGCCGCCGGCGACGCCGCGAGA
AGTCCATTGAACCTTATCATTTAGAGGAAGGAGAAGTCGTAACAAGGTTTCCGTAGGTGAACCTGCGGAAGGATC
ATTGTCGATACCTGCTCAGCAGAACGACCCGCGAACACGTGAAAACAACCTACCATGCCCGGGAGCGGGCTCCGG
CCAGCGACCCGGTGCTGCAACAAAATCCGGCGCAACTGGCGTCAAGGAAAACTCACCGGAAGCAAGGCGTCGGC
TCGTTCGGCGCCGCGTGTCCGAATACTCAAACGACTCTCGGCAACGGATATCTCGGCTCTTGCATCGATGAAGAAC
GTAGCGAAATGCGATACTTGGTGTGAATTGCAGAATCCCGTGAACCATCGAGTCTTTGAACGCAAGTTGCGCCCG
AAGCCTTTTTGGCCGAGGGCACGCCTGCCTGGGCGTCACACACAGCGTCGCCCCCCACCAATCCCTCTGGATGGG
ATGGGGGGCGGAGATTGGCCCCCCGAGCCCTCCGGGGCACGGTCGGCATAAATGTTGGTCCCCGGCGGCGAGCG
TCGCGGTCAGCGGTGGTTGTATACTCATCCCCCGAGGACAAAATGACGCGCACGCCTCGTCGCTCGTCGGGCAGA
GCAAACCCCAGGGAGCACGGCTTCCACCTGCGACCCCAGGTCAGGCGGGATTACCCGCTGAGTTTAAGCATATCA

ATAAGCGGAGGAAAAGAAACTTACGAGGATTCCCCTAGTAACGGCGAGCGAACCGGGAATAGCCCAGCTTGGGA
ATCGGGCGACCCCGTCGTCCGAATTGTAGTCTGTAGAAGCGTCCTCAGCGGCGGACCGGGCCCAAGTCCCCTGGA
AGGTGGCGCCAGAGAGGGTGAGAGCCCCGTCGTGCCCGGACCCTGTCGCACCACGAGGCGCTGTCGGCGAGTCG
GGTTGTTTGGGAATGCAGCCCCAATCGGGCGGTAAATTCCGTCCAAGGCTAAATACTGGCGAGAGACCGATAGCG
AACAAGTACCGCGAGGGAAAGATGAAAAGGACTTTGAAAAGAGAGTCAAAGAGTGCTTGAAATTGTCGGGAGG
GAAGCGGATGGGGGCCGGCGATGTGCGCCGGTCGGATGCGGAACGGCGAGAGCTGGTCCGCCGCTCGGCTCGG
TGCGCGGACCGACGCGGATTGTGGAGGCGACCCAAGCCTGGGCCTCCTCGGAGGCCTGCGGAGATGTCGTCCCC
GCGATTGTGGTGGGCAGCACGCGCCTCACGGCGTGCCTCGGCAACTGCGTGCTCCCGGCGTCGGCCAGTGGGCTC
CCCATTCGGCCCGTCTTGAAACACGGACCAAGGAGTCTGACATGTGTGCGAGTCAGCGGGTGAGTAAACCCGTGA
GGCGCAAGGAAGCTAATTGGCGGGATCCCCCTGTGGGTTGCACCGCCGACCGACCTAGATCTTCTGTGAAGGGTT
CGAGTGTGAGCATACCTGTCGGGACCCGAAAGATGGTGAACTATGCCTGAGCGGGGCGAAGCCAGAGGAAACTC
TGGTGGAGGCCCGCAGCGATACTGACGTGCAAATCGTTCGTCTGACTTGGGTATAGGGGCGAAAGACTAATCGA
ACCATCTAGTAGCTGGTTCCCTCCGAAGTTTCCCTCAGGATAGCTGGAGCTCGCGGGCGAGTTCTATCAGGTAAAG
CCAATGATTAGAGGCATCGGGGGCGCAACGCCCTCGACCTATTCTCAAACTTTAAATAGGTAGGACGGCGCGGCT
ACTTCGCTGAGCCGCGCCACGGAATCGAGTGCTCCAAGTGGGCCATTTTTGGTAAGCAGAACTGGCGATGCGGGA
TGAACCGGAAGCCGGGTTACGGTGCCCAACTGCGCGCTAACCCAGAACCCACAAAGGGTGTTGGTCGATTAAGAC
AGCAGGACGGTGGTCATGGAAGTCGAAATCCGCTAAGGAGTGTGTAACAACTCACCTGCCGAATCAACTAGCCCC
GAAAATGGATGGCGCTGAAGCGCGCGACCCACACCCGGCCGTCAGGGCAATTGTTAGGCCCTGATGAGTAGGAG
GGCGCAGCGGTGGCCGCGAAACCCAGGGCGCGAGCCCGGGTGGAGCCGCCGTTGGTGCAGATCTTGGTGGTAG
TAGCAAATATTCAAATGAGAACTTTGAAGGCCGAAGAGGGGAAAGGTTCCATGTGAACGGCACTTGCACATGGGT
TAGTCGATCCTAAGAGACGGCCGAAAGGCGTCAGAGAGCGTGCATCACGCGAGCTTCGAAAGGGAATCGGGTTA
AAATTCCTGAACCGGGACACAGTGGTCGACGGCAACGCTAGGAAGTCCGGAGACGTCGGCGGGGGCCTCGGGA
AGAGTTATCTTTTCTGTTTAACAGCCTGCCCACCCTGGAAACGGCTCAGCCGGAGGTAGGGTCCAGCGGCTGGAA
GAGCACCGCACGTCGCGTGGTGTCCGGTGCGCCCCCGGCGGCCCTTGAAAATCCGGAGGACCGAATGCCAACTGT
GCCCGGTCGTACTCATAACCGCATCAGGTCTCCAAGGTGAACAGCCTCTGGTCGATGGAACAATGTAGGCAAGGG
AAGTCGGCAAAATGGATCCGTAACCTCGGGAAAAGGATTGGCTCTGAGGGCTGGGCACGGGGGTCCCAGTCCCG
AACCCGTTGGCTGCCGGCGGACTGCTCGAGCTGCTTCCGTGGCGAGAGCGGGTCGCCGCGTGCCGGCCGGGGGA
TGGACTGGGAACGGCTCCTTCGGGGGCCTTCCCCGGGCGTCGAACAGCCAACTCAGAACTGGTACGGACAAGGG
GAATCCGACTGTTTAATTAAAACAAAGCATTGCGATGGTCCCTGCGGATGCTAACGCAATGTGATTTCTGCCCAGT
GCTCTGAATGTCAAAGTGAAGAAATTCAACCAAGCGCGGGTAAACGGCGGGAGTAACTATGACTCTCTTAAGGTA
GCCAAATGCCTCGTCATCTAATTAGTGACGCGCATGAATGGATTAACGAGATTCCCACTGTCCCTGTCTACTATCCA
GCGAAACCACAGCCAAGGGAACGGGCTTGGCAGAATCAGCGGGGAAAGAAGACCCTGTTGAGCTTGACTCTAGT
CCGACTTTGTGAAATGACTTGAGAGGTGTAGGATAAGTGGGAGCCGACTCGTGCGGCGAAAGTGAAATACCACT
ACTTTTAACGTTATTTTACTTATTCCGTGAATCGGAAGCGGGGCACTGCTCCTCTTTTTGGATCCAAGTTCGGTCTC
GACGGGACAATCCGGGCGGAAGACATTGTCAGGTGGGGAGTTTGGCTGGGGCGGCACATCTGTTAAAAGATAAC
GCAGGTGTCCTAAGATGAGCTCAACGAGAACAGAAATCTCGTGTGGAACAAAAGGGTAAAAGCTCGTTTGATTCT
GATTTCCAGTACGAATACGAACCGTGAAAGCGTGGCCTATCGATCCTTTAGACCTTCGGAATTTGAAGCTAGAGGT
GTCAGAAAAGTTACCACAGGGATAACTGGCTTGTGGCAGCCAAGCGTTCATAGCGACGTTGCTTTTTGATCCTTCG
ATGTCGGCTCTTCCTATCATTGTGAAGCAGAATTCACCAAGTGTTGGATTGTTCACCCACCAATAGGGAACGTGAG
CTGGGTTTAGACCGTCGTGAGACAGGTTAGTTTTACCCTACTGATGATAGTGTCGCGATAGTAATTCAACCTAGTA
CGAGAGGAACCGTTGATTCACACAATTGGTCATCGCGCTTGGTTGAAAAGCCAGTGGCGCGAAGCTACCGTGTGC
AGGATTATGACTGAACGCCTCTAAGTCAGAATCCAGGCTAGAGAAGCGACGCATGCGCCCGTCGCCCGATTGCCG
ACCTGCAGTAGGGGTCCTTGGACCCCCAAAGGCACGTGTCACTGGCGTAGCTCCCGTGGCCGATGAGTCGCGTGG
TGCCGCCTTGAAGTATAATTCCTACCGAGCGGCGGGCTGAATCCTTTGCAGACGACTTAAATACGCGACGGGGTA

TTGTAAGTGGCAGAGTGGCCTTGCTGCCACGATCCACTGAGATTCAGCCCCATGTCGCATCGATTCGTCCCTCCCC
ATCCAAAAAAA

>A_sylvestris_3/1-12084

ACTACAACACACCAATTGCCTCGTATACCACTCGAGGTTACAACTATTTTACGAGAGAGTGATACTTGGAAAAAAT
TCTAAGTCTGGGAAATAAATACTCCACTTCATATGTAAGTGCCATCGCCCGGACAAACCATGGTCGAGTATCATCG
CCAGTAGGGCTTGTACGAGCGCCCGTAGCGTATCACTATGTATAGTGAACATGGCTTGCACTAGCTCCCGTAGCGT
ATTGCCATGTATCACCCGCATGGCTTGCACTAGCCCCTGTAACGTATCGCCATGTATCGCCCGTAGCGTATCGCCAT
GTATCGCCCGCATGGCTTGCACTAGCCTGTGGGCAAATATGCATCGACTTCCCCGACGATGTTTGTGAGATGGATC
AACCGAGGAAATATGTCACGGGATAGCATATCCCCAAATAAATATGCACAATGGCATGAATCACCTATGCAAATA
ACCCCTGTCATGATGATGCTTGTGAAATGGGCAACTATTGGGCATTTCATAGGCATCGTTTGGGCATGAACTGGGC
ATTTGCTGGCCACCATGTATCGCCCGCATGCCTTGTACTAGCGCCCGTAGCGTATCGCCATGTATCACCCGCATGGC
TTGCACTAGCTCCCGTCGCGTATCGCCATGTATCGCCCGTAGTGTATCGCCATGTATCACCCGCATGGCTTGCACCA
GCTCCCGTAGCGTATCGCCATGTATCGACCGTAGTGTATCGCCATGTATCACCCGCATGGCTTGCACCAGCTCCCGT
AGCGTATCGCCATGTATCGCCCGTAGCGTATCGCCATGTATTGCCCGCATAGCTTGGGCATTTCCTAGGCATCTTTT
GGGCGTGTGCTGGGCATTTGATAGCCGTCATGTATCGCCCGCATGGCTTGTACCAACCCCCGTAGCGTATCGCCAT
GTATCGCCCGTAGCGTATCGCCATGTATCACCCGCATGGCTTGCACCAACCCCCGTAGCGTATCGCCATGTATCGC
CCGTAGCGTATCGCCATGGCTTGCATTAGCGCCCGTAGCGTATCGCCATGGCTTGCATTAGCGCCCGTTGCGTATC
GCCATGTATCGCCCGTAGCGTATCGCCATGTATCACCCGCATGGCTTGCACCAGCTCCCGTAGCGTATCGCCATGT
ATCGCCCGCATGGCTTACACTAGCGCCCGTAGCGTATCGCCATGTATTGCCCGCATGGCTTGGGCATTTCATAGGC
ATCTGTTGGGCGTGAGCTGGGCATTTGATGGGCACCATGTATCGCCCGCAAGGCTTGCACCAGTGCCCGTAGCGT
ATCGCCATGTATCGCCCGTAGCGTATCGCCATGTATCACCCGCATGGCTTGCACCAGCTCCCGTAGCGTATCGCCAT
GTATCGCCCGTAGCGTATCGCCATGTATTGCCCGCATAGCTTGGGCATTTCCTAGGCATCTTTTGGGCGTGTGCTG
GGCATTTGATAGCCGTCATGTATCGCCCGCATGGCTTGCACTAGCTCCCGTAGCGTATCGCCATGTATCGCCCGTA
GCGTATCGCCATGTATCACCCGCATGGCTTGCACCAGCTCCCGTAGCGTATCGCCATGTATCGCCCGCATGGCTTA
CACTAGCGCCCGTAGCGTATCGCCATGTATTGCCCGCATGGCTTGGGCATTTCATAGGCATCTGTTGGGCGTGAGC
TGGGCATTTGATGGGCACCATGTATCGCCCGCAAGGCTTGCACCAGTGCCCGTAGCGTATCGCCATGTATCGCCCG
TAGCGTATCGCCATGTATCACCCGCATGGCTTGCACCAGCTCCCGTAGCGTATCGCCATGAATCGCCCGTAGCGTA
TCGCCATGTATTGCCCGCATAGCTTGGGCATTTCCTAGGCATCATTTGGGCGTGAGCTGGGCATTTGATGGCCGTC
ATGTATCGCCCGCATGGCTTGCACCAGCCCCCTAGCGTATCGCCATGTATCGCCCGTAGCGTATCGCCATGTATCAC
CCGCATGCTTGCACCAGCTCCCGTAGCGTATCGCCATGTATCGCCCACATGGCTTGCACTAGCTCCCGTAGCGTATC
GCCATGTATCGCCCGTAGCGTATCGCCATGTATCACCCGCATGGCTTGCACCAGCTCCCGTAGCGTATCGCCATGT
ATCGCCCGCATGGCTTACACCAGCGCCCGTAGCGTATCGCCATGTATCGCCCGCATGGCTTGCACCAGCGCCCGTA
GCGTATCGCCATGTATCGCCCGTAGCGTATCGCCATGTATCACCCGCATGGCTTGCACCACCTCCCGTAGCGTATC
GCCATGTATCGCACACATGGCTTGCACTAGCGCCCGTAGCGTATCGCCATGTATTGCCCGCATGGCTTGGGCATTT
CATAGGCATCTCTTGGGCGTGAGCTGGGCATTTGATGGCCGCCATGTATCGCCCGCATGGTTGCACCCGCGCCCGT
AGCGTATCGCCATGTATCGCCCGTAGCGTATCGCCATGTATCACCCGCATGGCTTGCACCAGCTCCCGTAGCGTAT
CGCCATGTATCGCACGCATGGCTTGCACTAGCGCCCGTAGCGTATCGCCATGTATTGCCCGCATGGCTTGGGCATT
TCATAGGCATCTTTTGGGCGTGAGTTGGGCATTTGATGGCCGCCATGTATCGCCCGCATGGCTTGCACCAGCGCCC
GTAGCGTATCACCATGTATCGCCCGCATGGCTTGCACTAGCGCCCGTAGCGTATCACCATGTATCGCCCGTAGCGT
ATCGCCATGTATCGACCGCATGGCTTGCACTAGCGCCCATAGCGTATCGCCATGTATCGCCCGTAGCGTATCGCCA
TGTATCACCCGCATGGCTTGCACCAGCTCCCGTAGCGTATCGCCATGTATCGCCCGCATGGCTTGCACTAGCGCCC
GTAGCGTATCGCCATGTATCGCCCGCATGGCTTGGGCATTTGCTGGGCATCCTTGGGCATTGGCTAGGCGTTGCTT

GGGCATTGGCTGGGCGCTATGCATCCCCGGGCAAGATCACTCGGGGAATGAGACCTATGCATCGTCTGGTCAAGT
ATGGTTATGGTTCTCTCGGGGAACCTCAACCATGCATCACCACATAAGAGATATTGAGGTTGCATGGGTGATTCTA
TTGAGGCTGCGTGGGCGATGCATGGCTAGGCCGACGTGCATCCGGGAGGAAAATTCTTGAAAACACATCTAAATG
GTCCAATTTTTTTGTGACAACTTAGTAGTGGCTATTACTCATAAAAACACACCTAAGGCAAAAAGATTGAGAAAAA
AAGAGGTCCGGGGAAAAAAAATCGTAAAAAATCATTTTCGTCAATTCTGAGTATGAATACTCCATTATCCGCTTCC
AAAACATGTCTAGATGTGTAGTACGTTCGAAAACACCCCAAATGAAAAAAAAAAGTGTCAAAAAACGACACCGGA
GTGATAAGTTATGACATTTTGAATTTCAGTGTGCCCCAATGTTCCCTTTTCAGTACCCTGGAAAAATTTTAGTGGCT
ATATTAGGGGGGAGGTGTTGGTTGGATCCCTGGGAAAAAAAAAATTTCCCCCCCGAGTTGGTGAAAGGCCAGCA
ATGCCCACACGATGCATCGACCAATGCATGTGAGCGTGTGGGCATGTCCCGATGAGGTCCCTTTTGAGTTTCCAAC
ACCGAACAAACCACGTGGGGGCGTGGGAATGGCTCTCGATCACCCTATTGGTACGGGCGATCCTGGGCGACGGC
TGGGCAATGGCTGGGCGTTTGCTGGGCGTTTGCTGGGCATTCGCTGGGCGTTCGCTGGGCGTTCTCTGGGCGATA
CATGGGCCGGTGATTCGGCGTATCACCATAGGAGAATTCCTTACCATCGTCCATGGCACGTCACGAGATGGCATTG
GAAATATGGGGGATGCGTTGGTTGATGGCTTCTTGGACAAGAAAATCCCCCCGAGCTGGTGAAAGGTCGGCAAA
GACCCACACGTCTCCCTTGTAGCGCCCAATGCATGAGAGCGTGTGGGTATGTCCCGATGAGGCCCCTTCGAGATCA
CAACACCACGTAGGGGCGGAGGGATTGGCTCTCGATCACCCTATTGGCACGGGTGATCGTGGGCGACGGCTGGG
CGATGGCTGGGCGCTCTCTGGGCGTTCTCTGGGCGATACATGGGCCGGTGATTCGGCGTATCACCATGAGATAAT
TCCTTGCCATCGTCCATGGCGCGTCACGAGATGGTGTTGGCTGGATGGTTGGGCACATGGGCATAGCTTGGGAGA
TCCCCGGGCAATATGTGGGCATTGGATGGTTGGCGGATATCTTGTGAATGTTTTGAGATCAGCACGTGGAGCGTT
GCTCGGGCTGGTGCTGAGCAACCCCCAAATATGTGAGCTGGGTACTTTTTGTGGACGGTTGTCAATCCTCATGCTT
GGTAGCTATGAAATGAAATGTTCTCCATGCCTCTCCCTCGTATAACTTGCCCCGATACTATCAGGCGACTGTGTTTG
TCTCCCATGCAGCATCTTAGTCCGTATATCGCTCATCCTAATTACATTCGTTTGTAATGCTGCGGCGCAGGTGGCCA
AAGCGGGCGATGGAATAAGGTGTTGCATGGGGGTACTATAGGTCTCCCATGCAACATCTTAGTTCGTATATCGCTC
AATCCGGTTACATTCGTTTGTAACGCTGCGGCGCTGGTGGCCAAAGCGGGCGACGGAATAGGTTGTTGCATGGGG
GTACGCATGGTCTTCCATGCAGCATCTTAGTCCGTATATTGCTCACCCCGGTTACATTTGTTTGTAACGCTGCGGCG
CTGGTGGCCAAAGCGAGCGATGGAAAAAGTTGTTGCATGGTGGATCATGCGTACGTCTGCTCGCATGCACCATCT
TAGGCCTTGATTGCTCAGTCCGGTTACATTCGTTTGTAACGCTGCGGCGCTGGTGGCCAAAGCGGGCGATGGAAT
AAGGTGTTGCATGTGGGAAGGTCGTGGGCTGTTACTTTGATGCTGGGGGTTCACCATGCCTCTCTCCTGGATAACC
TTCCCGTTCCATTCCACGCGGCCATTTGACTTGTTTAAGGCGCTTCATGTGCGAAAAAAACATGGTCATCTAAACGA
GTGGTTTAGGATAGGGGGTGTGATGGGGGAGGAGTGTGTTGCTGCCCACCATGCATCAGCTTGGCCCGATACTAT
CAGGCGACCGTGTTAGTCTCCTATGCAGCATCTTAGTCCGTATATCGCTCAATCCGGTTACATTCGTTTGTAACGCT
GCGGCGCTGGTGGCCAAAGCGGGCGGTGGAATAGGTTGTTGCATGGGGGTGCGCATGGTCTCCCATGCAACATC
TTAGTCCGTATATTGCTCATCCTGGTTACATTCGTTTGTAACGCTGTGGTGCTGGTGGCCAAAGCGGGCGATGATG
GAATAAGGTGTTGCATGGGGGATCATGCGTACGTCTGCTCCCATGCACCATTTTAGGCCTTGATTGCTCAAATCGG
TTACATTTGTTTGTAACGCTGCGGCGCTGGTGGCCAAAGCGGGCGATGGAATAAGGTGTTGCATGGGGGAAATCA
TGGGCTGTTACTTTGATGCGGGGGGAACATTCAGGATATTGCTACTAGATTGCGTGTTGGGAAGACTACCGTGCC
CAATAACCCAGTTTGCACCCTTCCGACTCCACGTAGTGCAAGCACAAAATGTCCACGAGATGCCTGATACTGTTCC
GAAGCTTTTCATGTTGGTTTGAAACTTTTGCCCCGATAGTGTGTCCTGACCGTTGCAGCAAGATTTTGCGCACAGCG
AGCGTTTCGAATGGCGCCTCGTTCTACCATATGTATTGCCCTTTGCGGGGTGATGCACACATGGGTGAGCGGGCAT
TGTTCCCAACGCAACGTGTTGGCGTGTGAGTGGTAGTAGAGACATCTCTGCTTTTGGGCTCCGTGCTTCGCGCATC
GAACCATAAGCACATGTTTCCCTCATTGGCGTCCGGCGTACTTGACTGTATGCCGGCAAGTAAAATGGTTCCTGTG
TTGCCTACTCGGAGAAATTGGAAAATATCATTGCTAACGAGATTCATTGCCTTTGGTCGGCCAAAAGCTGGCCGAG
GGCAACATGTTAGTGTGGCTTGTGAAACCTACCGTGGCATTCGCCCGCGTGTCTGGCTAGCAAGCTCGCACGTGCT
TGGGGATATGGAAAACATAGATGGGTCAGGGTTTCAAACTCGGCCGATGCAATTGTAGCTAATTTTGTGACCGCC
GGACCGTAATGATAAGTCCTACTCCCTGCGTGACCAGGTGTAGTTAGGCGCGGCGCGGACAACCGGCGACATAAA

GGAATGCTACCTGGTTGATCCTGCCAGTAGTCATATGCTTGTCTCAAAGATTAAGCCATGCATGTGTAAGTATGAA
CTAATTCAGACTGTGAAACTGCGAATGGCTCATTAAATCAGTTATAGTTTGTTTGATGGTATATGCTACTCGGATAA
CCGTAGTAATTCTAGAGCTAATACGTGCAACAAACCCCGACTTCTGGAAGGGATGCATTTATTAGATAAAAGGTCA
ACGCGGGCTTGTCCCGTTGCTCTGATGATTCATGATAACTCGACGGATCGCACGGCCTTTGTGCCGGCGACGCATC
ATTCAAATTTCTGCCCTATCAACTTTCGATGGTAGGATAGTGGCCTACTATGGTGGTGACGGGTGACGGAGAATTA
GGGTTCGATTCCGGAGAGGGAGCCTGAGAAACGGCTACCACATCCAAGGAAGGCAGCAGGCGCGCAAATTACCC
AATCCTGACACGGGGAGGTAGTGACAATAAATAACAATACCGGGCTCTTAGAGTCTGGTAATTGGAATGAGTACA
ATCTAAATCCCTTAACGAGGATCCATTGGAGGGCAAGTCTGGTGCCAGCAGCCGCGGTAATTCCAGCTCCAATAG
CGTATATTTAAGTTGTTGCAGTTAAAAAGCTCGTAGTTGGACTTTGGGTTTGGTAGGCCGGTCCGCCCTTGTGGTG
TGCACCGGTCGACCAGTCCCTTCTACCGGCGATACGCTCCTGTCCTTAATTGGCCGGGTCGTGCCACCGGTGCTGT
TACTTTGAAGAAATTAGAGTGCTCAAAGCAAGCCCAAGCTCTGGATATATTAGCATGGGATAACATCATAGGATTT
CGGTCCTATTGCGTTGGCCTTCGGGATCGGAGTAATGATTAACAGGGACAGTCGGGGGCATTCGTATTTCATAGTC
AGAGGTGAAATTCTTGGATTTATGAAAGACGAACCACTGCGAAAGCATTTGCCAAGGATGTTTTCATTAATCAAGA
ACGAAAGTTGGGGGCTCGAAGACGATCAGATACCGTCCTAGTCTCAACCATAAACGATGCCGACCAGGGATCGGC
GGATGTTGCTTTTAGGACTCCGCCGGCACCTTATGAGAAATCAAAGTTTTTGGGTTCCGGGGGGAGTATGGTCGC
AAGGCTGAAACTTAAAGGAATTGACGGAAGGGCACCACCAGGAGTGGAGCCTGCGGCTTAATTTGACTCAACAC
GGGGAAACTTACCAGGTCCAGACATAGTAAGGATTGACAGACTGAGAGCTCTTTCTTGATTCTATGGGTGGTGGT
GCATGGCCGTTCTTAGTTGGTGGAGCGATTTGTCTGGTTAATTCCGTTAACGAACGAGACCTCAGCCTGCTAACTA
GCTATGTGGAGGTACCCTCCACGGCCAGCTTCTTAGAGGGACTATGGCCGTTTAGGCCACGGAAGTTTGAGGCAA
TAACAGGTCTGTGATGCCCTTAGATGTTCTGGGCCGCACGCGCGCTACACTGATGTATTCAACGAGTCTATAGCCT
TGGCCGACAGGCCCGGGTAATCTTTGAAAATTTCATCGTGATGGGGATAGATCATTGCAATTGTTGGTCTTCAACG
AGGAATTCCTAGTAAGCGCGAGTCATCAGCTCGCGTTGACTACGTCCCTGCCCTTTGTACACACCGCCCGTCGCTCC
TACCGATTGAATGGTCCGGTGAAATGTTCGGATCGCGGCAACGTGGGTGGTTCGCCGCCGGCGACGCCGCGAGA
AGTCCATTGAACCTTATCATTTAGAGGAAGGAGAAGTCGTAACAAGGTTTCCGTAGGTGAACCTGCGGAAGGATC
ATTGTCGATACCTGCTCAGCAGAACGACCCGCGAACACGTGAAAACAACCTACCATGCCCGGGAGCGGGCTCCGG
CCAGCGACCCGGTGCTGCAACAAAATCCGGCGCAACTGGCGTCAAGGAAAACTCACCGGAAGCAAGGCGTCGGC
TCGTTCGGCGCCGCGTGTCCGAATACTCAAACGACTCTCGGCAACGGATATCTCGGCTCTTGCATCGATGAAGAAC
GTAGCGAAATGCGATACTTGGTGTGAATTGCAGAATCCCGTGAACCATCGAGTCTTTGAACGCAAGTTGCGCCCG
AAGCCTTTTTGGCCGAGGGCACGCCTGCCTGGGCGTCACACACAGCGTCGCCCCCCACCAATCCCTCTGGATGGG
ATGGGGGGCGGAGATTGGCCCCCCGAGCCCTCCGGGGCACGGTCGGCATAAATGTTGGTCCCCGGCGGCGAGCG
TCGCGGTCAGCGGTGGTTGTATACTCATCCCCCGAGGACAAAATGACGCGCACGCCTCGTCGCTCGTCGGGCAGA
GCAAACCCCAGGGAGCACGGCTTCCACCTGCGACCCCAGGTCAGGCGGGATTACCCGCTGAGTTTAAGCATATCA
ATAAGCGGAGGAAAAGAAACTTACGAGGATTCCCCTAGTAACGGCGAGCGAACCGGGAATAGCCCAGCTTGGGA
ATCGGGCGACCCCGTCGTCCGAATTGTAGTCTGTAGAAGCGTCCTCAGCGGCGGACCGGGCCCAAGTCCCCTGGA
AGGTGGCGCCAGAGAGGGTGAGAGCCCCGTCGTGCCCGGACCCTGTCGCACCACGAGGCGCTGTCGGCGAGTCG
GGTTGTTTGGGAATGCAGCCCCAATCGGGCGGTAAATTCCGTCCAAGGCTAAATACTGGCGAGAGACCGATAGCG
AACAAGTACCGCGAGGGAAAGATGAAAAGGACTTTGAAAAGAGAGTCAAAGAGTGCTTGAAATTGTCGGGAGG
GAAGCGGATGGGGGCCGGCGATGTGCGCCGGTCGGATGCGGAACGGCGAGAGCTGGTCCGCCGCTCGGCTCGG
TGCGCGGACCGACGCGGATTGTGGAGGCGACCCAAGCCTGGGCCTCCTCGGAGGCCTGCGGAGATGTCGTCCCC
GCGATTGTGGTGGGCAGCACGCGCCTCACGGCGTGCCTCGGCAACTGCGTGCTCCCGGCGTCGGCCAGTGGGCTC
CCCATTCGGCCCGTCTTGAAACACGGACCAAGGAGTCTGACATGTGTGCGAGTCAGCGGGTGAGTAAACCCGTGA
GGCGCAAGGAAGCTAATTGGCGGGATCCCCCTGTGGGTTGCACCGCCGACCGACCTAGATCTTCTGTGAAGGGTT
CGAGTGTGAGCATACCTGTCGGGACCCGAAAGATGGTGAACTATGCCTGAGCGGGGCGAAGCCAGAGGAAACTC
TGGTGGAGGCCCGCAGCGATACTGACGTGCAAATCGTTCGTCTGACTTGGGTATAGGGGCGAAAGACTAATCGA

ACCATCTAGTAGCTGGTTCCCTCCGAAGTTTCCCTCAGGATAGCTGGAGCTCGCGGGCGAGTTCTATCAGGTAAAG
CCAATGATTAGAGGCATCGGGGGCGCAACGCCCTCGACCTATTCTCAAACTTTAAATAGGTAGGACGGCGCGGCT
ACTTCGCTGAGCCGCGCCACGGAATCGAGTGCTCCAAGTGGGCCATTTTTGGTAAGCAGAACTGGCGATGCGGGA
TGAACCGGAAGCCGGGTTACGGTGCCCAACTGCGCGCTAACCCAGAACCCACAAAGGGTGTTGGTCGATTAAGAC
AGCAGGACGGTGGTCATGGAAGTCGAAATCCGCTAAGGAGTGTGTAACAACTCACCTGCCGAATCAACTAGCCCC
GAAAATGGATGGCGCTGAAGCGCGCGACCCACACCCGGCCGTCAGGGCAATTGTTAGGCCCTGATGAGTAGGAG
GGCGCAGCGGTGGCCGCGAAACCCAGGGCGCGAGCCCGGGTGGAGCCGCCGTTGGTGCAGATCTTGGTGGTAG
TAGCAAATATTCAAATGAGAACTTTGAAGGCCGAAGAGGGGAAAGGTTCCATGTGAACGGCACTTGCACATGGGT
TAGTCGATCCTAAGAGACGGCCGAAAGGCGTCAGAGAGCGTGCATCACGCGAGCTTCGAAAGGGAATCGGGTTA
AAATTCCTGAACCGGGACACAGTGGTCGACGGCAACGCTAGGAAGTCCGGAGACGTCGGCGGGGGCCTCGGGA
AGAGTTATCTTTTCTGTTTAACAGCCTGCCCACCCTGGAAACGGCTCAGCCGGAGGTAGGGTCCAGCGGCTGGAA
GAGCACCGCACGTCGCGTGGTGTCCGGTGCGCCCCCGGCGGCCCTTGAAAATCCGGAGGACCGAATGCCAACTGT
GCCCGGTCGTACTCATAACCGCATCAGGTCTCCAAGGTGAACAGCCTCTGGTCGATGGAACAATGTAGGCAAGGG
AAGTCGGCAAAATGGATCCGTAACCTCGGGAAAAGGATTGGCTCTGAGGGCTGGGCACGGGGGTCCCAGTCCCG
AACCCGTTGGCTGCCGGCGGACTGCTCGAGCTGCTTCCGTGGCGAGAGCGGGTCGCCGCGTGCCGGCCGGGGGA
TGGACTGGGAACGGCTCCTTCGGGGGCCTTCCCCGGGCGTCGAACAGCCAACTCAGAACTGGTACGGACAAGGG
GAATCCGACTGTTTAATTAAAACAAAGCATTGCGATGGTCCCTGCGGATGCTAACGCAATGTGATTTCTGCCCAGT
GCTCTGAATGTCAAAGTGAAGAAATTCAACCAAGCGCGGGTAAACGGCGGGAGTAACTATGACTCTCTTAAGGTA
GCCAAATGCCTCGTCATCTAATTAGTGACGCGCATGAATGGATTAACGAGATTCCCACTGTCCCTGTCTACTATCCA
GCGAAACCACAGCCAAGGGAACGGGCTTGGCAGAATCAGCGGGGAAAGAAGACCCTGTTGAGCTTGACTCTAGT
CCGACTTTGTGAAATGACTTGAGAGGTGTAGGATAAGTGGGAGCCGACTCGTGCGGCGAAAGTGAAATACCACT
ACTTTTAACGTTATTTTACTTATTCCGTGAATCGGAAGCGGGGCACTGCTCCTCTTTTTGGATCCAAGTTCGGTCTC
GACGGGACAATCCGGGCGGAAGACATTGTCAGGTGGGGAGTTTGGCTGGGGCGGCACATCTGTTAAAAGATAAC
GCAGGTGTCCTAAGATGAGCTCAACGAGAACAGAAATCTCGTGTGGAACAAAAGGGTAAAAGCTCGTTTGATTCT
GATTTCCAGTACGAATACGAACCGTGAAAGCGTGGCCTATCGATCCTTTAGACCTTCGGAATTTGAAGCTAGAGGT
GTCAGAAAAGTTACCACAGGGATAACTGGCTTGTGGCAGCCAAGCGTTCATAGCGACGTTGCTTTTTGATCCTTCG
ATGTCGGCTCTTCCTATCATTGTGAAGCAGAATTCACCAAGTGTTGGATTGTTCACCCACCAATAGGGAACGTGAG
CTGGGTTTAGACCGTCGTGAGACAGGTTAGTTTTACCCTACTGATGATAGTGTCGCGATAGTAATTCAACCTAGTA
CGAGAGGAACCGTTGATTCACACAATTGGTCATCGCGCTTGGTTGAAAAGCCAGTGGCGCGAAGCTACCGTGTGC
AGGATTATGACTGAACGCCTCTAAGTCAGAATCCAGGCTAGAGAAGCGACGCATGCGCCCGTCGCCCGATTGCCG
ACCTGCAGTAGGGGTCCTTGGACCCCCAAAGGCACGTGTCACTGGCGTAGCTCCCGTGGCCGATGAGTCGCGTGG
TGCCGCCTTGAAGTATAATTCCTACCGAGCGGCGGGCTGAATCCTTTGCAGACGACTTAAATACGCGACGGGGTA
TTGTAAGTGGCAGAGTGGCCTTGCTGCCACGATCCACTGAGATTCAGCCCCATGTCGCATCGATTCGTCCCTCCCC
ATCCAAAAAAA

>A_sylvestris_2/1-12084

ACTACAACACACCAATTGCCTCGTATACCACTCGAGGTTACAACTATTTTACGAGAGAGTGATACTTGGAAAAAAT
TCTAAGTCTGGGAAATAAATACTCCACTTCATATGTAAGTGCCATCGCCCGGACAAACCATGGTCGAGTATCATCG
CCAGTAGGGCTTGTACGAGCGCCCGTAGCGTATCACTATGTATAGTGAACATGGCTTGCACTAGCTCCCGTAGCGT
ATTGCCATGTATCACCCGCATGGCTTGCACTAGCCCCTGTAACGTATCGCCATGTATCGCCCGTAGCGTATCGCCAT
GTATCGCCCGCATGGCTTGCACTAGCCTGTGGGCAAATATGCATCGACTTCCCCGACGATGTTTGTGAGATGGATC
AACCGAGGAAATATGTCACGGGATAGCATATCCCCAAATAAATATGCACAATGGCATGAATCACCTATGCAAATA
ACCCCTGTCATGATGATGCTTGTGAAATGGGCAACTATTGGGCATTTCATAGGCATCGTTTGGGCATGAACTGGGC

ATTTGCTGGCCACCATGTATCGCCCGCATGCCTTGTACTAGCGCCCGTAGCGTATCGCCATGTATCACCCGCATGGC
TTGCACTAGCTCCCGTCGCGTATCGCCATGTATCGCCCGTAGTGTATCGCCATGTATCACCCGCATGGCTTGCACCA
GCTCCCGTAGCGTATCGCCATGTATCGACCGTAGTGTATCGCCATGTATCACCCGCATGGCTTGCACCAGCTCCCGT
AGCGTATCGCCATGTATCGCCCGTAGCGTATCGCCATGTATTGCCCGCATAGCTTGGGCATTTCCTAGGCATCTTTT
GGGCGTGTGCTGGGCATTTGATAGCCGTCATGTATCGCCCGCATGGCTTGTACCAACCCCCGTAGCGTATCGCCAT
GTATCGCCCGTAGCGTATCGCCATGTATCACCCGCATGGCTTGCACCAACCCCCGTAGCGTATCGCCATGTATCGC
CCGTAGCGTATCGCCATGGCTTGCATTAGCGCCCGTAGCGTATCGCCATGGCTTGCATTAGCGCCCGTTGCGTATC
GCCATGTATCGCCCGTAGCGTATCGCCATGTATCACCCGCATGGCTTGCACCAGCTCCCGTAGCGTATCGCCATGT
ATCGCCCGCATGGCTTACACTAGCGCCCGTAGCGTATCGCCATGTATTGCCCGCATGGCTTGGGCATTTCATAGGC
ATCTGTTGGGCGTGAGCTGGGCATTTGATGGGCACCATGTATCGCCCGCAAGGCTTGCACCAGTGCCCGTAGCGT
ATCGCCATGTATCGCCCGTAGCGTATCGCCATGTATCACCCGCATGGCTTGCACCAGCTCCCGTAGCGTATCGCCAT
GAATCGCCCGTAGCGTATCGCCATGTATTGCCCGCATAGCTTGGGCATTTCCTAGGCATCATTTGGGCGTGAGCTG
GGCATTTGATGGCCGTCATGTATCGCCCGCATGGCTTGCACCAGCCCCCTAGCGTATCGCCATGTATCGCCCGTAG
CGTATCGCCATGTATCACCCGCATGCTTGCACCAGCTCCCGTAGCGTATCGCCATGTATCGCCCACATGGCTTGCAC
TAGCTCCCGTAGCGTATCGCCATGTATCGCCCGTAGCGTATCGCCATGTATCACCCGCATGGCTTGCACCAGCTCCC
GTAGCGTATCGCCATGTATCGCCCGCATGGCTTACACTAGCGCCCGTAGCGTATCGCCATGTATTGCCCGCATGGC
TTGGGCATTTCATAGGCATCTGTTGGGCGTGAGCTGGGCATTTGATGGGCACCATGTATCGCCCGCAAGGCTTGC
ACCAGTGCCCGTAGCGTATCGCCATGTATCGCCCGTAGCGTATCGCCATGTATCACCCGCATGGCTTGCACCAGCT
CCCGTAGCGTATCGCCATGTATCGCCCGTAGCGTATCGCCATGTATTGCCCGCATAGCTTGGGCATTTCCTAGGCAT
CTTTTGGGCGTGTGCTGGGCATTTGATAGCCGTCATGTATCGCCCGCATGGCTTGCACTAGCTCCCGTAGCGTATC
GCCATGTATCGCCCGTAGCGTATCGCCATGTATCACCCGCATGGCTTGCACCAGCTCCCGTAGCGTATCGCCATGT
ATCGCCCGCATGGCTTACACCAGCGCCCGTAGCGTATCGCCATGTATCGCCCGCATGGCTTGCACCAGCGCCCGTA
GCGTATCGCCATGTATCGCCCGTAGCGTATCGCCATGTATCACCCGCATGGCTTGCACCACCTCCCGTAGCGTATC
GCCATGTATCGCACACATGGCTTGCACTAGCGCCCGTAGCGTATCGCCATGTATTGCCCGCATGGCTTGGGCATTT
CATAGGCATCTCTTGGGCGTGAGCTGGGCATTTGATGGCCGCCATGTATCGCCCGCATGGTTGCACCCGCGCCCGT
AGCGTATCGCCATGTATCGCCCGTAGCGTATCGCCATGTATCACCCGCATGGCTTGCACCAGCTCCCGTAGCGTAT
CGCCATGTATCGCACGCATGGCTTGCACTAGCGCCCGTAGCGTATCGCCATGTATTGCCCGCATGGCTTGGGCATT
TCATAGGCATCTTTTGGGCGTGAGTTGGGCATTTGATGGCCGCCATGTATCGCCCGCATGGCTTGCACCAGCGCCC
GTAGCGTATCACCATGTATCGCCCGCATGGCTTGCACTAGCGCCCGTAGCGTATCACCATGTATCGCCCGTAGCGT
ATCGCCATGTATCGACCGCATGGCTTGCACTAGCGCCCATAGCGTATCGCCATGTATCGCCCGTAGCGTATCGCCA
TGTATCACCCGCATGGCTTGCACCAGCTCCCGTAGCGTATCGCCATGTATCGCCCGCATGGCTTGCACTAGCGCCC
GTAGCGTATCGCCATGTATCGCCCGCATGGCTTGGGCATTTGCTGGGCATCCTTGGGCATTGGCTAGGCGTTGCTT
GGGCATTGGCTGGGCGCTATGCATCCCCGGGCAAGATCACTCGGGGAATGAGACCTATGCATCGTCTGGTCAAGT
ATGGTTATGGTTCTCTCGGGGAACCTCAACCATGCATCACCACATAAGAGATATTGAGGTTGCATGGGTGATTCTA
TTGAGGCTGCGTGGGCGATGCATGGCTAGGCCGACGTGCATCCGGGAGGAAAATTCTTGAAAACACATCTAAATG
GTCCAATTTTTTTGTGACAACTTAGTAGTGGCTATTACTCATAAAAACACACCTAAGGCAAAAAGATTGAGAAAAA
AAGAGGTCCGGGGAAAAAAAATCGTAAAAAATCATTTTCGTCAATTCTGAGTATGAATACTCCATTATCCGCTTCC
AAAACATGTCTAGATGTGTAGTACGTTCGAAAACACCCCAAATGAAAAAAAAAAGTGTCAAAAAACGACACCGGA
GTGATAAGTTATGACATTTTGAATTTCAGTGTGCCCCAATGTTCCCTTTTCAGTACCCTGGAAAAATTTTAGTGGCT
ATATTAGGGGGGAGGTGTTGGTTGGATCCCTGGGAAAAAAAAAATTTCCCCCCCGAGTTGGTGAAAGGCCAGCA
ATGCCCACACGATGCATCGACCAATGCATGTGAGCGTGTGGGCATGTCCCGATGAGGTCCCTTTTGAGTTTCCAAC
ACCGAACAAACCACGTGGGGGCGTGGGAATGGCTCTCGATCACCCTATTGGTACGGGCGATCCTGGGCGACGGC
TGGGCAATGGCTGGGCGTTTGCTGGGCGTTTGCTGGGCATTCGCTGGGCGTTCGCTGGGCGTTCTCTGGGCGATA
CATGGGCCGGTGATTCGGCGTATCACCATAGGAGAATTCCTTACCATCGTCCATGGCACGTCACGAGATGGCATTG

GAAATATGGGGGATGCGTTGGTTGATGGCTTCTTGGACAAGAAAATCCCCCCGAGCTGGTGAAAGGTCGGCAAA
GACCCACACGTCTCCCTTGTAGCGCCCAATGCATGAGAGCGTGTGGGTATGTCCCGATGAGGCCCCTTCGAGATCA
CAACACCACGTAGGGGCGGAGGGATTGGCTCTCGATCACCCTATTGGCACGGGTGATCGTGGGCGACGGCTGGG
CGATGGCTGGGCGCTCTCTGGGCGTTCTCTGGGCGATACATGGGCCGGTGATTCGGCGTATCACCATGAGATAAT
TCCTTGCCATCGTCCATGGCGCGTCACGAGATGGTGTTGGCTGGATGGTTGGGCACATGGGCATAGCTTGGGAGA
TCCCCGGGCAATATGTGGGCATTGGATGGTTGGCGGATATCTTGTGAATGTTTTGAGATCAGCACGTGGAGCGTT
GCTCGGGCTGGTGCTGAGCAACCCCCAAATATGTGAGCTGGGTACTTTTTGTGGACGGTTGTCAATCCTCATGCTT
GGTAGCTATGAAATGAAATGTTCTCCATGCCTCTCCCTCGTATAACTTGCCCCGATACTATCAGGCGACTGTGTTTG
TCTCCCATGCAGCATCTTAGTCCGTATATCGCTCATCCTAATTACATTCGTTTGTAATGCTGCGGCGCAGGTGGCCA
AAGCGGGCGATGGAATAAGGTGTTGCATGGGGGTACTATAGGTCTCCCATGCAACATCTTAGTTCGTATATCGCTC
AATCCGGTTACATTCGTTTGTAACGCTGCGGCGCTGGTGGCCAAAGCGGGCGACGGAATAGGTTGTTGCATGGGG
GTACGCATGGTCTTCCATGCAGCATCTTAGTCCGTATATTGCTCACCCCGGTTACATTTGTTTGTAACGCTGCGGCG
CTGGTGGCCAAAGCGAGCGATGGAAAAAGTTGTTGCATGGTGGATCATGCGTACGTCTGCTCGCATGCACCATCT
TAGGCCTTGATTGCTCAGTCCGGTTACATTCGTTTGTAACGCTGCGGCGCTGGTGGCCAAAGCGGGCGATGGAAT
AAGGTGTTGCATGTGGGAAGGTCGTGGGCTGTTACTTTGATGCTGGGGGTTCACCATGCCTCTCTCCTGGATAACC
TTCCCGTTCCATTCCACGCGGCCATTTGACTTGTTTAAGGCGCTTCATGTGCGAAAAAAACATGGTCATCTAAACGA
GTGGTTTAGGATAGGGGGTGTGATGGGGGAGGAGTGTGTTGCTGCCCACCATGCATCAGCTTGGCCCGATACTAT
CAGGCGACCGTGTTAGTCTCCTATGCAGCATCTTAGTCCGTATATCGCTCAATCCGGTTACATTCGTTTGTAACGCT
GCGGCGCTGGTGGCCAAAGCGGGCGGTGGAATAGGTTGTTGCATGGGGGTGCGCATGGTCTCCCATGCAACATC
TTAGTCCGTATATTGCTCATCCTGGTTACATTCGTTTGTAACGCTGTGGTGCTGGTGGCCAAAGCGGGCGATGATG
GAATAAGGTGTTGCATGGGGGATCATGCGTACGTCTGCTCCCATGCACCATTTTAGGCCTTGATTGCTCAAATCGG
TTACATTTGTTTGTAACGCTGCGGCGCTGGTGGCCAAAGCGGGCGATGGAATAAGGTGTTGCATGGGGGAAATCA
TGGGCTGTTACTTTGATGCGGGGGGAACATTCAGGATATTGCTACTAGATTGCGTGTTGGGAAGACTACCGTGCC
CAATAACCCAGTTTGCACCCTTCCGACTCCACGTAGTGCAAGCACAAAATGTCCACGAGATGCCTGATACTGTTCC
GAAGCTTTTCATGTTGGTTTGAAACTTTTGCCCCGATAGTGTGTCCTGACCGTTGCAGCAAGATTTTGCGCACAGCG
AGCGTTTCGAATGGCGCCTCGTTCTACCATATGTATTGCCCTTTGCGGGGTGATGCACACATGGGTGAGCGGGCAT
TGTTCCCAACGCAACGTGTTGGCGTGTGAGTGGTAGTAGAGACATCTCTGCTTTTGGGCTCCGTGCTTCGCGCATC
GAACCATAAGCACATGTTTCCCTCATTGGCGTCCGGCGTACTTGACTGTATGCCGGCAAGTAAAATGGTTCCTGTG
TTGCCTACTCGGAGAAATTGGAAAATATCATTGCTAACGAGATTCATTGCCTTTGGTCGGCCAAAAGCTGGCCGAG
GGCAACATGTTAGTGTGGCTTGTGAAACCTACCGTGGCATTCGCCCGCGTGTCTGGCTAGCAAGCTCGCACGTGCT
TGGGGATATGGAAAACATAGATGGGTCAGGGTTTCAAACTCGGCCGATGCAATTGTAGCTAATTTTGTGACCGCC
GGACCGTAATGATAAGTCCTACTCCCTGCGTGACCAGGTGTAGTTAGGCGCGGCGCGGACAACCGGCGACATAAA
GGAATGCTACCTGGTTGATCCTGCCAGTAGTCATATGCTTGTCTCAAAGATTAAGCCATGCATGTGTAAGTATGAA
CTAATTCAGACTGTGAAACTGCGAATGGCTCATTAAATCAGTTATAGTTTGTTTGATGGTATATGCTACTCGGATAA
CCGTAGTAATTCTAGAGCTAATACGTGCAACAAACCCCGACTTCTGGAAGGGATGCATTTATTAGATAAAAGGTCA
ACGCGGGCTTGTCCCGTTGCTCTGATGATTCATGATAACTCGACGGATCGCACGGCCTTTGTGCCGGCGACGCATC
ATTCAAATTTCTGCCCTATCAACTTTCGATGGTAGGATAGTGGCCTACTATGGTGGTGACGGGTGACGGAGAATTA
GGGTTCGATTCCGGAGAGGGAGCCTGAGAAACGGCTACCACATCCAAGGAAGGCAGCAGGCGCGCAAATTACCC
AATCCTGACACGGGGAGGTAGTGACAATAAATAACAATACCGGGCTCTTAGAGTCTGGTAATTGGAATGAGTACA
ATCTAAATCCCTTAACGAGGATCCATTGGAGGGCAAGTCTGGTGCCAGCAGCCGCGGTAATTCCAGCTCCAATAG
CGTATATTTAAGTTGTTGCAGTTAAAAAGCTCGTAGTTGGACTTTGGGTTTGGTAGGCCGGTCCGCCCTTGTGGTG
TGCACCGGTCGACCAGTCCCTTCTACCGGCGATACGCTCCTGTCCTTAATTGGCCGGGTCGTGCCACCGGTGCTGT
TACTTTGAAGAAATTAGAGTGCTCAAAGCAAGCCCAAGCTCTGGATATATTAGCATGGGATAACATCATAGGATTT
CGGTCCTATTGCGTTGGCCTTCGGGATCGGAGTAATGATTAACAGGGACAGTCGGGGGCATTCGTATTTCATAGTC

AGAGGTGAAATTCTTGGATTTATGAAAGACGAACCACTGCGAAAGCATTTGCCAAGGATGTTTTCATTAATCAAGA
ACGAAAGTTGGGGGCTCGAAGACGATCAGATACCGTCCTAGTCTCAACCATAAACGATGCCGACCAGGGATCGGC
GGATGTTGCTTTTAGGACTCCGCCGGCACCTTATGAGAAATCAAAGTTTTTGGGTTCCGGGGGGAGTATGGTCGC
AAGGCTGAAACTTAAAGGAATTGACGGAAGGGCACCACCAGGAGTGGAGCCTGCGGCTTAATTTGACTCAACAC
GGGGAAACTTACCAGGTCCAGACATAGTAAGGATTGACAGACTGAGAGCTCTTTCTTGATTCTATGGGTGGTGGT
GCATGGCCGTTCTTAGTTGGTGGAGCGATTTGTCTGGTTAATTCCGTTAACGAACGAGACCTCAGCCTGCTAACTA
GCTATGTGGAGGTACCCTCCACGGCCAGCTTCTTAGAGGGACTATGGCCGTTTAGGCCACGGAAGTTTGAGGCAA
TAACAGGTCTGTGATGCCCTTAGATGTTCTGGGCCGCACGCGCTACACTGATGTATTCAACGAGTCTATAGCCT
TGGCCGACAGGCCCGGGTAATCTTTGAAAATTTCATCGTGATGGGGATAGATCATTGCAATTGTTGGTCTTCAACG
AGGAATTCCTAGTAAGCGCGAGTCATCAGCTCGCGTTGACTACGTCCCTGCCCTTTGTACACACCGCCCGTCGCTCC
TACCGATTGAATGGTCCGGTGAAATGTTCGGATCGCGGCAACGTGGGTGGTTCGCCGCCGGCGACGCCGCGAGA
AGTCCATTGAACCTTATCATTTAGAGGAAGGAGAAGTCGTAACAAGGTTTCCGTAGGTGAACCTGCGGAAGGATC
ATTGTCGATACCTGCTCAGCAGAACGACCCGCGAACACGTGAAAACAACCTACCATGCCCGGGAGCGGGCTCCGG
CCAGCGACCCGGTGCTGCAACAAAATCCGGCGCAACTGGCGTCAAGGAAAACTCACCGGAAGCAAGGCGTCGGC
TCGTTCGGCGCCGCGTGTCCGAATACTCAAACGACTCTCGGCAACGGATATCTCGGCTCTTGCATCGATGAAGAAC
GTAGCGAAATGCGATACTTGGTGTGAATTGCAGAATCCCGTGAACCATCGAGTCTTTGAACGCAAGTTGCGCCCG
AAGCCTTTTTGGCCGAGGGCACGCCTGCCTGGGCGTCACACACAGCGTCGCCCCCCACCAATCCCTCTGGATGGG
ATGGGGGGCGGAGATTGGCCCCCCGAGCCCTCCGGGGCACGGTCGGCATAAATGTTGGTCCCCGGCGGCGAGCG
TCGCGGTCAGCGGTGGTTGTATACTCATCCCCCGAGGACAAAATGACGCGCACGCCTCGTCGCTCGTCGGGCAGA
GCAAACCCCAGGGAGCACGGCTTCCACCTGCGACCCCAGGTCAGGCGGGATTACCCGCTGAGTTTAAGCATATCA
ATAAGCGGAGGAAAAGAAACTTACGAGGATTCCCCTAGTAACGGCGAGCGAACCGGGAATAGCCCAGCTTGGGA
ATCGGGCGACCCCGTCGTCCGAATTGTAGTCTGTAGAAGCGTCCTCAGCGGCGGACCGGGCCCAAGTCCCCTGGA
AGGTGGCGCCAGAGAGGGTGAGAGCCCCGTCGTGCCCGGACCCTGTCGCACCACGAGGCGCTGTCGGCGAGTCG
GGTTGTTTGGGAATGCAGCCCCAATCGGGCGGTAAATTCCGTCCAAGGCTAAATACTGGCGAGAGACCGATAGCG
AACAAGTACCGCGAGGGAAAGATGAAAAGGACTTTGAAAAGAGAGTCAAAGAGTGCTTGAAATTGTCGGGAGG
GAAGCGGATGGGGGCCGGCGATGTGCGCCGGTCGGATGCGGAACGGCGAGAGCTGGTCCGCCGCTCGGCTCGG
TGCGCGGACCGACGCGGATTGTGGAGGCGACCCAAGCCTGGGCCTCCTCGGAGGCCTGCGGAGATGTCGTCCCC
GCGATTGTGGTGGGCAGCACGCGCCTCACGGCGTGCCTCGGCAACTGCGTGCTCCCGGCGTCGGCCAGTGGGCTC
CCCATTCGGCCCGTCTTGAAACACGGACCAAGGAGTCTGACATGTGTGCGAGTCAGCGGGTGAGTAAACCCGTGA
GGCGCAAGGAAGCTAATTGGCGGGATCCCCCTGTGGGTTGCACCGCCGACCGACCTAGATCTTCTGTGAAGGGTT
CGAGTGTGAGCATACCTGTCGGGACCCGAAAGATGGTGAACTATGCCTGAGCGGGGCGAAGCCAGAGGAAACTC
TGGTGGAGGCCCGCAGCGATACTGACGTGCAAATCGTTCGTCTGACTTGGGTATAGGGGCGAAAGACTAATCGA
ACCATCTAGTAGCTGGTTCCCTCCGAAGTTTCCCTCAGGATAGCTGGAGCTCGCGGGCGAGTTCTATCAGGTAAAG
CCAATGATTAGAGGCATCGGGGGCGCAACGCCCTCGACCTATTCTCAAACTTTAAATAGGTAGGACGGCGCGGCT
ACTTCGCTGAGCCGCGCCACGGAATCGAGTGCTCCAAGTGGGCCATTTTTGGTAAGCAGAACTGGCGATGCGGGA
TGAACCGGAAGCCGGGTTACGGTGCCCAACTGCGCGCTAACCCAGAACCCACAAAGGGTGTTGGTCGATTAAGAC
AGCAGGACGGTGGTCATGGAAGTCGAAATCCGCTAAGGAGTGTGTAACAACTCACCTGCCGAATCAACTAGCCCC
GAAAATGGATGGCGCTGAAGCGCGCGACCCACACCCGGCCGTCAGGGCAATTGTTAGGCCCTGATGAGTAGGAG
GGCGCAGCGGTGGCCGCGAAACCCAGGGCGCGAGCCCGGGTGGAGCCGCCGTTGGTGCAGATCTTGGTGGTAG
TAGCAAATATTCAAATGAGAACTTTGAAGGCCGAAGAGGGGAAAGGTTCCATGTGAACGGCACTTGCACATGGGT
TAGTCGATCCTAAGAGACGGCCGAAAGGCGTCAGAGAGCGTGCATCACGCGAGCTTCGAAAGGGAATCGGGTTA
AAATTCCTGAACCGGGACACAGTGGTCGACGGCAACGCTAGGAAGTCCGGAGACGTCGGCGGGGGCCTCGGGA
AGAGTTATCTTTTCTGTTTAACAGCCTGCCCACCCTGGAAACGGCTCAGCCGGAGGTAGGGTCCAGCGGCTGGAA
GAGCACCGCACGTCGCGTGGTGTCCGGTGCGCCCCCGGCGGCCCTTGAAAATCCGGAGGACCGAATGCCAACTGT

GCCCGGTCGTACTCATAACCGCATCAGGTCTCCAAGGTGAACAGCCTCTGGTCGATGGAACAATGTAGGCAAGGG
AAGTCGGCAAAATGGATCCGTAACCTCGGGAAAAGGATTGGCTCTGAGGGCTGGGCACGGGGGTCCCAGTCCCG
AACCCGTTGGCTGCCGGCGGACTGCTCGAGCTGCTTCCGTGGCGAGAGCGGGTCGCCGCGTGCCGGCCGGGGGA
TGGACTGGGAACGGCTCCTTCGGGGGCCTTCCCCGGGCGTCGAACAGCCAACTCAGAACTGGTACGGACAAGGG
GAATCCGACTGTTTAATTAAAACAAAGCATTGCGATGGTCCCTGCGGATGCTAACGCAATGTGATTTCTGCCCAGT
GCTCTGAATGTCAAAGTGAAGAAATTCAACCAAGCGCGGGTAAACGGCGGGAGTAACTATGACTCTCTTAAGGTA
GCCAAATGCCTCGTCATCTAATTAGTGACGCGCATGAATGGATTAACGAGATTCCCACTGTCCCTGTCTACTATCCA
GCGAAACCACAGCCAAGGGAACGGGCTTGGCAGAATCAGCGGGGAAAGAAGACCCTGTTGAGCTTGACTCTAGT
CCGACTTTGTGAAATGACTTGAGAGGTGTAGGATAAGTGGGAGCCGACTCGTGCGGCGAAAGTGAAATACCACT
ACTTTTAACGTTATTTTACTTATTCCGTGAATCGGAAGCGGGGCACTGCTCCTCTTTTTGGATCCAAGTTCGGTCTC
GACGGGACAATCCGGGCGGAAGACATTGTCAGGTGGGGAGTTTGGCTGGGGCGGCACATCTGTTAAAAGATAAC
GCAGGTGTCCTAAGATGAGCTCAACGAGAACAGAAATCTCGTGTGGAACAAAAGGGTAAAAGCTCGTTTGATTCT
GATTTCCAGTACGAATACGAACCGTGAAAGCGTGGCCTATCGATCCTTTAGACCTTCGGAATTTGAAGCTAGAGGT
GTCAGAAAAGTTACCACAGGGATAACTGGCTTGTGGCAGCCAAGCGTTCATAGCGACGTTGCTTTTTGATCCTTCG
ATGTCGGCTCTTCCTATCATTGTGAAGCAGAATTCACCAAGTGTTGGATTGTTCACCCACCAATAGGGAACGTGAG
CTGGGTTTAGACCGTCGTGAGACAGGTTAGTTTTACCCTACTGATGATAGTGTCGCGATAGTAATTCAACCTAGTA
CGAGAGGAACCGTTGATTCACACAATTGGTCATCGCGCTTGGTTGAAAAGCCAGTGGCGCGAAGCTACCGTGTGC
AGGATTATGACTGAACGCCTCTAAGTCAGAATCCAGGCTAGAGAAGCGACGCATGCGCCCGTCGCCCGATTGCCG
ACCTGCAGTAGGGGTCCTTGGACCCCCAAAGGCACGTGTCACTGGCGTAGCTCCCGTGGCCGATGAGTCGCGTGG
TGCCGCCTTGAAGTATAATTCCTACCGAGCGGCGGGCTGAATCCTTTGCAGACGACTTAAATACGCGACGGGGTA
TTGTAAGTGGCAGAGTGGCCTTGCTGCCACGATCCACTGAGATTCAGCCCCATGTCGCATCGATTCGTCCCTCCCC
ATCCAAAAAAA

>A_parviflora_HC/1-11269

ACAACAACTCACCACCAATACCCTCGTGTACCCCTCGAGGTTACAGCCGCAATACGTGAAAGGGATACTTGGAAGA
AAAAAAAAGTCTAAGTATGGGAAATAAAAACTCCACTGATTATGCCCAGCCCGGCGCAAGGGCAGACGAGGCAA
GTGCACATGCATCGTCGGGCCAAGGATATGTATCACTTGGGCAAGCGACGTTGCCCGAGAAGGAAGCTGATGCAT
CGCCCGGGCAATGACGGCTATGCATCACTCGGGGAAGGATGACTATGCATCATGGGGGAAAGGATGCTTATGCA
TCGCCGGATCAAGGATAATCGTGTATCACCCGGGCAAATATGCCCATGGAGTCCCGAGGGTGCTGTGAGATGGAT
CACCCGCGGTAATATGCCCCGACCCTCCGGGCAAATATGAAACGGAGCCCCGACAGCGCTTCTGAGATGTATCAC
CCCGGAAAATATGCCCTGGCGTAGCATATCGCCCGGGTAAATATGCCTCGGTAGCTCCAACGATGCTTATGAGAT
GGATCACCCAGGGAAATATGCCCGAGAGTAGCATAACTCTCGGGCAAATATGCATCGGCGGCCCCGCCGAAATAT
GCCTGATGGATCAACCCGGGGTAATATGTCCCGGGGTAGCATATCCCCAAGCAAATATGCCCCTGGAGTCCAGAG
GGTGCTGTGAGTTTGATCACCCGGGGTAATATGCCCCCGCGTAGCATATCACCCAGGGGAATATCCACCTGGAGT
CCTAACAGTGCTTGTGAGAGGGATCACCCGGGGAAATATGCCACAACATAGCATATCGCCCCGGCGAAAATGCCT
CGGTAGCCCCAACGGTGCTTGTCAGATGAATCACCCCGGCTGATATGCCCCGACGTAGCATATCACTCCGGCTGAT
ATGCCCCGACGATGCTTGTGAGAGGGATCACCCGGACAAATATGCCCCGACATAGCATATCGTACAGACAAAAAT
GCCTTGGTAGCCCCGGTGGTGCTTATGAGATGAATCACCTGGGCGCCAAGCATCGGCCGGGCAAAAATGCCCCAT
GCGTGGCCTTGGCAAGGCACGCCCGCATGGCTTGCACTAGCTCCCATAGCGTATCGTCGTGTCGAGGGCCATGCA
TCGCCCGGGGCGTTGCGTATCGCCGCATGGCTTGCACTAGCGCCCGTAGCGCATCGCCGTGTCAAGGGCCATGCA
TCGCCCGGGGCGATGCGTATCGCCGCATGGCCCTCGACACGGGCCGATGCGTATCGCCGCATGGCTTGCACTAGG
GCCCATACCGTATCGCCGTGTCGAGGGCCATGCATCGCCCGGGGCGTTGCGTATCGCCGCATGGCCCTCGACACG
GGGCGATGCGGATCGCTACATGGCCCTCGACAAGGGGCGATGCGTATCGCCGCATGGCTTGCACTAGCGCCCGTA

GCGTATCGCCGTGTCGAGGGCCATGCATCGCCCGGGGCGATGCGTATCGCCGCATGGCTTGCACTAGCGCCCGTA
GCGTATCGCCGTGTCGAGGGCCATGCATCGCCCGGGGCGTTGCGTATCGCCGCATGGCCCTCGACACGGGGCGAT
GCGGATCGCCGCATGGCCCTCGACACTTGGCGATGCGGATCGCTGCATGGCCCTCGACACGGGGCGATGCGCATC
GCCGCATGGCCCTCGACACCGGGCGATGCGTATCGCCGCATGGCTTGCACTAGCGCCCGTAGCGTATCGCCGTGT
CGAGGGCCATGCATCGCCCGGGGCGTTGCGTATCGCCGCATGGCCCTCGACACGGGGTGATGCGTATCGCCGTGT
CGAGGGCCATGCATCGCCCGGGGCGTAGTGTATCGCCGCATGGCTTGCACTAGCGCCCGTAGCGTATCGCCGTGT
CGAGGACCATGTATCACCCGGGCGAGGATGCCCCATGCGTTGCCTTGGCAAGGCACCCCCCATGCATGTCTATGG
TTATGGTTCTCTCGGGCAACCTCAACCATGCATCACCACGCTACAGCTATTGAGGCTGCGTGGGTGATGCTACTGA
GGCTGCGTGGGCGATGCATGGCCCGGCCGGCGCGCGTCCGGGAGGCAATTTCTTGGAAACACACCGAAACGGTC
CAATTTTTTTGTGACAACTTGGCAGTGGTTAGTACTCATAAAAACACACCCAAAGAAAAAATATTGACAAAAAAAG
AGGTCCGGACAAATTTTTTTTGAAAAAAATCGTATTCGTCAATTCTGAGTATGAATACCTCATTATCCGTTTCCAAA
ACCTGTCTAGATGTGTAGTACGTTCGAAAACACCCCAAATGAAAAAAAAAAAGTGTCAAAAAACGACAACGGAGTG
AGAAGTTATGGCATTTTCAATTTCTGTGTGCCCCAATGTTCCCTTTTCAGTACCAGGGAAAAATTTTAGTGGCTATA
TTAGGGGGGAGGTGTTGGTTGGCTCCCAAAAAAAAAAAAATTTCCCCCTCGAGCTGGTGAAAGGCCAACAATGCCC
ACACGTCTCTCATGCATCCCCCAATGCATGTGAGCGTGTGGGCATGTCGCAATGAGGTCCCTTTCGAGTTTACAAC
ACCCAACAAACCACGTGGGGGCGTGGGAATGGCTCTCGATCACCCTATTGGCACGGGCGACGGATGGGCGACGG
CTGGGCGATGGCTGAGCGATGTTGGGCGTTCTCTGGGCGATACATGGGCCGGTGATTTGACGTATCGCCATAAGA
GAAATCCTTGCCATCACCCATGGCACGTCACGAGATGGCGTTGGAAATAGGGGGATGTGTTGGTTGATGGCTTCC
TGAACAAAAAAAAAAATCCCCCACGAGCTGGTGAAAGGCCGCAAAGACCCACACGTCTCCCATGCATCTAACAATG
CATGAGAGCGTGTGGGCATGTCCCGATGAGGACCCTTCGAGTTCACAACACCACGTGGGGGCGGAGGGAATGGC
TCTCGATCTCCCTATTGGCATGGGCGATCCTGGGCGACGTATGGGCGATGGATGAGTGATGTTGGGCGCTCTTTG
GGCGATACATGGGCCAGTGATTCGGCGTATCGCCATAAGAGAATTCCTTGCCATCGTCCATGGCACGTCACGAGA
TGGTGTTGGAAATAGTTGGCTGGTGGAAAAAAAAACCATTGGATGGATCGTCGTGAGAACGGGTTGGTTGGATG
GTTGGGCGCATGGGCGCAGCTTGGGAGATCCCCGGGCAATCGATGGGCATTGGATGGTTGGCGGACATCTTGTG
AACGTTTGTGATCACCATGTGGAGCATTGCTCGGGCAGGTGCTGAGCAACCCCAAATATGTGAGCTGGGTACTTTT
TGTGGACGGTTGTCAATCCTCATGCTTGGTAGCTATGAAATGAAGGGGGTTCACCACGCCTCTCCCTCGGATAACC
TTCCGGGCCCATACCACACGTTCATTTGACCTGTTTAAGCCGCTTCATGTGCGAAAAAAAACATGGTCTTCTCAACG
AGTGGCTTAGGCTTGGGGGTGTGAGGGGGGAGGAGTGTGGTGCTGCCCACGATGCATCAACTTGCCCCGATACT
ATTAGACGATCTTGTTCGTCTCCCATGCAACATCTTAGTCCGTATATCGCTCATCCTAATTACATTCGTTTGTAACGC
TACGGCGCAGGTGGCCAAAGCGGGCGATGGAATAAGGTGTTGCATGGGGGCTTTATTGGTCTCCCATGCACCATC
TTAGGCCTTTTGATCGCTCAATCCGGTTACATTCATTTGTAATGCTGCGGTGCTGGTGGCCAAAGCGAGCGGTGGA
ATAAGGTGTTGCATGGGGGAAATATAGGGCTCCCATGCAACATCTTAGGCCGTGATCGCTCAATCCGGTTACATTC
GTGTGTAACGCTGCGGTGCTGGTGGCCAAAGCGGGCGATGGAATAAGGTGTTGCATGGGGGATCATGCGTACGT
CTGCTCCCATGCACCATCTTAGGCCTTGATCGCTCAATCCGGTTACATTCGTTTGTAACGCTGCGGCGCTGGTGGCC
AAAGCGGGCGATGGAATAAGGTGTTGCATTGGGGAACATCATGGGCTGTTACTTTGATGCTTGGGGGGGTTCACT
ATGCCCCTCCCCAGGATAACCTTCCCGGCCCATTCCACACGGCCATTTGACTTGTTTGACCCGCTTCATGCGCAAAA
AACATGGTCATCTCAACGAGTGGTTTAGGATAGGGGGTGTGAGGGGGGAGGAGTGTGTTGCTGCCCACTATGCA
TCAACTTGCCCCGATGCTATCAGACGACGTGTTAGTCTCCCATGCAGCATCTTAGTCCGTATATCGCTCAATCCGGT
TACATTCGTTTGTAACGCTGCGGCGCTGGTGGCCAAAGCGGGGCGATGGAATGAGGTGTTGCATGGGGGTACTC
CAGGTCTCCCATGCAACATCTTAGTCCGTACATCGCTCAATCCGGTTACATTCGTTTGTAACGCTGCGGCGCTGGTG
GCCAAAGCGGGCGATGGAATAGGTTGTTGCATGGGGGTACGCATGGTCTCCCATGCAACATCTTAGTCTGTATAT
CGCTTATCCTGGTTACATTCGTTTGTAACGCTGCGGCGCTGGTGGCCAAAGCGGGCGATGGAATAAGGTGTTGCA
TGGTGGATCATGCGTACGTCTGCTCTCATGCACCATCTTAGGCCTTGATCGCTCAATCCGGTTACATTTGTTTGTAA
CGCTGCGGCGCTGGTGGCCAAAGCGGGCGATGGAATAAGGTGTTGCATGGGGAAAGATCATGGGGTGTTACTTT

GATGAGGGGACCTTCAGGGCATTGCTACTAGATTGCGTGTTGGGGAGACTCCCGTGCCCAATAACCAGTTTGCAC
CCATCCGGCTCCACGTAGTGCAATGACAAAATGTTCAGGAGATGCCCCGATACTGTTCTGAAGCTTTTCATGTTGG
TTCGAAACTTGCCCCGACAGTGTGTCCCGACCGTTACAGCCAGATTTCGCGCACAGCGAGTGTTTCGAACGGTGCT
TCGTTCTACCATACGTATTGCCCTTTGCGGGGTAATGCACACATATGGGTGAGCAGGCATTGTTCCCAACGCAACG
TGTTGGCGTGTGAGTGGTAGTAGAGACATCCCTGCTTTTGGGCTCCGTGCTTCGCGCATCGAACCATAAGCACATG
TTTCCCTCATTGGCGTCCGGCGTACTTGATTGTATGTCGGCAAGTAAAATGGTTCCTGTGCTCCCTACCCGAAGAAA
GTGGAAAAGATCATTGCTAACGAGATTTATTGCCTTTGGTCGGCCAAAAGCTGGCCGAGGGCAACATGTTAGTGT
GGCTTGTGAAACCAACCGCGGCGTTTGCCCGCGTGTCTGGCTAGCGAGCTTGCACGTGCTTGGGGATATGGAAAA
CATAGATGGGTCAGGGTTCTGAACTCGACCGACGCAATCGTAGCTAACGAGTGACCGCCGGACCGCAATGGTAA
GTCCCACTCCCTGCGTGACCAGGTGTAGTTGGGCGCGGCGCGGACAACCGGCGACATGAAGGAATGCTACCTGG
TTGATCCTGCCAGTAGTCATATGCTTGTCTCAAAGATTAAGCCATGCATGTGTAAGTATGAACTAATTCAGACTGTG
AAACTGCGAATGGCTCATTAAATCAGTTATAGTTTGTTTGATGGTATATGCTACTCGGATAACCGTAGTAATTCTAG
AGCTAATACGTGCAACAAACCCCGACTTCTGGAAGGGATGCATTTATTAGATAAAAGGTCAACGCGGGCTTGTCCC
GTTGCTCTGATGATTCATGATAACTCGACGGATCGCACGGCCTTTGTGCCGGCGACGCATCATTCAAATTTCTGCCC
TATCAACTTTCGATGGTAGGATAGTGGCCTACTATGGTGGTGACGGGTGACGGAGAATTAGGGTTCGATTCCGGA
GAGGGAGCCTGAGAAACGGCTACCACATCCAAGGAAGGCAGCAGGCGCGCAAATTACCCAATCCTGACACGGGG
AGGTAGTGACAATAAATAACAATACCGGGCTCTTAGAGTCTGGTAATTGGAATGAGTACAATCTAAATCCCTTAAC
GAGGATCCATTGGAGGGCAAGTCTGGTGCCAGCAGCCGCGGTAATTCCAGCTCCAATAGCGTATATTTAAGTTGT
TGCAGTTAAAAAGCTCGTAGTTGGACTTTGGGTTTGGTAGGCCGGTCCGCCCTTGTGGTGTGCACCGGTCGACCA
GTCCCTTCTACCGGCGATACGCTCCTGTCCTTAATTGGCCGGGTCGTGCCACCGGTGCTGTTACTTTGAAGAAATTA
GAGTGCTCAAAGCAAGCCCAAGCTCTGGATATATTAGCATGGGATAACATCATAGGATTTCGGTCCTATTGCGTTG
GCCTTCGGGATCGGAGTAATGATTAACAGGGACAGTCGGGGGCATTCGTATTTCATAGTCAGAGGTGAAATTCTT
GGATTTATGAAAGACGAACCACTGCGAAAGCATTTGCCAAGGATGTTTTCATTAATCAAGAACGAAAGTTGGGGG
CTCGAAGACGATCAGATACCGTCCTAGTCTCAACCATAAACGATGCCGACCAGGGATCGGCGGATGTTGCTTTTAG
GACTCCGCCGGCACCTTATGAGAAATCAAAGTTTTTGGGTTCCGGGGGGAGTATGGTCGCAAGGCTGAAACTTAA
AGGAATTGACGGAAGGGCACCACCAGGAGTGGAGCCTGCGGCTTAATTTGACTCAACACGGGGAAACTTACCAG
GTCCAGACATAGTAAGGATTGACAGACTGAGAGCTCTTTCTTGATTCTATGGGTGGTGGTGCATGGCCGTTCTTAG
TTGGTGGAGCGATTTGTCTGGTTAATTCCGTTAACGAACGAGACCTCAGCCTGCTAACTAGCTATGTGGAGGTACC
CTCCACGGCCAGCTTCTTAGAGGGACTATGGCCGTTTAGGCCACGGAAGTTTGAGGCAATAACAGGTCTGTGATG
CCCTTAGATGTTCTGGGCCGCACGCGCGCTACACTGATGTATTCAACGAGTCTATAGCCTTGGCCGACAGGCCTGG
GTAATCTTTGAAAATTTCATCGTGATGGGGATAGATCATTGCAATTGTTGGTCTTCAACGAGGAATTCCTAGTAAG
CGCGAGTCATCAGCTCGCGTTGACTACGTCCCTGCCCTTTGTACACACCGCCCGTCGCTCCTACCGATTGAATGGTC
CGGTGAAATGTTCGGATCGCGGCAACGTGGGTGGTTCGCCGCCGGCGACGCCGCGAGAAGTCCATTGAACCTTAT
CATTTAGAGGAAGGAGAAGTCGTAACAAGGTTTCCGTAGGTGAACCTGCGGAAGGATCATTGTCGATACCTGCTC
AGCAGAACGACCCGCGAACACGTGAAAACAACTTACCATGCCCGGGGAGCGGGCTCTGGCCAGCGACCCGGCGC
AGCAACAAAATTCGGCGCAACTGGCGTCAAGGAAAACTTATCGGAAGCAAGGCGTCGGCTCGTTCGGCGCCGCG
TATCCAAATACTCAAACGACTCTCGGCAATGGATATCTCGGCTCTTGCATCGATGAAGAACGTAGCGAAATGCGAT
ACTTGGTGTGAATTGCAGAATCCCGTGAACCATCGAGTCTTTGAACGCAAGTTGCGCCCGAAGCCTTTTTGGCTGA
GGGCACGCCTGCCTGGGCGTCACACACAGCGTCGCCCCCCACCAATCCCTCTGGATGGGATGGGGGCGGAGATT
GGCCCCCCGAGCCCTCCGGGGCACGGTCGGCATAAATGTGGGTCCCCGGCGACGAGCGTCGCGGTCAGCGGTGG
TTGTATACTCATCCCCCAAAGACGAAATGACGCGCACGCCTCGTCGCTCGGCGGACAGAGCAAACCCTAGGAAGC
CGGGCTTCCACCTGCGACCCCAGGTCAGGCGGGATTACCCGCTGAGTTTAAGCATATCAATAAGCGGAGGAAAAG
AAACTTACGAGGATTCCCCTAGTAACGGCGAGCGAACCGGGAACAGCCCAGCTTGGGAATCGGGCGACTCCGTC
GTCCGAATTGTAGTCTGTAGAAGCGTCCTCAGCGGCGGACCGGGCCCAAGTCCCCTGGAAGGTGGCGCCAGAGA

GGGTGAGAGCCCCGTCGTGCCCGGACCCTGTCGCACCACGAGGCGCTGTCGGCGAGTCGGGTTGTTTGGGAATG
CAGCCCCAATCGGGCGGTAAATTCCGTCCAAGGCTAAATACTGGCGAGAGACCGATAGCGAACAAGTACCGCGA
GGGAAAGATGAAAAGGACTTTGAAAAGAGAGTCAAAGAGTGCTTGAAATTGTCGGGAGGGAAGCGGATGGGG
CCGGCGATGTGCACCGGTCGGATGCGGAACGGCGACAGCTGGTCCGCCGCTCGGCTCGGTGCGCGGACCGACGC
GGATTGTGGAGGCGACCCAAGCCTGGGCCTCCTCGGAGGCCCGTGGAGATGTCGTCCCCGCGATTGTGGTGGGC
AGCACGCGCCTCACGGCGTGCCTCGGCAACTGCGTGCTCCCGGCGTCGGCCAGTGGGCTCCCCATTCGGCCCGTC
TTGAAACACGGACCAAGGAGTCTGACATGTGTGCGAGTCAGCGGGTGAGTAAACCCGCGAGGCGCAAGGAAGCT
AATTGGCGGGATCCCCCTGTGGGTTGCACCGCCGACCGACCTAGATCTTCTGTGAAGGGTTCGAGTGTGAGCATA
CCTGTCGGGACCCGAAAGATGGTGAACTATGCCTGAGCGGGGCGAAGCCAGAGGAAACTCTGGTGGAGGCCCGC
AGCGATACTGACGTGCAAATCGTTCGTCTGACTTGGGTATAGGGGCGAAAGACTAATCGAACCATCTAGTAGCTG
GTTCCCTCCGAAGTTTCCCTCAGGATAGCTGGAGCTCGCGGGCGAGTTCTATCAGGTAAAGCCAATGATTAGAGG
CATCGGGGGCGCAACGCCCTCGACCTATTCTCAAACTTTAAATAGGTAGGACGGCGCGGCTACTTCGCTGAGCCG
TGCCACGGAATCGAGTGCTCCAAGTGGGCCATTTTTGGTAAGCAGAACTGGCGATGCGGGATGAACCGGAAGCC
GGGTTACGGTGCCCAACTGCGCGCTAACCCAGAACCCACAAAGGGTGTTGGTCGATTAAGACAGCAGGACGGTG
GTCATGGAAGTCGAAATCCGCTAAGGAGTGTGTAACAACTCACCTGCCGAATCAACTAGCCCCGAAAATGGATGG
CGCTGAAGCGCGCGACCCACACCCGGCCGTCAGGGCAATTGTTAGGCCCTGATGAGTAGGAGGGCGCAGCGGTG
GCCGCGAAACCCAGGGCGCAAGCCCGGGTGGAGCCGCCGTTGGTGCAGATCTTGGTGGTAGTAGCAAATATTCA
AATGAGAACTTTGAAGGCCGAAGAGGGGAAAGGTTCCATGTGAACGGCACTTGCACATGGGTTAGTCGATCCTA
AGAGACGGCCGAAAGGCGTCAGAGAGCGTGCATCACGCGAGCTTCGAAAGGGAATCGGGTTAAAATTCCTGAAC
CGGGACACAGTGGTCGACGGCAACGTTAGGAAGTCCGGAGACGTCGGCGGGGGCCTCGGGAAGAGTTATCTTTT
CTGTTTAACAGCCTGCCCACCCTGGAAACGGCTCAGCCGGAGGTAGGGTCCAGCGGCTGGAAGAGCACCGCACG
TCGCGTGGTGTCCGGTGCGCCCCCGGCGGCCCTTGAAAATCCGGAGGACCGAATGCCAACTGTGCCCGGTCGTAC
TCATAACCGCATCAGGTCTCCAAGGTGAACAGCCTCTGGTCGATGGAACAATGTAGGCAAGGGAAGTCGGCAAAA
TGGATCCGTAACCTCGGGAAAAGGATTGGCTCTGAGGGCTGGGCACGGGGGTCCCAGTCCCGAACCCGTTGGCT
GCCGGCGGACTGCTCGAGCTGCTTCCGTGGCGAGAGCGGGTCGCCGCGTGCCGGCCGGGGGATGGACTGGGAA
CGGCTCCTTCGGGGGCCTTCCCCGGGCGTCGAACAGCCAACTCAGAACTGGTACGGACAAGGGGAATCCGACTGT
TTAATTAAAACAAAGCATTGCGATGGTCCCTGCGGATGCTAACGCAATGTGATTTCTGCCCAGTGCTCTGAATGTC
AAAGTGAAGAAATTCAACCAAGCGCGGGTAAACGGCGGGAGTAACTATGACTCTCTTAAGGTAGCCAAATGCCTC
GTCATCTAATTAGTGACGCGCATGAATGGATTAACGAGATTCCCACTGTCCCTGTCTACTATCCAGCGAAACCACA
GCCAAGGGAACGGGCTTGGCAGAATCAGCGGGGAAAGAAGACCCTGTTGAGCTTGACTCTAGTCCGACTTTGTG
AAATGACTTGAGAGGTGTAGGATAAGTGGGAGCCGACTCGTGCGGCGAAAGTGAAATACCACTACTTTTAACGTT
ATTTTACTTATTCCGTGAATCGGAAGCGGGGCACTGCTCCTCTTTTTGGATCCAAGTTCGGTCTCGACGGGACAATC
CGGGCGGAAGACATTGTCAGGTGGGGAGTTTGGCTGGGGCGGCACATCTGTTAAAAGATAACGCAGGTGTCCTA
AGATGAGCTCAACGAGAACAGAAATCTCGTGTGGAACAAAAGGGTAAAAGCTCGTTTGATTCTGATTTCCAGTAC
GAATACGAACCGTGAAAGCGTGGCCTATCGATCCTTTAGACCTTCGGAATTTGAAGCTAGAGGTGTCAGAAAAGT
TACCACAGGGATAACTGGCTTGTGGCAGCCAAGCGTTCATAGCGACGTTGCTTTTTGATCCTTCGATGTCGGCTCTT
CCTATCATTGTGAAGCAGAATTCACCAAGTGTTGGATTGTTCACCCACCAATAGGGAACGTGAGCTGGGTTTAGAC
CGTCGTGAGACAGGTTAGTTTTACCCTACTGATGACAGTGTCGCGATAGTAATTCAACCTAGTACGAGAGGAACC
GTTGATTCACACAATTGGTCATCGCGCTTGGTTGAAAAGCCAGTGGCGCGAAGCTACCGTGTGCAGGATTATGAC
TGAACGCCTCTAAGTCAGAATCCAGGCTAGAGAAGCGACGCATGCGCCCGCCGCCCAATTGCCGACCTGCAGTAG
GGGTCCTCGGACCCCCAAAGGCACGTGTCATTGGCGTAGCTCCCGTGGCCGACGAGCCGCGTGGTGCCGCCTTGA
AGTATAATTCCTACTGAGCGGCGGGCTGAATCCTTTGCAGACGACTTAAATACGCGACGGGGTATTGTAAGTGGC
AGAGTGGCCTTGCTGCCACGATCCACTGAGATTCAGCCCCATGTCGCATCGATTCGTCCCTCCCCACCCCCATCAA

>A_parviflora_LC/1-11278

ACAACAACTCACCACCAATACCCTCGTATACCCCTCGAGGTTACAGCCGCAACTTTATTCTACGTCTACGTGAAAGG
GAGGGATACTTAGAAGAAAGTCTAAGTATAGGAATTAAAAACTCCACTGATTATGCCTAGCCCGGCGCATGTCTCT
TTACACTAAGGCAAGTGCACATGCATCGTCGGGCCAAGGATGGCTATGTATCACTTGGGCAAGCGACGTTGCCCG
GGAAGGAAGCTTATGCATCGCCCGGGCAATGACGGCTCTGTATCACTCGAGGAAGGATGACTATGCATTGTGGG
GGAAAGGATGCTTATGCATCGCCGGATCAAGGATAATCGTGTATCACCCGGGCAAATATGCCCCTGGAGTCCCGA
GGGTGCTGTGAGATGGACCACCCGGGGTAATATGCCCTGACGTAGCATACCCTCCGGGCAAATATGAAACGGAG
CCCCGACGGCGCTTCTGAGATGTATCACCCCGGAATATATGCCCTGGCGTAGCATATCGCCCGGGTAAATATGCCT
CGGTAGCTCCAACGATGCTTGTGAGATGGATCACCCTGGGAAATATGCCCGAGAGTAGCATAACTCTCGGGCAAA
TATGCATCGGCAGCCCCGACGGTGCTTGTGAGATGGATCAACCCGGGGTAATATGTCCCGGGGTAGCATATCCCC
AAGCAAATACGCCCCTGGAGTCCAGAGGGTGCTGTGAGTTGGATCACCCGGGGTAATATGCCCCGGCGTAGCATA
TCACCCACGGGAATATCCACCTGGAGTCCTAACAGTGCTTGTGAGAGGGATCACCCGGGGAAATATGCCACAACA
TAGCATATCGCCCCGGCGAAAATGCCTCGGTAGCCCCGACGGTGCTTGTCAGATGAATCACCCCGGCTGATATGCC
CCGACGATGCTTGTGAGAGGGATCACCCGGATAAATATGCCCCGACATAGCATATCGTCCGGACAAAAATGCCTT
GGTAGCCCCGGTGGTGCTTATGAGATGAATCACCTGGGCGCCAAGCATCGGCCGGGCAAAAATGCCCCATGCGT
GGCCTTGGCAAGGCACGCCCGCATGGCTTGCACTAGCTCCCATAGCGTATCGTCGTGTCGAGGGCCATGCATCGC
CCGGGGCGTTGCGTATCGCCGCATGGCTTGCACTAGCGCCCGTAGCGCATCGCCGTGTCAAGGGCCATGCATCGC
CCGGGGCGATGCGTATCGCCGCATGGCCCTCGACACGGGCCGATGCGTATCGCCGCATGGCTTGCACTAGGGCCC
ATACCGTATCGCCGTGTCGAGGGCCATGCATCGCCCGGGGCGTTGCGTATCGCCGCATGGCCCTCGACACGGGGC
GATGCGGATCGCTACATGGCCCTCGACAAGGGGCGATGCGTATCGCCGCATGGCTTGCACTAGCGCCCGTAGCGT
ATCGCCGTGTCGAGGGCCATGCATCGCCCGGGGCGATGCGTATCGCCGCATGGCTTGCACTAGCGCCCGTAGCGT
ATCGCCGTGTCGAGGGCCATGCATCGCCCGGGGCGTTGCGTATCGCCGCATGGCCCTCGACACGGGGCGATGCG
GATCGCCGCATGGCCCTCGACACTTGGCGATGCGGATCGCTGCATGGCCCTCGACACGGGGCGATGCGCATCGCC
GCATGGCCCTCGACACCGGGCGATGCGTATCGCCGCATGGCTTGCACTAGCGCCCGTAGCGTATCGCCGTGTCGA
GGGCCATGCATCGCCCGGGGCGTTGCGTATCGCCGCATGGCCCTCGACACGGGGTGATGCGTATCGCCGTGTCGA
GGGCCATGCATCGCCCGGGGCGTAGTGTATCGCCGCATGGCTTGCACTAGCGCCCGTAGCGTATCGCCGTGTCGA
GGACCATGTATCACCCGGGCGAGGATGCCCCATGCGTTGCCTTGGCAAGGCACCCCCCATGCATGTCTATGGTTAT
GGTTCTCTCGGGCAACCTCAACCATGCATCACCACGCTACAGCTATTGAGGCTGCGTGGGTGATGCTACTGAGGCT
GCGTGGGCGATGCATGGCCCGGCCGGCGCGCGTCCGGGAGGCAATTTCTTGGAAACACACCGAAACGGTCCAAT
TTTTTTGTGACAACTTGGCAGTGGTTAGTACTCATAAAAACACACCCAAAGAAAAAATATTGACAAAAAAAGAGGT
CCGGACAAATTTTTTTTGAAAAAAATCGTATTCGTCAATTCTGAGTATGAATACCTCATTATCCGTTTCCAAAACCTG
TCTAGATGTGTAGTACGTTCGAAAACACCCCAAATGAAAAAAAAAAGTGTCAAAAAACGACAACGGAGTGAGAA
GTTATGGCATTTTCAATTTCTGTGTGCCCCAATGTTCCCTTTTCAGTACCAGGGAAAAATTTTAGTGGCTATATTAG
GGGGGAGGTGTTGGTTGGCTCCCAAAAAAAAAAAATTTCCCCCTCGAGCTGGTGAAAGGCCAACAATGCCCACAC
GTCTCTCATGCATCCCCCAATGCATGTGAGCGTGTGGGCATGTCGCAATGAGGTCCCTTTCGAGTTTACAACACCG
AACAAACCACGTGGGGGCGTGGGAATGGCTCTCGATCACCCTATTGGCACGGGCGACGGCTGGGCGACGGATGG
GCGACGGCTGGGCGATGTTGGGCGTTCTCTGGGCGATACATGGGCCGGTGATTTGACGTATCGCCATAAGAGAA
ATCCTTGCCATCATCCATGGCACGTCACGAGATGGCGTTGGAAATAGGGGGGATGCGTTGGTTGATGGCTTCCTG
AACAAAAAAAATCCCCCATGAGCTGGTGAAAGGCCGCAAAGACCCACACGTCTCCCATGCATCTAACAATCCATGA
GAGCGTGTGGGCATGTCCCGATGAGGTCCCTTCGAGTTCACAACACCACGTGGGGGCGGAGGGAATGGTTCTCG
ATCATCCTATTGGCATGGGTGATCCTGGGCGACGTATGGGCGATGGCTGAGCGATGTTGGGCGCTCTTTGGGCGA
TACATGGGCCAGTGATTCGGCGTATCACCATAAGAGAATTCCTTGCCATCGTCCATGGCACGTCACGAGATGGTGT
TGGAAATAGTTGGCTGGTGGAAAAAAAAAACCCATTGGATGGACCGTCGTGAGAACGGGTTGGTTGGATGGTTGG
GCGCATGGGCGCAGCTTGGGAGATCCCCGGGCAATCGATGGGCATTGGATGGGTTGGCGGACATCTTGTGAACG
TTTGTGATCACCATATGGAGCGTTGCTCGGGCAGGTGCTGAGCAACCCCAAATATGTGAGCTGGGTGCTTTTTGTG

GACGGTTGTCAATCCTCATGCTTGGTAGCCATGAAATGAATGGGGTTCACCACGCCTCTCCCTTGGATAACCTTCC
GAGCCCATACCACACGATCATTTGACCTGTTTAAGCCCTTCATGTGCGAAAAAAAACATGGTCTTCTCAACGAGTG
GCTTAGGCTTGGGGGTGTGAGGGGGGAGGAGTGTGGTGCTGCCCACGATGCATCAACTTGCCCCAATACTATTAG
ACGATCGTTTTCGTCTCCCATGTAACATCTTAGTCCGTATATCGCTCATCCTAATTACATTCGTTTGTAACGCTACGG
CGCAGGTGGCCAAAGCGGGCGATGGAACAAGGTGTTGCATGGGGGCTTTATTGGTCTCCCATGCACCATCTTAGG
CCTTTTGATCGCTCAATCCGGTTACATTCATTTGTAACGCTGCGGCGCTGGTGGCCAAAGTGAGCGGTGGAATAAG
GTGTTGCATGGGGGAAATATAGGGCTCCCATGCAACATCTTAGGCCGTGATCGCTCAATCCGGTTACATTCGTGTG
TAACGCTGCGGTGCTGGTGGCCAAAGCGGGCGATGGAATAAGGTGTTGCATGGGGGATCATGCGTACGTCTGCT
CCCATGCACCATCTTAGGCCTTGATCGCTCAATCCGGTTACATTCGTTTGTAACGCTGCGGCGCTGGTGGCCAAAG
CGGGCGATGGAATAAGGTGTTGCATTGGGGAACATCATGGGCTGTTACTTTGATGCTTGGGGGGGTTCACTATGC
CCCTCCCCAGGATAACCTTCCCGGCCCATTCCACACGGCCATTTGACTTGTTTGACCCGCTTCATGCGCAAAAAACA
TGGTCATCTCAACGAGTGGTTTAGGATAGGGGGTGTGAGGGGGGAGGAGTGTGTTGCTGCCCACTATGCATCAA
CTTGCCCCGATGCTATCAGACGACGTGTTAGTCTCCCATGCAGCATCTTAGTCCGTATATCGCTCAATCCGGTTACA
TTCGTTTGTAACGCTGCGGCGCTGGTGGCCAAAGCGGGGCGATGGAATGAGGTGTTGCATGGGGGTACTCCAGG
TCTCCCATGCAACATCTTAGTCCGTACATCGCTCAATCCGGTTACATTCGTTTGTAACGCTGCGGCGCTGGTGGCCA
AAGCGGGCGATGGAATAGGTTGTTGCATGGGGGTACGCATGGTCTCCCATGCAACATCTTAGTCTGTATATCGCTT
ATCCTGGTTACATTCGTTTGTAACGCTGCGGCGCTGGTGGCCAAAGCGGGCGATGGAATAAGGTGTTGCATGGTG
GATCATGCGTACGTCTGCTCTCATGCACCATCTTAGGCCTTGGTCGCTCAATCCGGTTACATTTGTTTGTAACGCTG
CGGCGCTGGTGGCCAAAGCGGGCGATGGAATAAGGTTTTGCATGGGGGAAAATCATGGGGTGTTACTTTGATGC
TGGGACCTTCAGGGCATTGCTACTAGATTGCGTGTTGGGGAGACTCCCGTGCCCAATAACCAGTTTGCACCCATCC
CCATCCGGCTCCACGTAGTGCAATGACAAAATGTTCACGAGATGCCCCGATATTGTTCTGAAGCTTTTCATGTTGGT
TCGAAACTTGCCCCGACAGTGTGTCCCGACCGTTACAACCAGGTTTCGCGCACAGCGAGCGTTTTGAACGGCGCTT
CGTTCTACCATACGTATTGCCCTATTGCGGGGTGATGCACACATATGGGTGAGCAGGCATTGTTCCCAACGCAACG
TGTTGGCGTGTGAGTGGTAGTAGAGACATCCCTGCTTTTGGGCTCCGTGCTTCGCGCATCGAACCATAAGCACATG
TTTCCCTCATTGGCGTCCGGCGTACTTGATTGTATGTCGGCAAGTAAAATGGTTCCTGTGCTCCCTACCCGAAGAAA
GTGGAAAAGATCATTGCTAACGAGATTTATTGCCTTTGGTCGGCCAAAAGCTGGCCGAGGGCAACATGTTAGTGT
GGCTTGTGAAACCAACCGCGGCGTTTGCCCGCGTGTCTGGCTAGCGAGCTTGCACGTGCTTGGGGATATGGAAAA
CATAGATGGGTCAGGGTTCTGAACTCGACCGACGCAATCGTAGCTAACGAGTGACCGCCGGACCGCAATGGTAA
GTCCCACTCCCTGCGTGACCAGGTGTAGTTGGGCGCGGCGCGGACAACCGGCGACATGAAGGAATGCTACCTGG
TTGATCCTGCCAGTAGTCATATGCTTGTCTCAAAGATTAAGCCATGCATGTGTAAGTATGAACTAATTCAGACTGTG
AAACTGCGAATGGCTCATTAAATCAGTTATAGTTTGTTTGATGGTATATGCTACTCGGATAACCGTAGTAATTCTAG
AGCTAATACGTGCAACAAACCCCGACTTCTGGAAGGGATGCATTTATTAGATAAAAGGTCAACGCGGGCTTGTCCC
GTTGCTCTGATGATTCATGATAACTCGACGGATCGCACGGCCTTTGTGCCGGCGACGCATCATTCAAATTTCTGCCC
TATCAACTTTCGATGGTAGGATAGTGGCCTACTATGGTGGTGACGGGTGACGGAGAATTAGGGTTCGATTCCGGA
GAGGGAGCCTGAGAAACGGCTACCACATCCAAGGAAGGCAGCAGGCGCGCAAATTACCCAATCCTGACACGGGG
AGGTAGTGACAATAAATAACAATACCGGGCTCTTAGAGTCTGGTAATTGGAATGAGTACAATCTAAATCCCTTAAC
GAGGATCCATTGGAGGGCAAGTCTGGTGCCAGCAGCCGCGGTAATTCCAGCTCCAATAGCGTATATTTAAGTTGT
TGCAGTTAAAAAGCTCGTAGTTGGACTTTGGGTTTGGTAGGCCGGTCCGCCCTTGTGGTGTGCACCGGTCGACCA
GTCCCTTCTACCGGCGATACGCTCCTGTCCTTAATTGGCCGGGTCGTGCCACCGGTGCTGTTACTTTGAAGAAATTA
GAGTGCTCAAAGCAAGCCCAAGCTCTGGATATATTAGCATGGGATAACATCATAGGATTTCGGTCCTATTGCGTTG
GCCTTCGGGATCGGAGTAATGATTAACAGGGACAGTCGGGGGCATTCGTATTTCATAGTCAGAGGTGAAATTCTT
GGATTTATGAAAGACGAACCACTGCGAAAGCATTTGCCAAGGATGTTTTCATTAATCAAGAACGAAAGTTGGGGG
CTCGAAGACGATCAGATACCGTCCTAGTCTCAACCATAAACGATGCCGACCAGGGATCGGCGGATGTTGCTTTTAG
GACTCCGCCGGCACCTTATGAGAAATCAAAGTTTTTGGGTTCCGGGGGGAGTATGGTCGCAAGGCTGAAACTTAA

AGGAATTGACGGAAGGGCACCACCAGGAGTGGAGCCTGCGGCTTAATTTGACTCAACACGGGGAAACTTACCAG
GTCCAGACATAGTAAGGATTGACAGACTGAGAGCTCTTTCTTGATTCTATGGGTGGTGGTGCATGGCCGTTCTTAG
TTGGTGGAGCGATTTGTCTGGTTAATTCCGTTAACGAACGAGACCTCAGCCTGCTAACTAGCTATGTGGAGGTACC
CTCCACGGCCAGCTTCTTAGAGGGACTATGGCCGTTTAGGCCACGGAAGTTTGAGGCAATAACAGGTCTGTGATG
CCCTTAGATGTTCTGGGCCGCACGCGCGCTACACTGATGTATTCAACGAGTCTATAGCCTTGGCCGACAGGCCTGG
GTAATCTTTGAAAATTTCATCGTGATGGGGATAGATCATTGCAATTGTTGGTCTTCAACGAGGAATTCCTAGTAAG
CGCGAGTCATCAGCTCGCGTTGACTACGTCCCTGCCCTTTGTACACACCGCCCGTCGCTCCTACCGATTGAATGGTC
CGGTGAAATGTTCGGATCGCGGCAACGTGGGTGGTTCGCCGCCGGCGACGCCGCGAGAAGTCCATTGAACCTTAT
CATTTAGAGGAAGGAGAAGTCGTAACAAGGTTTCCGTAGGTGAACCTGCGGAAGGATCATTGTCGATACCTGCTC
AGCAGAACGACCCGCGAACACGTGAAAACAACTTACCATGCCCGGGGAGCGGGCTCTGGCCAGCGACCCGGCGC
AGCAACAAAATTCGGCGCAACTGGCGTCAAGGAAAACTTATCGGAAGCAAGGCGTCGGCTCGTTCGGCGCCGCG
TATCCAAATACTCAAACGACTCTCGGCAATGGATATCTCGGCTCTTGCATCGATGAAGAACGTAGCGAAATGCGAT
ACTTGGTGTGAATTGCAGAATCCCGTGAACCATCGAGTCTTTGAACGCAAGTTGCGCCCGAAGCCTTTTTGGCTGA
GGGCACGCCTGCCTGGGCGTCACACACAGCGTCGCCCCCCACCAATCCCTCTGGATGGGATGGGGGCGGAGATT
GGCCCCCCGAGCCCTCCGGGGCACGGTCGGCATAAATGTGGGTCCCCGGCGACGAGCGTCGCGGTCAGCGGTGG
TTGTATACTCATCCCCCAAAGACGAAATGACGCGCACGCCTCGTCGCTCGGCGGACAGAGCAAACCCTAGGAAGC
CGGGCTTCCACCTGCGACCCCAGGTCAGGCGGGATTACCCGCTGAGTTTAAGCATATCAATAAGCGGAGGAAAAG
AAACTTACGAGGATTCCCCTAGTAACGGCGAGCGAACCGGGAACAGCCCAGCTTGGGAATCGGGCGACTCCGTC
GTCCGAATTGTAGTCTGTAGAAGCGTCCTCAGCGGCGGACCGGGCCCAAGTCCCCTGGAAGGTGGCGCCAGAGA
GGGTGAGAGCCCCGTCGTGCCCGGACCCTGTCGCACCACGAGGCGCTGTCGGCGAGTCGGGTTGTTTGGGAATG
CAGCCCCAATCGGGCGGTAAATTCCGTCCAAGGCTAAATACTGGCGAGAGACCGATAGCGAACAAGTACCGCGA
GGGAAAGATGAAAAGGACTTTGAAAAGAGAGTCAAAGAGTGCTTGAAATTGTCGGGAGGGAAGCGGATGGGGG
CCGGCGATGTGCACCGGTCGGATGCGGAACGGCGACAGCTGGTCCGCCGCTCGGCTCGGTGCGCGGACCGACGC
GGATTGTGGAGGCGACCCAAGCCTGGGCCTCCTCGGAGGCCCGTGGAGATGTCGTCCCCGCGATTGTGGTGGGC
AGCACGCGCCTCACGGCGTGCCTCGGCAACTGCGTGCTCCCGGCGTCGGCCAGTGGGCTCCCCATTCGGCCCGTC
TTGAAACACGGACCAAGGAGTCTGACATGTGTGCGAGTCAGCGGGTGAGTAAACCCGCGAGGCGCAAGGAAGCT
AATTGGCGGGATCCCCCTGTGGGTTGCACCGCCGACCGACCTAGATCTTCTGTGAAGGGTTCGAGTGTGAGCATA
CCTGTCGGGACCCGAAAGATGGTGAACTATGCCTGAGCGGGGCGAAGCCAGAGGAAACTCTGGTGGAGGCCCGC
AGCGATACTGACGTGCAAATCGTTCGTCTGACTTGGGTATAGGGGCGAAAGACTAATCGAACCATCTAGTAGCTG
GTTCCCTCCGAAGTTTCCCTCAGGATAGCTGGAGCTCGCGGGCGAGTTCTATCAGGTAAAGCCAATGATTAGAGG
CATCGGGGGCGCAACGCCCTCGACCTATTCTCAAACTTTAAATAGGTAGGACGGCGCGGCTACTTCGCTGAGCCG
TGCCACGGAATCGAGTGCTCCAAGTGGGCCATTTTTGGTAAGCAGAACTGGCGATGCGGGATGAACCGGAAGCC
GGGTTACGGTGCCCAACTGCGCGCTAACCCAGAACCCACAAAGGGTGTTGGTCGATTAAGACAGCAGGACGGTG
GTCATGGAAGTCGAAATCCGCTAAGGAGTGTGTAACAACTCACCTGCCGAATCAACTAGCCCCGAAAATGGATGG
CGCTGAAGCGCGCGACCCACACCCGGCCGTCAGGGCAATTGTTAGGCCCTGATGAGTAGGAGGGCGCAGCGGTG
GCCGCGAAACCCAGGGCGCAAGCCCGGGTGGAGCCGCCGTTGGTGCAGATCTTGGTGGTAGTAGCAAATATTCA
AATGAGAACTTTGAAGGCCGAAGAGGGGAAAGGTTCCATGTGAACGGCACTTGCACATGGGTTAGTCGATCCTA
AGAGACGGCCGAAAGGCGTCAGAGAGCGTGCATCACGCGAGCTTCGAAAGGGAATCGGGTTAAAATTCCTGAAC
CGGGACACAGTGGTCGACGGCAACGTTAGGAAGTCCGGAGACGTCGGCGGGGGCCTCGGGAAGAGTTATCTTTT
CTGTTTAACAGCCTGCCCACCCTGGAAACGGCTCAGCCGGAGGTAGGGTCCAGCGGCTGGAAGAGCACCGCACG
TCGCGTGGTGTCCGGTGCGCCCCCGGCGGCCCTTGAAAATCCGGAGGACCGAATGCCAACTGTGCCCGGTCGTAC
TCATAACCGCATCAGGTCTCCAAGGTGAACAGCCTCTGGTCGATGGAACAATGTAGGCAAGGGAAGTCGGCAAAA
TGGATCCGTAACCTCGGGAAAAGGATTGGCTCTGAGGGCTGGGCACGGGGGTCCCAGTCCCGAACCCGTTGGCT
GCCGGCGGACTGCTCGAGCTGCTTCCGTGGCGAGAGCGGGTCGCCGCGTGCCGGCCGGGGGATGGACTGGGAA

CGGCTCCTTCGGGGGCCTTCCCCGGGCGTCGAACAGCCAACTCAGAACTGGTACGGACAAGGGGAATCCGACTGT
TTAATTAAAACAAAGCATTGCGATGGTCCCTGCGGATGCTAACGCAATGTGATTTCTGCCCAGTGCTCTGAATGTC
AAAGTGAAGAAATTCAACCAAGCGCGGGTAAACGGCGGGAGTAACTATGACTCTCTTAAGGTAGCCAAATGCCTC
GTCATCTAATTAGTGACGCGCATGAATGGATTAACGAGATTCCCACTGTCCCTGTCTACTATCCAGCGAAACCACA
GCCAAGGGAACGGGCTTGGCAGAATCAGCGGGGAAAGAAGACCCTGTTGAGCTTGACTCTAGTCCGACTTTGTG
AAATGACTTGAGAGGTGTAGGATAAGTGGGAGCCGACTCGTGCGGCGAAAGTGAAATACCACTACTTTTAACGTT
ATTTTACTTATTCCGTGAATCGGAAGCGGGGCACTGCTCCTCTTTTTGGATCCAAGTTCGGTCTCGACGGGACAATC
CGGGCGGAAGACATTGTCAGGTGGGGAGTTTGGCTGGGGCGGCACATCTGTTAAAAGATAACGCAGGTGTCCTA
AGATGAGCTCAACGAGAACAGAAATCTCGTGTGGAACAAAAGGGTAAAAGCTCGTTTGATTCTGATTTCCAGTAC
GAATACGAACCGTGAAAGCGTGGCCTATCGATCCTTTAGACCTTCGGAATTTGAAGCTAGAGGTGTCAGAAAAGT
TACCACAGGGATAACTGGCTTGTGGCAGCCAAGCGTTCATAGCGACGTTGCTTTTTGATCCTTCGATGTCGGCTCTT
CCTATCATTGTGAAGCAGAATTCACCAAGTGTTGGATTGTTCACCCACCAATAGGGAACGTGAGCTGGGTTTAGAC
CGTCGTGAGACAGGTTAGTTTTACCCTACTGATGACAGTGTCGCGATAGTAATTCAACCTAGTACGAGAGGAACC
GTTGATTCACACAATTGGTCATCGCGCTTGGTTGAAAAGCCAGTGGCGCGAAGCTACCGTGTGCAGGATTATGAC
TGAACGCCTCTAAGTCAGAATCCAGGCTAGAGAAGCGACGCATGCGCCCGCCGCCCAATTGCCGACCTGCAGTAG
GGGTCCTCGGACCCCCAAAGGCACGTGTCATTGGCGTAGCTCCCGTGGCCGACGAGCCGCGTGGTGCCGCCTTGA
AGTATAATTCCTACTGAGCGGCGGGCTGAATCCTTTGCAGACGACTTAAATACGCGACGGGGTATTGTAAGTGGC
AGAGTGGCCTTGCTGCCACGATCCACTGAGATTCAGCCCCATGTCGCATCGATTCGTCCCTCCCCATCCCCATCCAA
AAA
