# Supplementary figures and images for: Structure and Methylation of 35S rDNA in Allopolyploids Anemone multifida (2n = 4x = 32, BBDD) and Anemone baldensis (2n = 6x = 48, AABBDD) and Their Parental Species Show Evidence of Nucleolar Dominance
Source: Front Plant Sci. 2022 Jul 6;13:908218. doi: 10.3389/fpls.2022.908218 (PMC9296772; doi:10.3389/fpls.2022.908218)

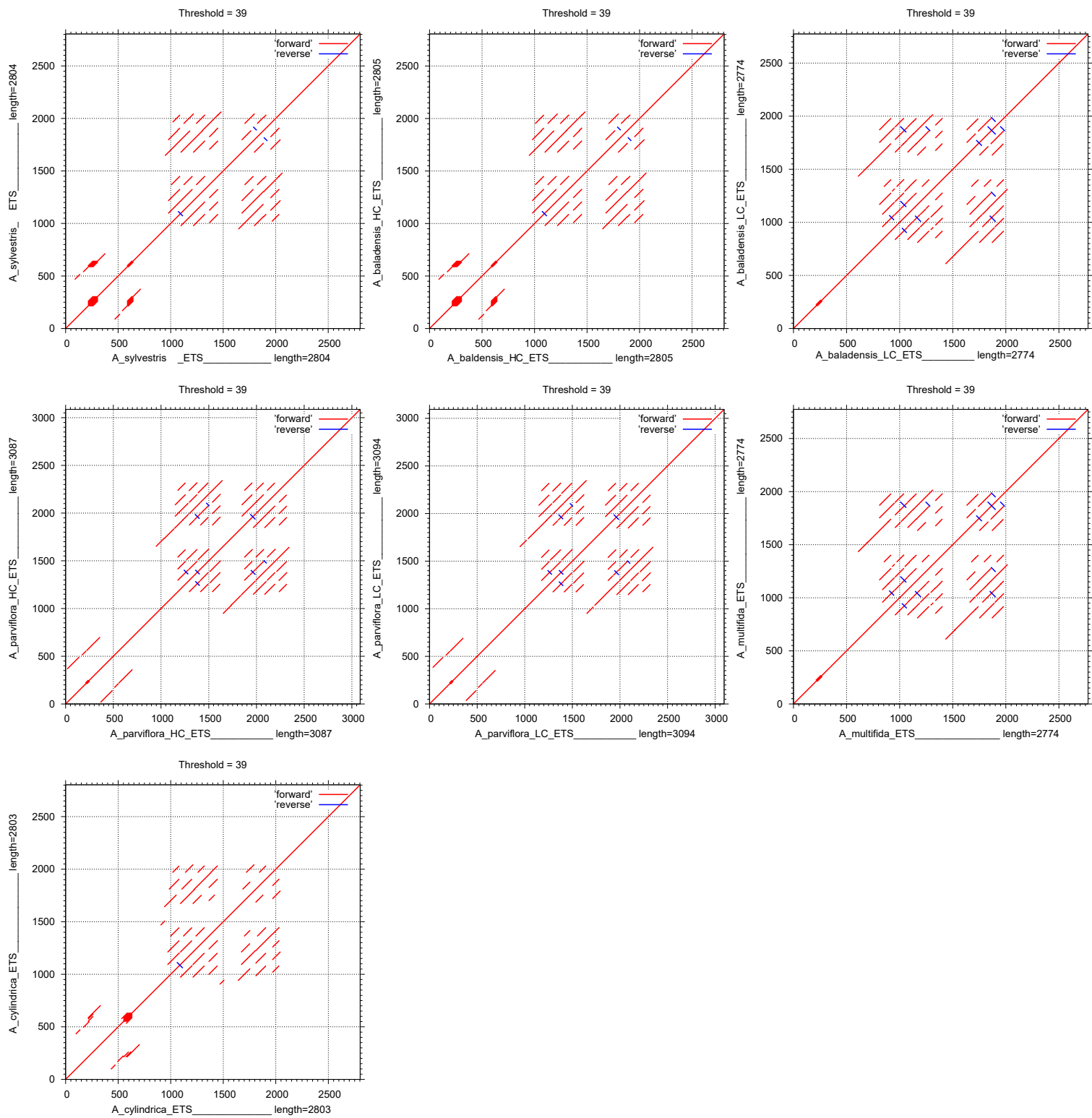

Supplementary Figure S4. Dot matrix plot of ETS region in five *Anemone* species.

Supplement: Supplementary file 4 [file Image_4.pdf]
