# Supplementary material for: Structure and Methylation of 35S rDNA in Allopolyploids Anemone multifida (2n = 4x = 32, BBDD) and Anemone baldensis (2n = 6x = 48, AABBDD) and Their Parental Species Show Evidence of Nucleolar Dominance
Source: Front Plant Sci. 2022 Jul 6;13:908218. doi: 10.3389/fpls.2022.908218 (PMC9296772; doi:10.3389/fpls.2022.908218)
Supplement: Supplementary file 5 [file Image_5.pdf]

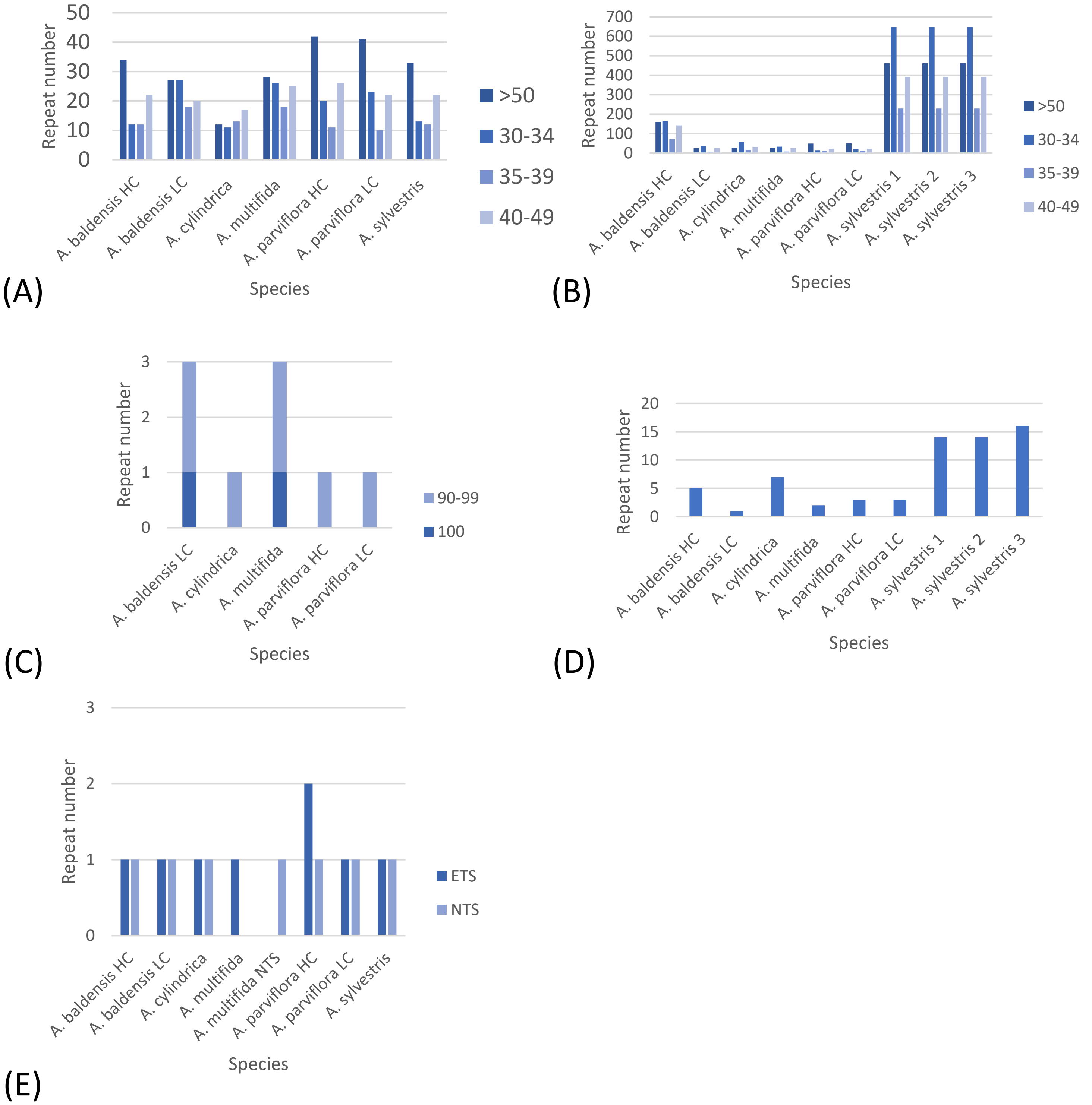

Supplementary Figure S5. Analysis of repetitive sequences in the IGS region of *Anemone*. **(A)** Distribution of forward repeats in the ETS region, **(B)** Distribution of forward repeats in the NTS region, **(C)** Frequency of tandem repeats in the ETS region, **(D)** Frequency of tandem repeats in the NTS region, **(E)** Frequency of SSRs in IGS.
