# Supplementary material for: Structure and Methylation of 35S rDNA in Allopolyploids Anemone multifida (2n = 4x = 32, BBDD) and Anemone baldensis (2n = 6x = 48, AABBDD) and Their Parental Species Show Evidence of Nucleolar Dominance
Source: Front Plant Sci. 2022 Jul 6;13:908218. doi: 10.3389/fpls.2022.908218 (PMC9296772; doi:10.3389/fpls.2022.908218)

# *A. parviflora* CpG1 island

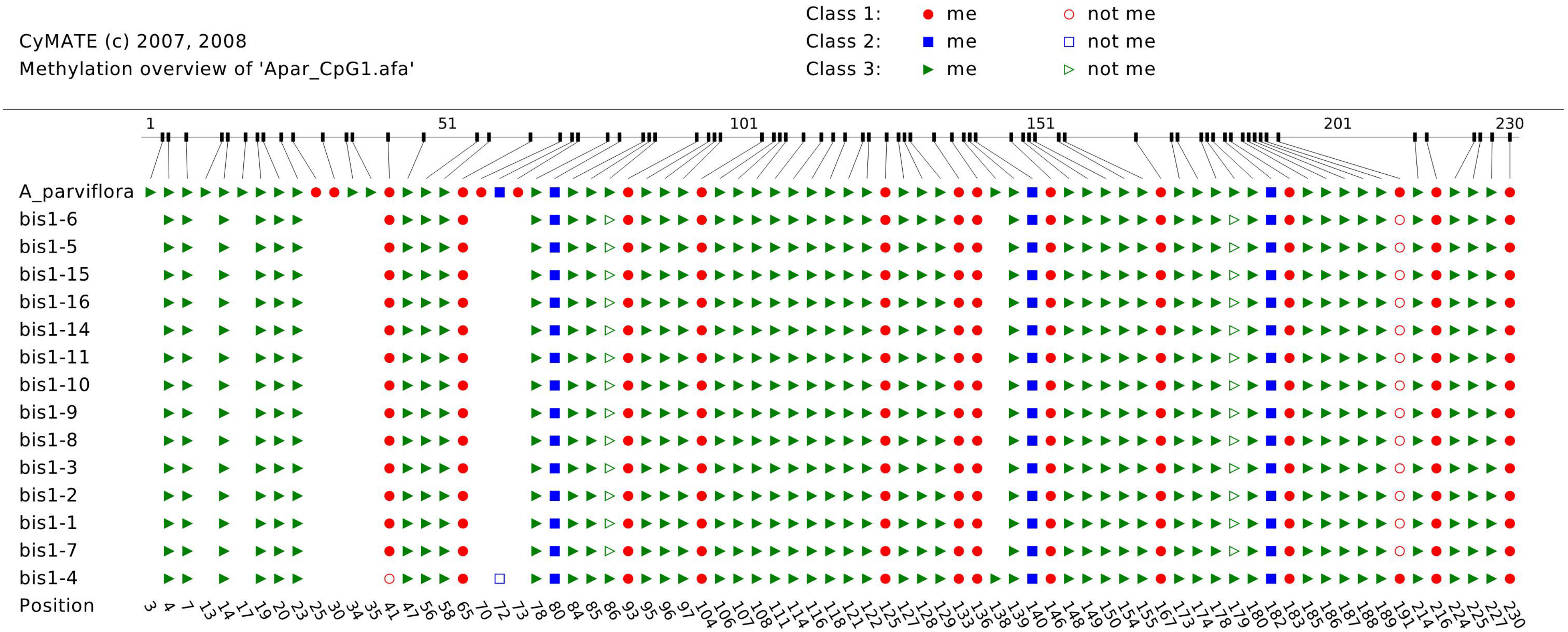


Supplementary Figure S6. Bisulfite analysis of the 35S rDNA. CyMATE program outputs from sequencing of CpG islands within ETS are shown. Red circles represent CG sites, blue squares represent CHG sites and green triangles represent CHH sites. Filled symbols – methylated Cs; empty symbols non-methylated Cs. The numbers below the diagrams indicate C residues in the alignments. Gaps in matrices were caused by sequence polymorphisms.

# *A. parviflora* CpG2 island

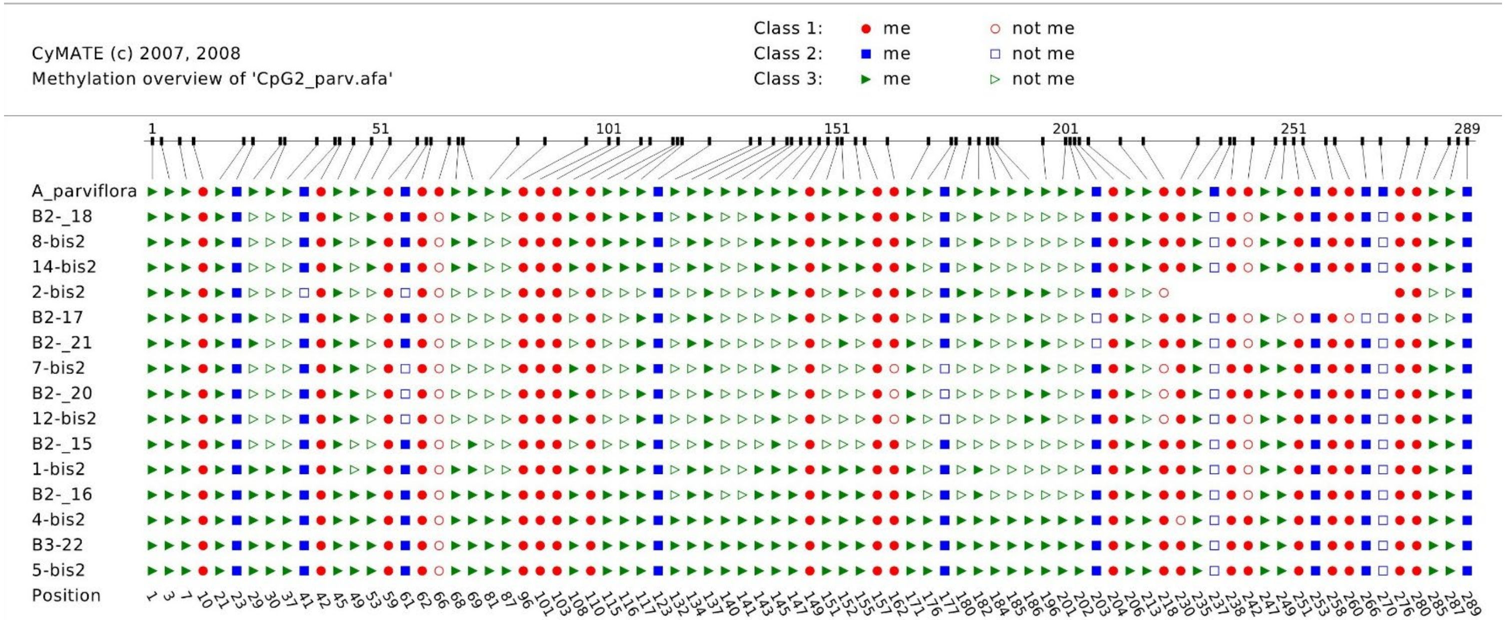


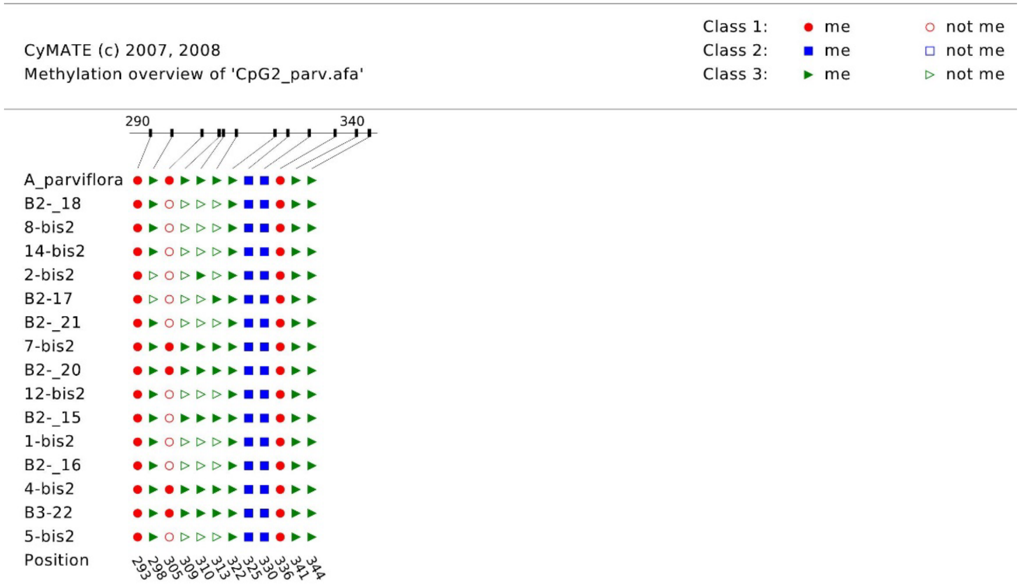

# *A. parviflora* CpG3 island

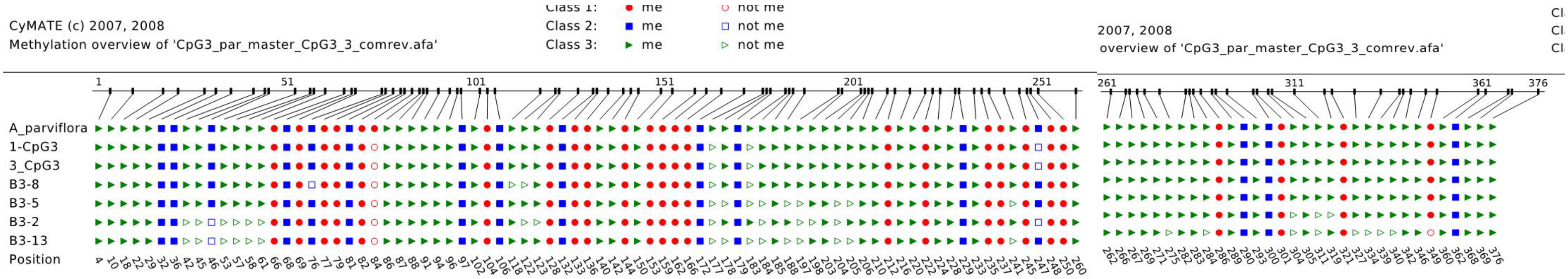


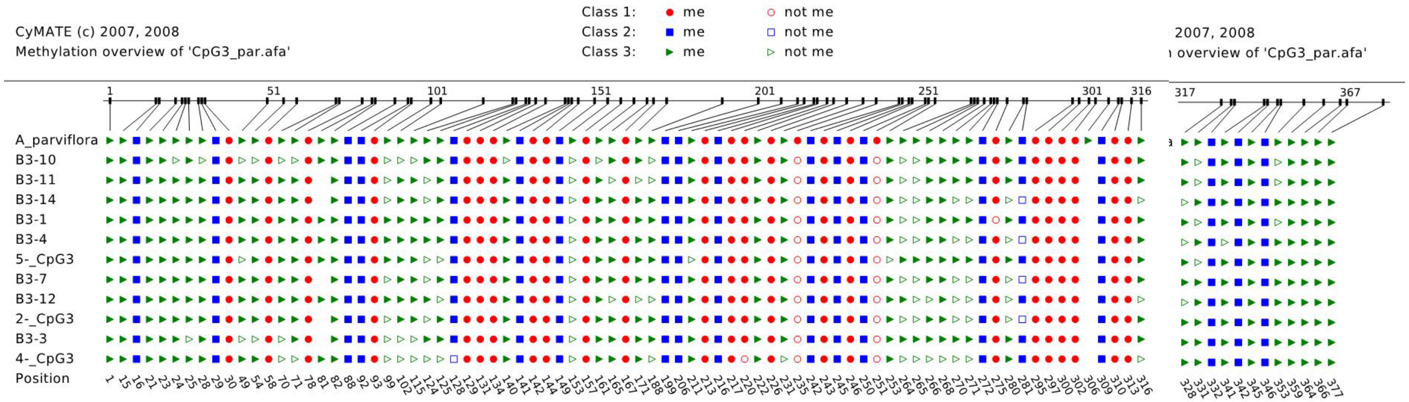

# *A. cylindrica* CpG1 island

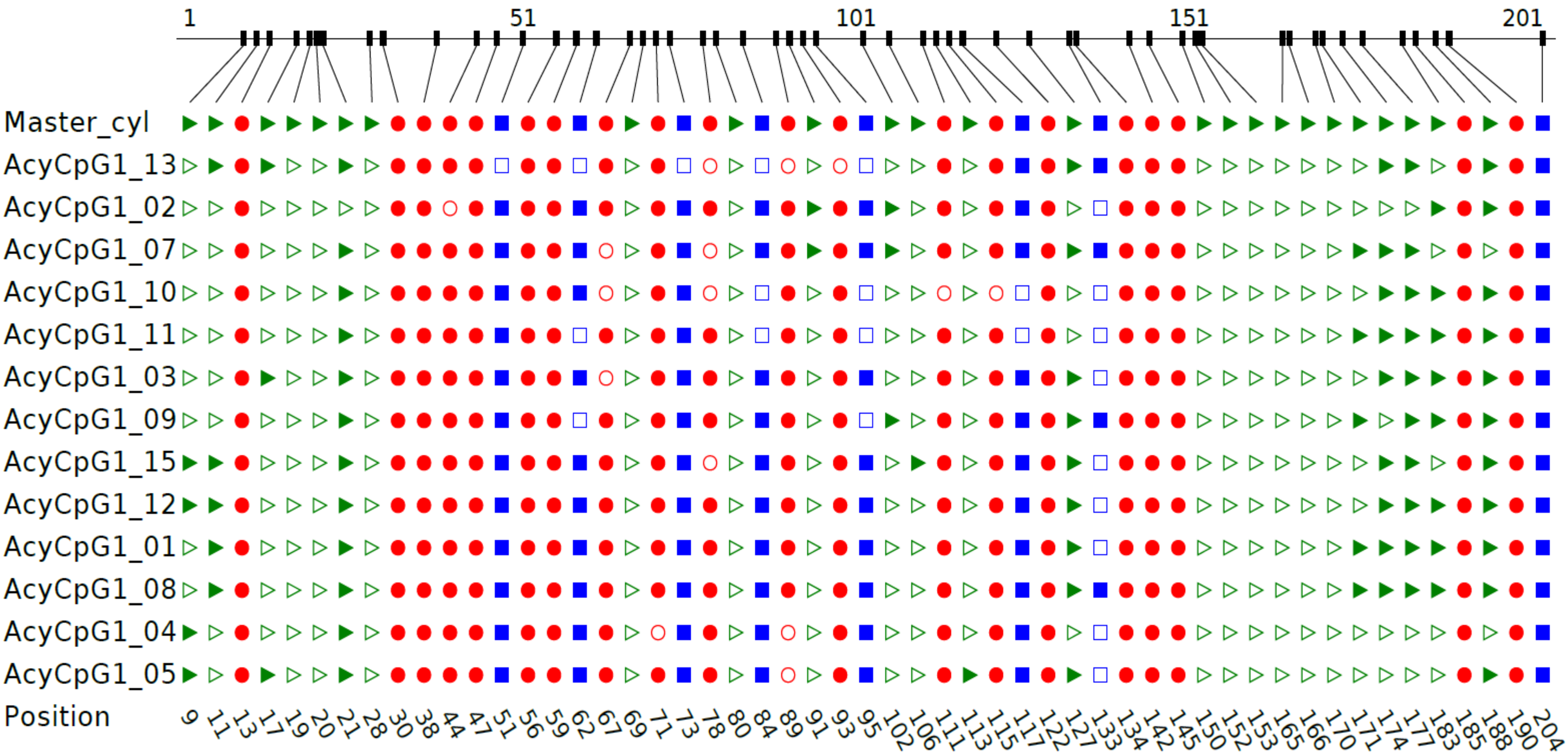

# *A. cylindrica* CpG3 island

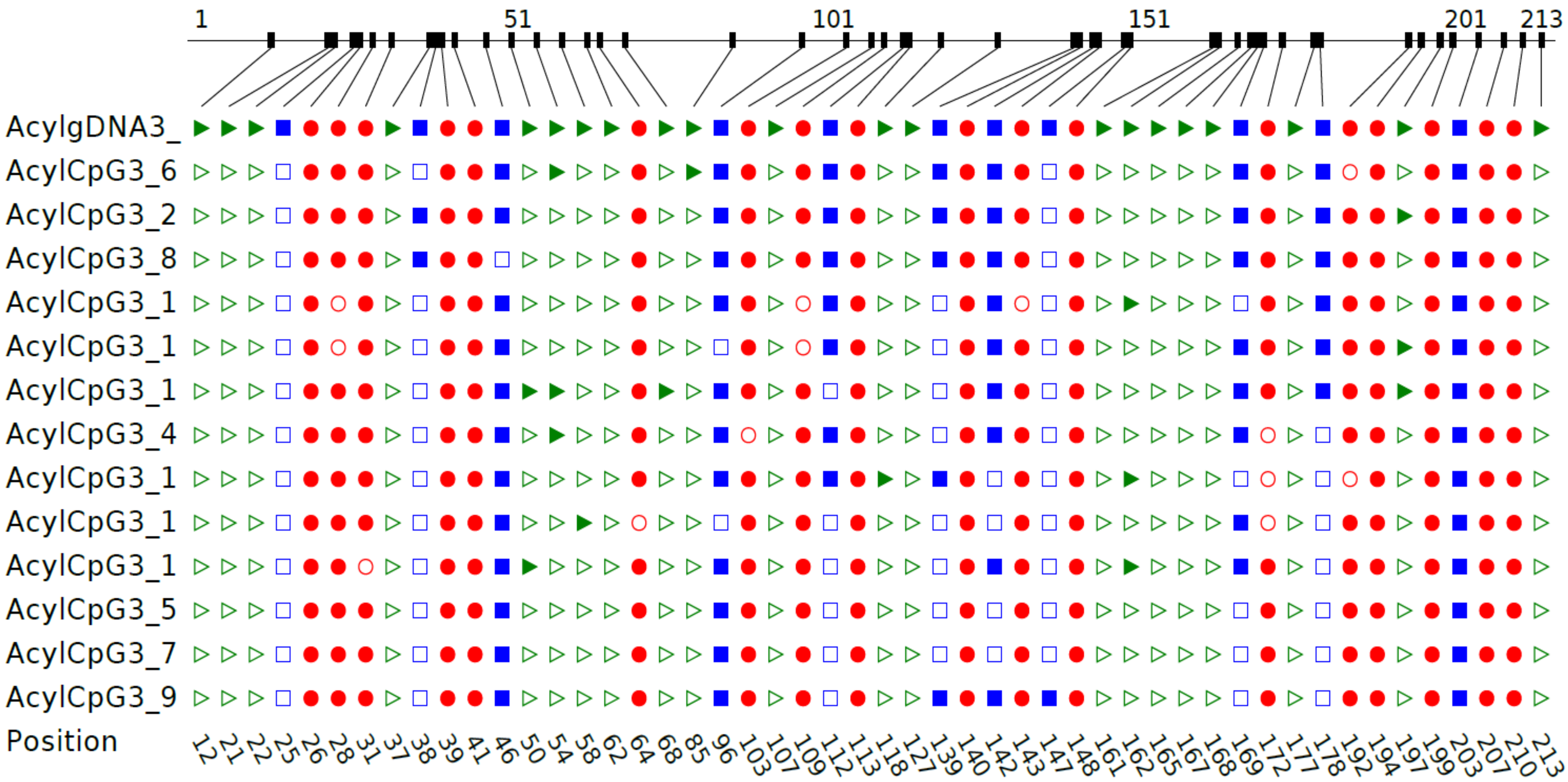

## *A. sylvestris* CpG1 island_shorter repeats

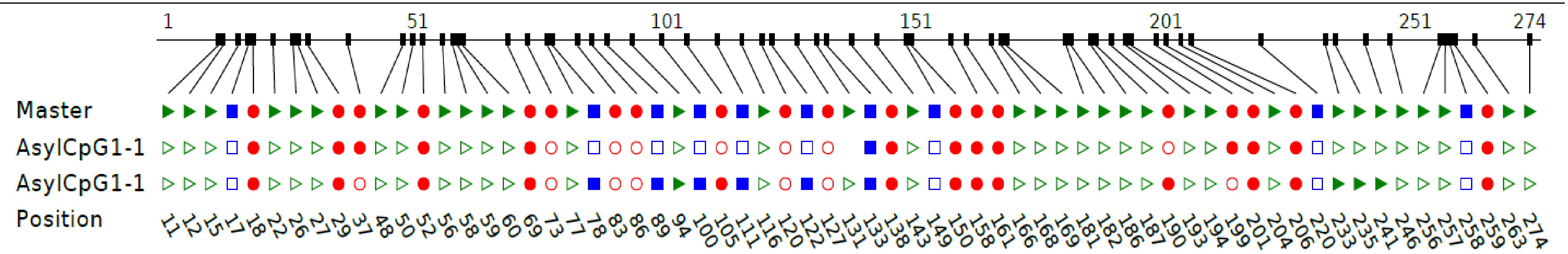


## *A. sylvestris* CpG1 island_longer repeats

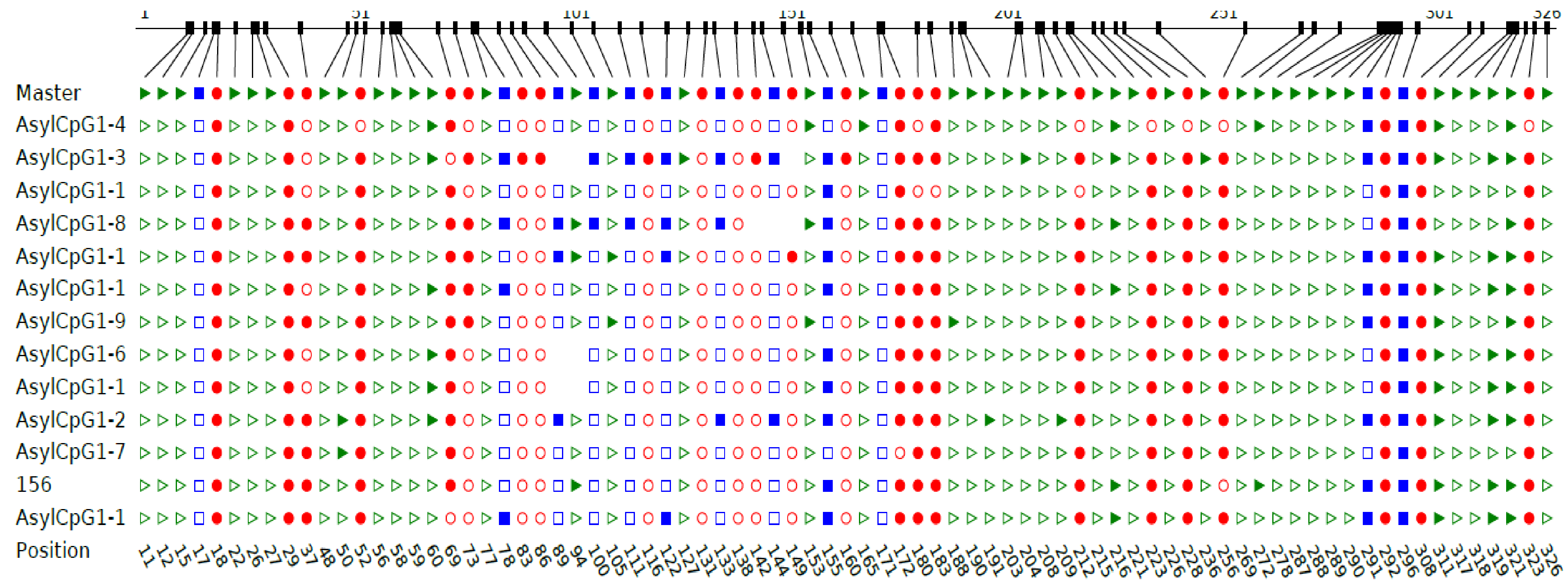

# *A. sylvestris* CpG2 island

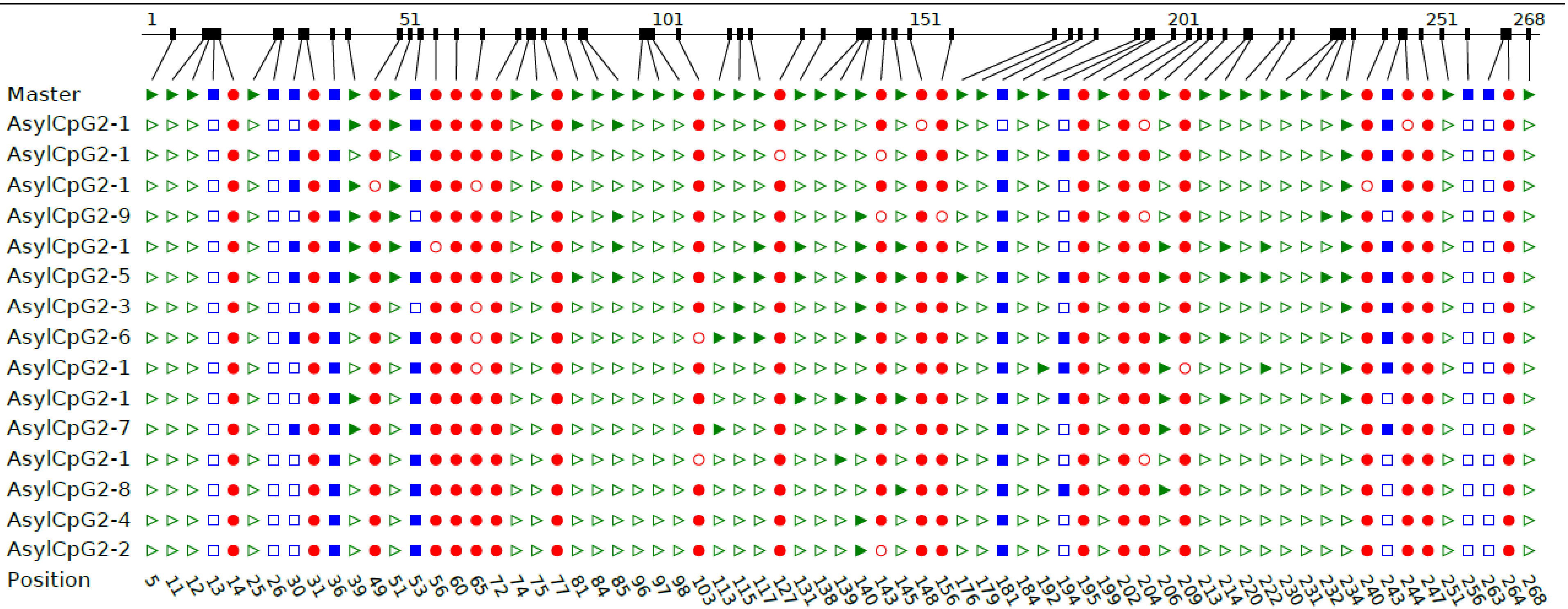

# *A. sylvestris* CpG3 island

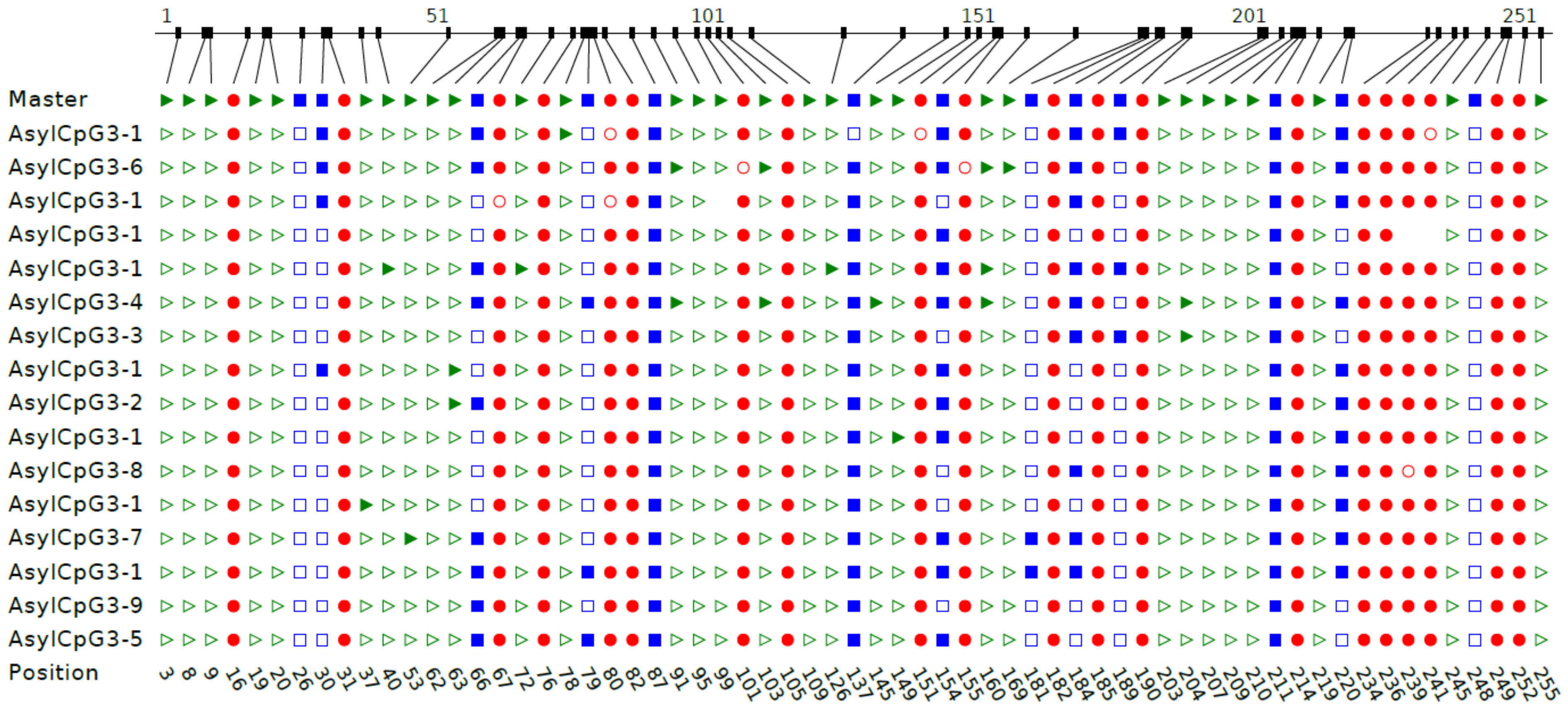

# *A. baldensis* CpG1 island_A subgenome

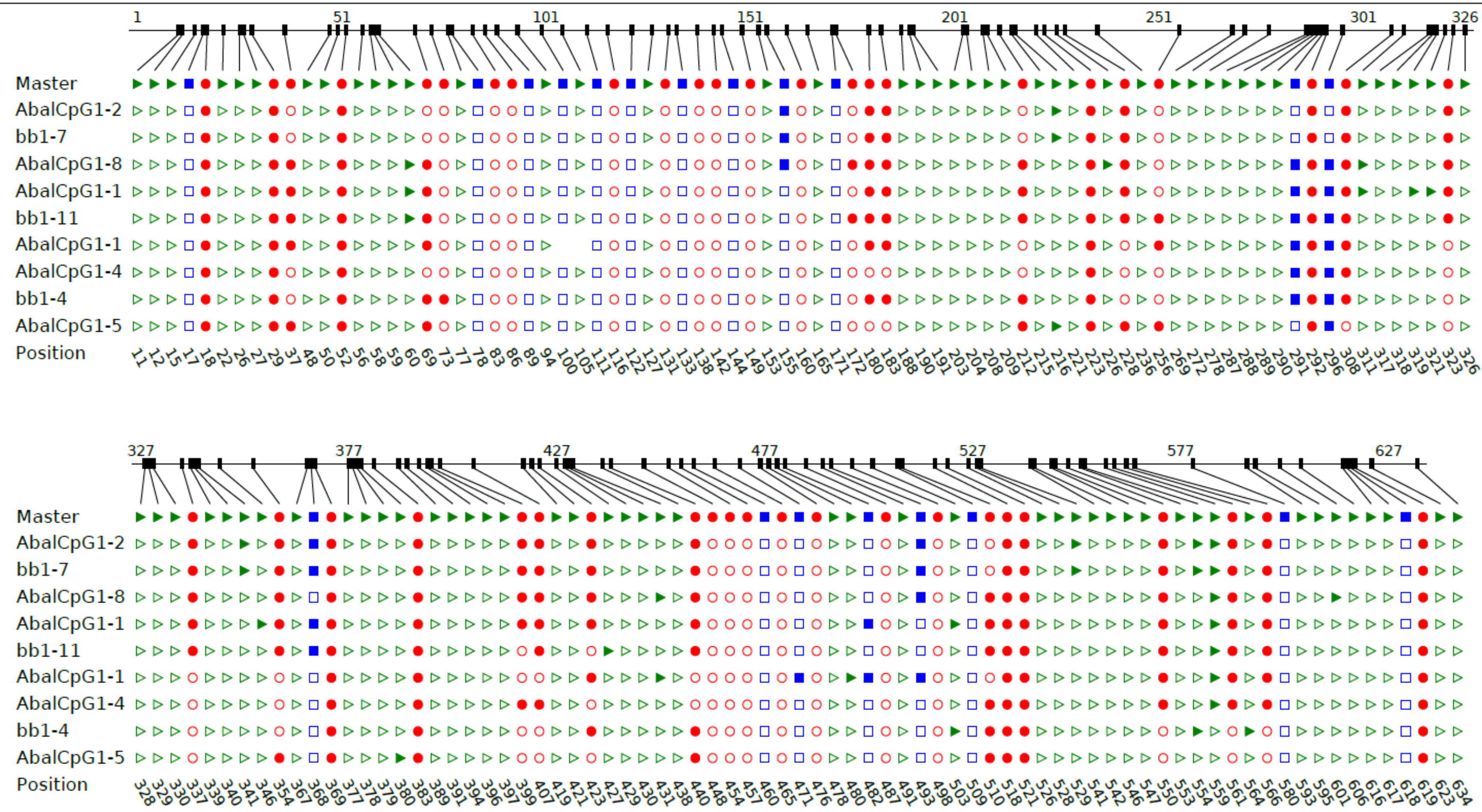

# *A. baldensis* CpG1 island_D subgenome

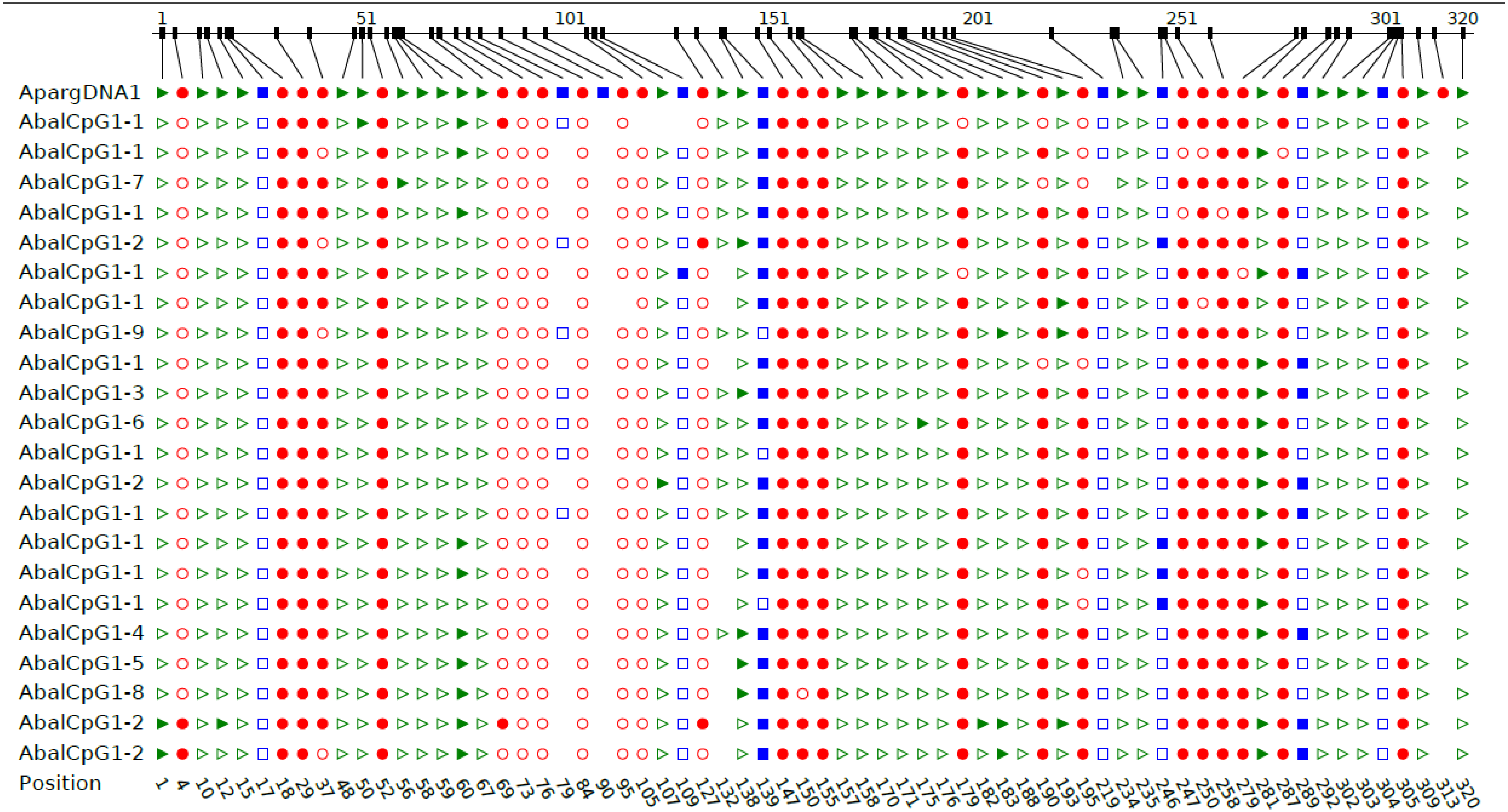

# *A. baldensis* CpG2 island_ A subgenome

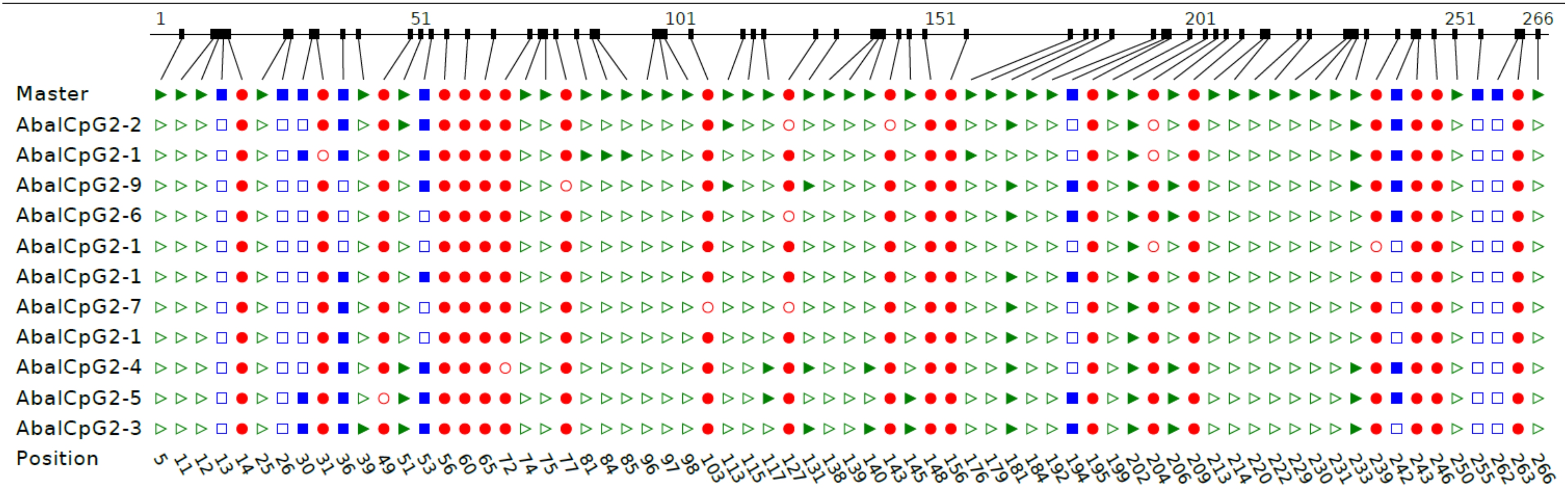

# *A. baldensis* CpG2 island_ D subgenome

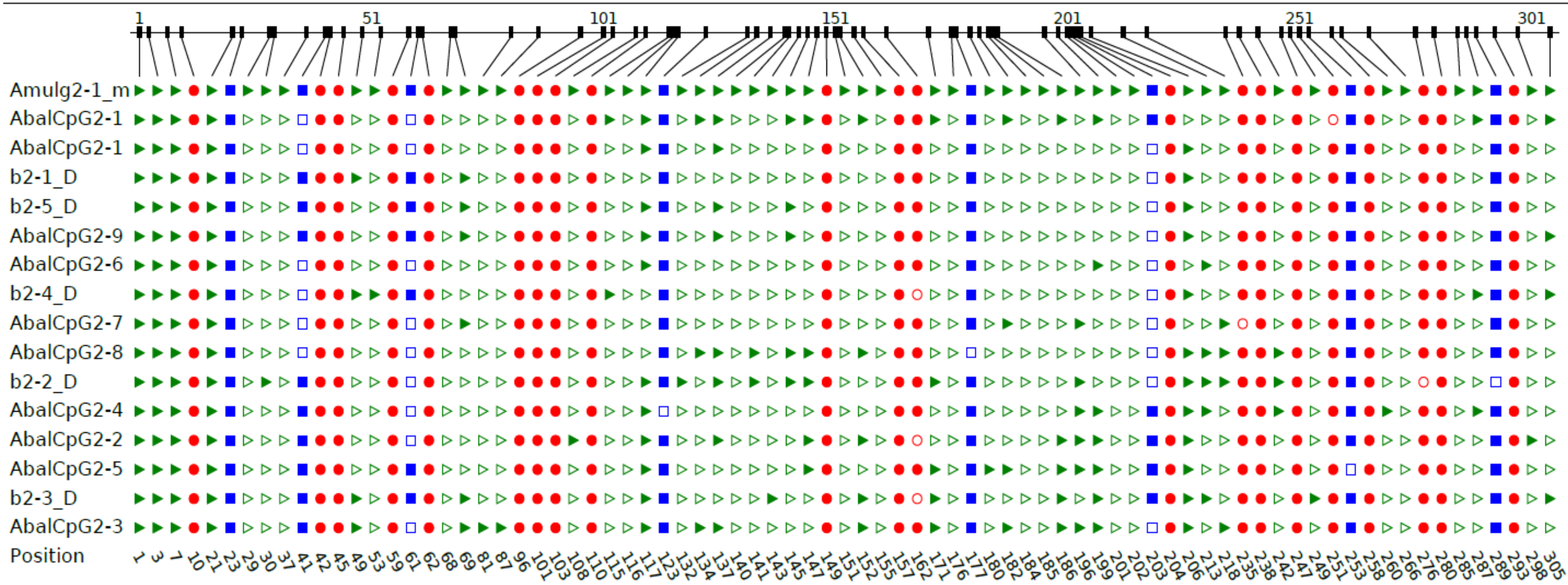

# *A. baldensis* CpG3 island_ A subgenome

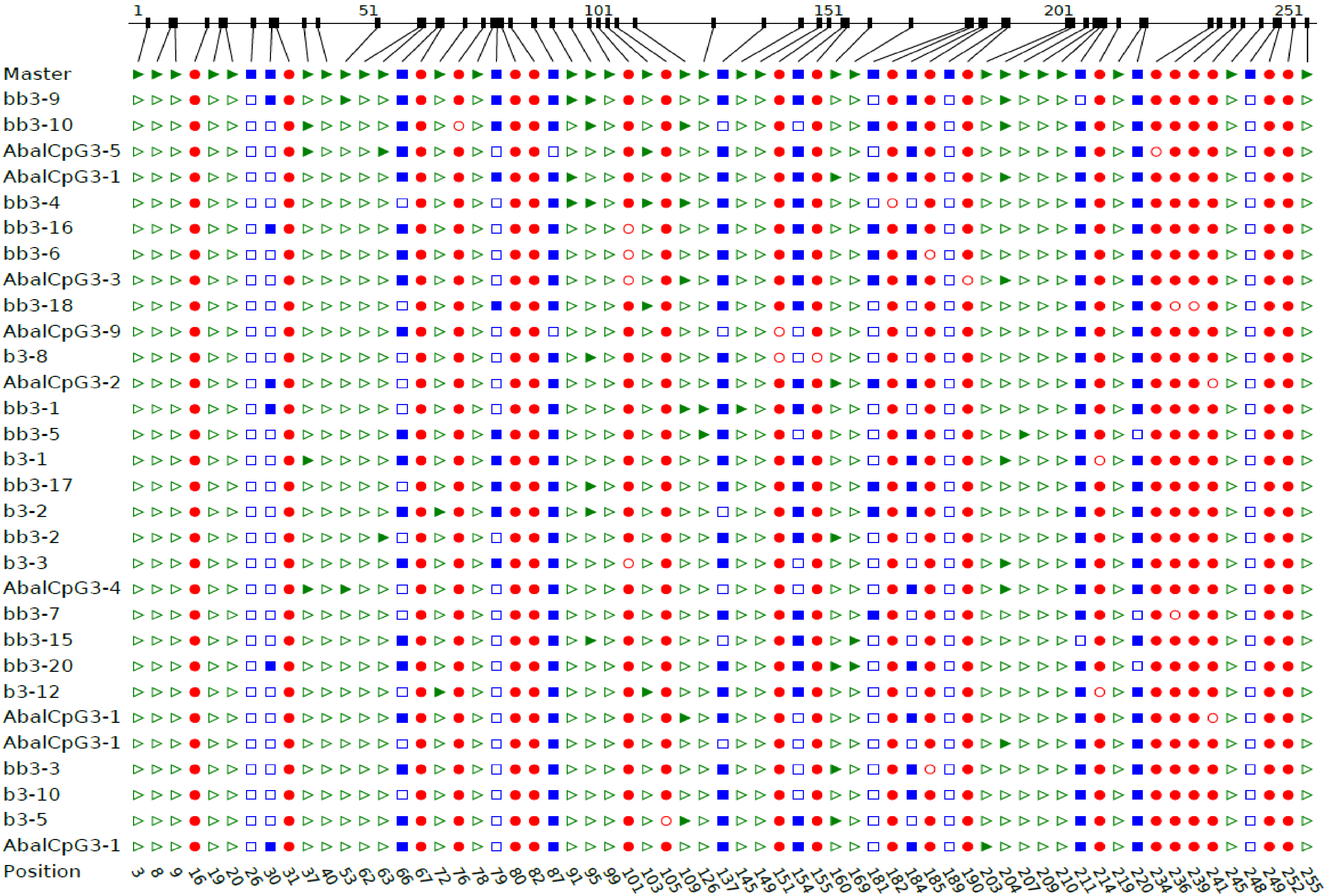

# *A. baldensis* CpG3 island_ D subgenome

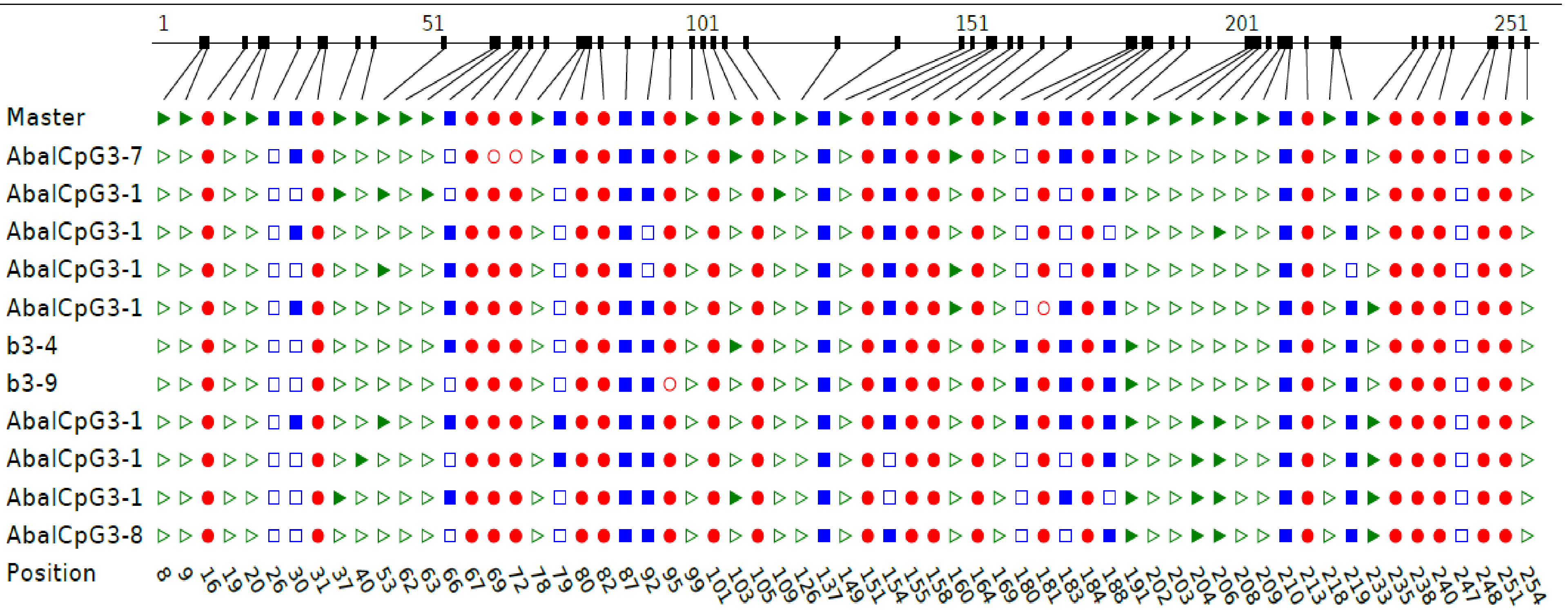

# *A. multifida* CpG1 island_ D subgenome

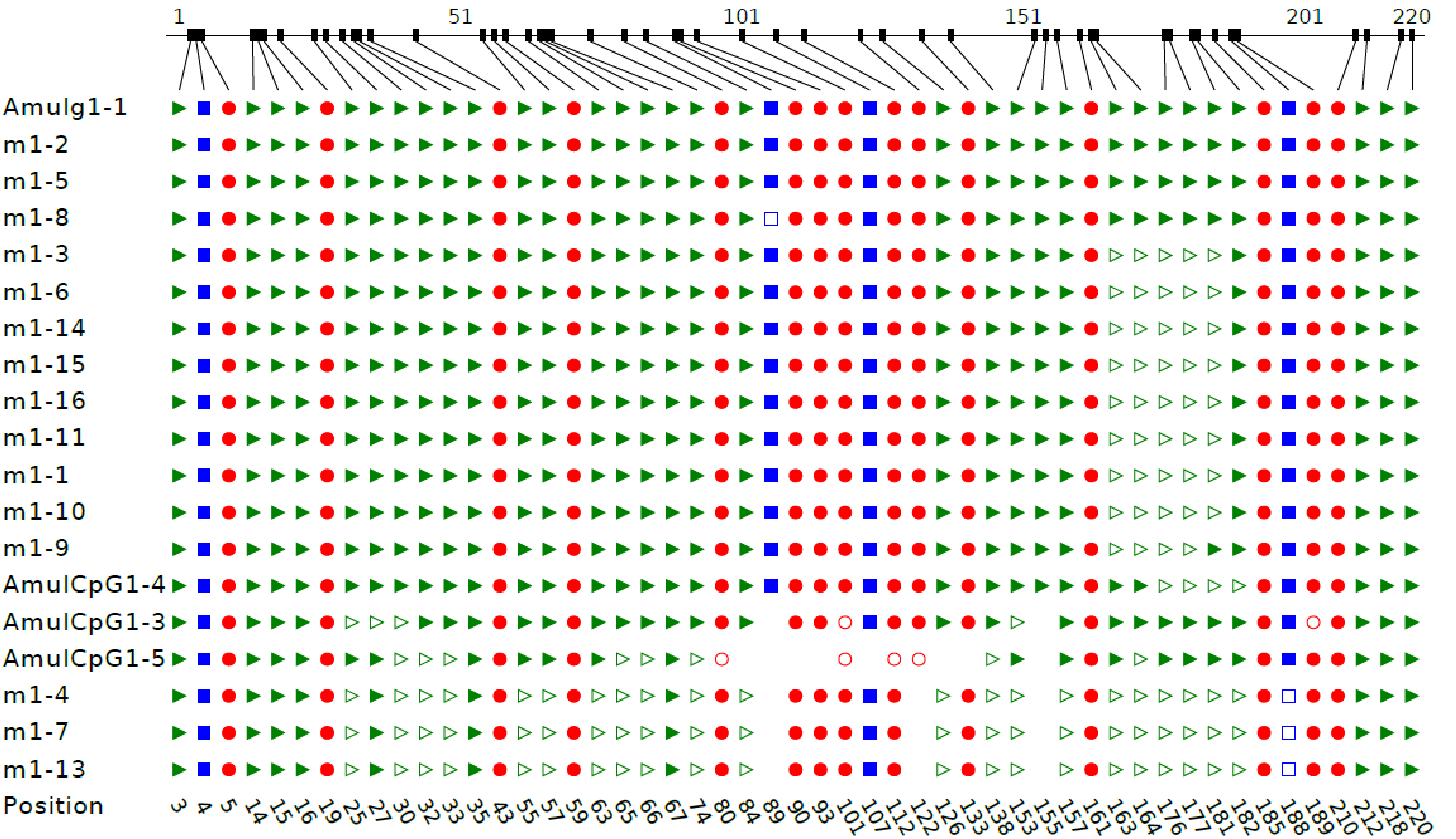

# *A. multifida* CpG1 island_D subgenome_highly methylated clones

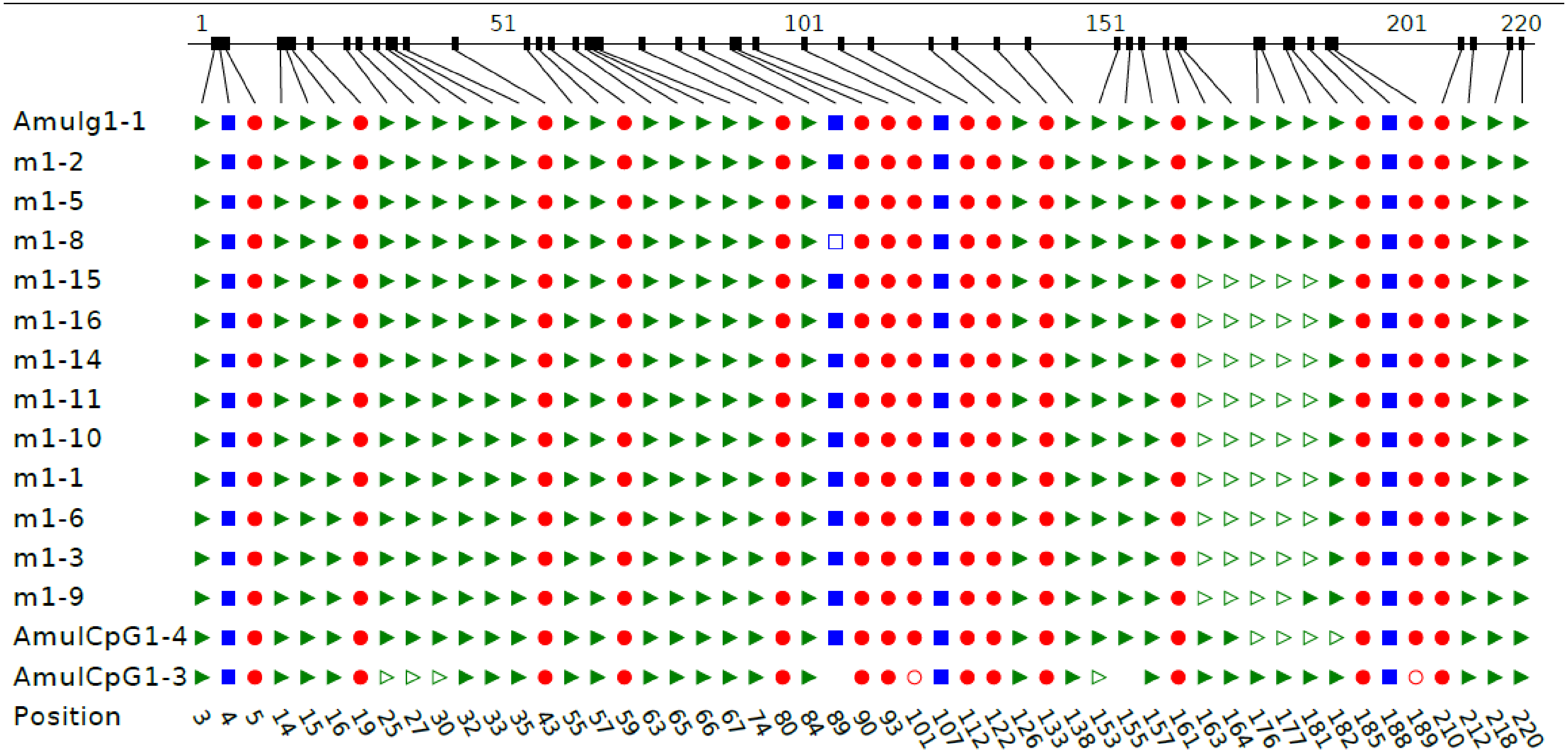


# *A. multifida* CpG1 island_D subgenome_moderately methylated clones

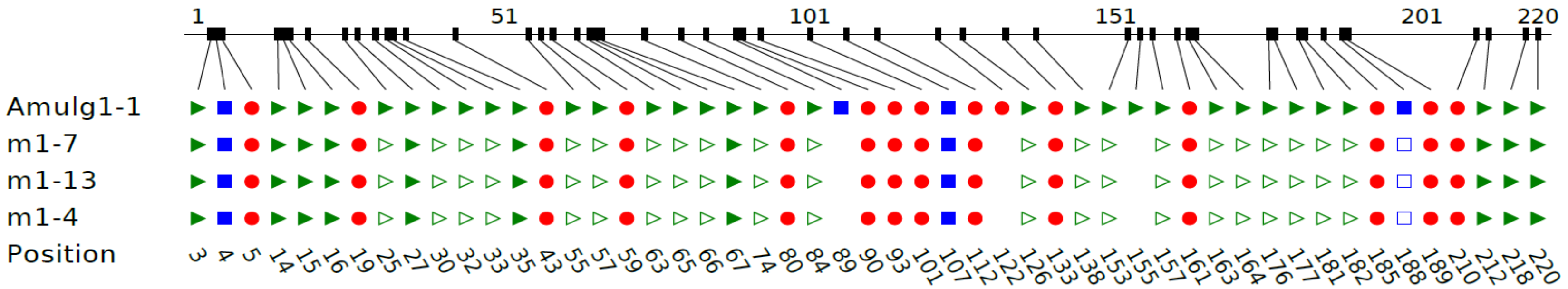

# *A. multifida* CpG2 island_ D subgenome

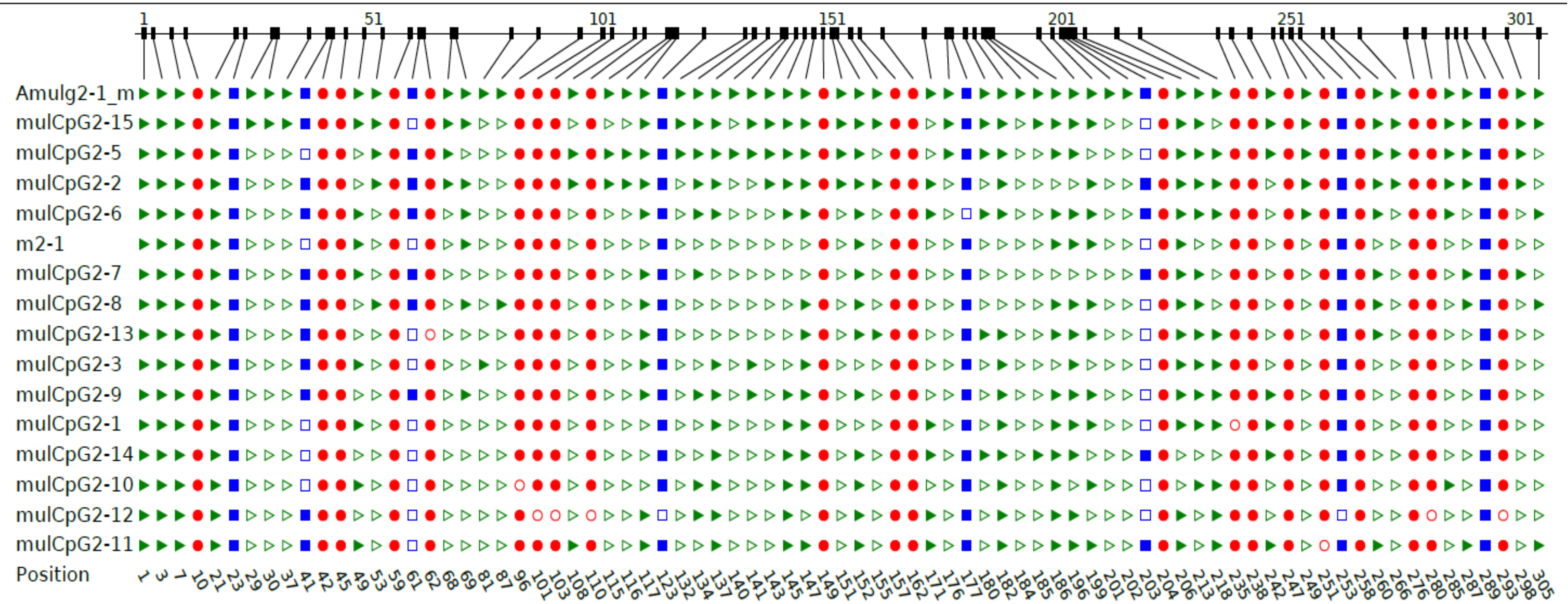

# *A. multifida* CpG3 island_ D subgenome

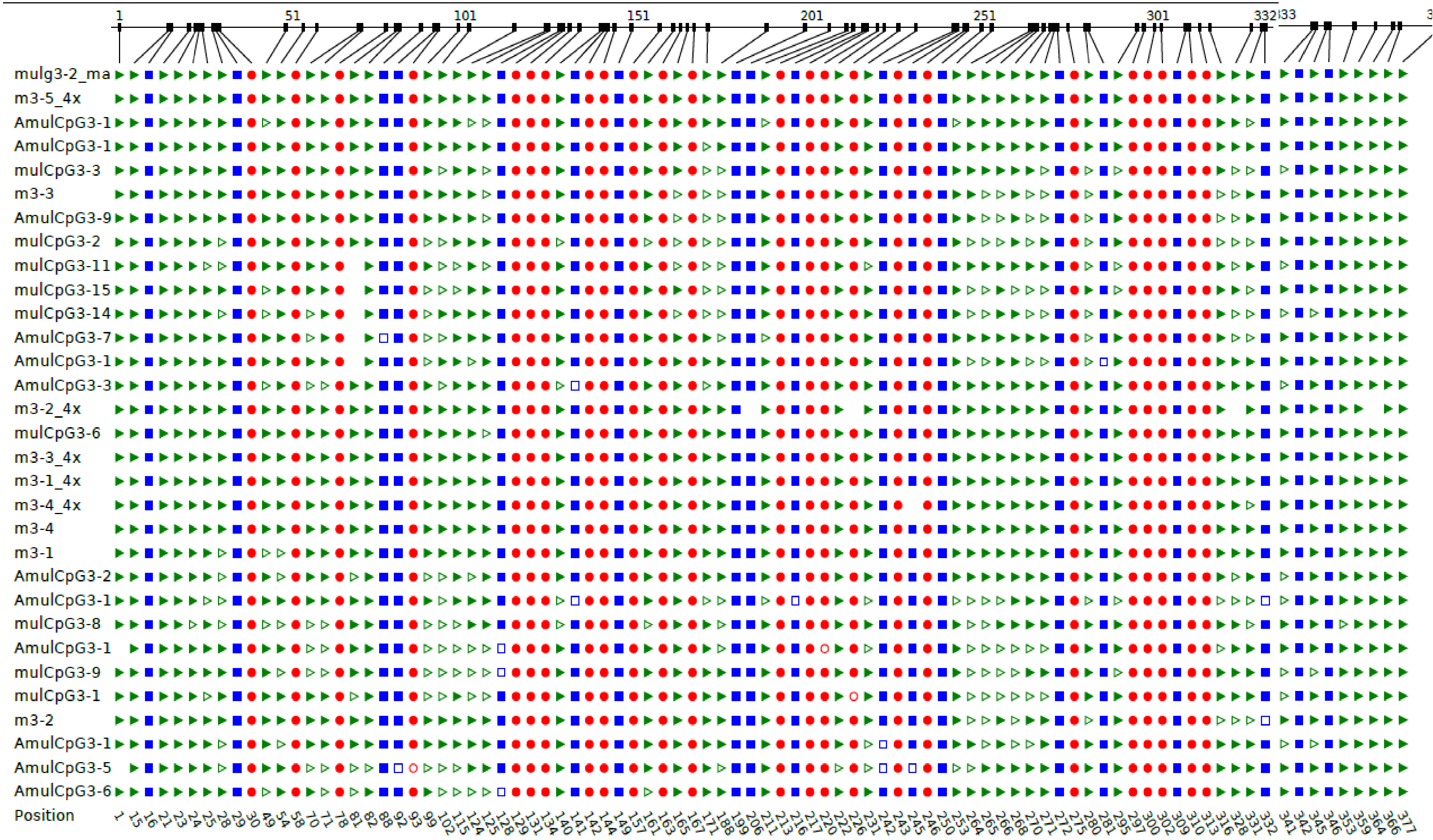

# *A. multifida* CpG3 island_ D subgenome

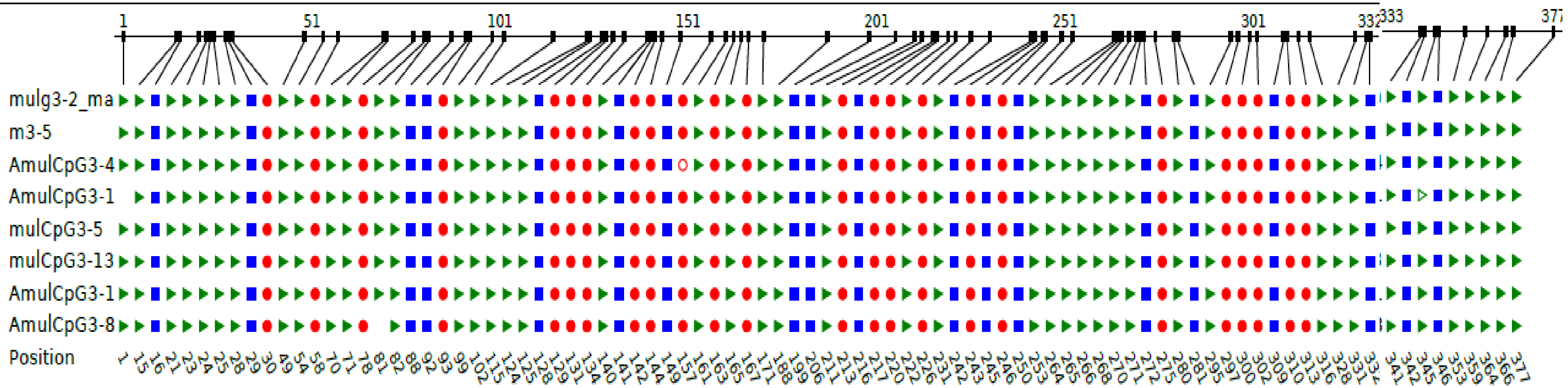

Supplement: Supplementary file 6 [file Image_6.pdf]
