# Supplementary material for: Structure and Methylation of 35S rDNA in Allopolyploids Anemone multifida (2n = 4x = 32, BBDD) and Anemone baldensis (2n = 6x = 48, AABBDD) and Their Parental Species Show Evidence of Nucleolar Dominance
Source: Front Plant Sci. 2022 Jul 6;13:908218. doi: 10.3389/fpls.2022.908218 (PMC9296772; doi:10.3389/fpls.2022.908218)
Supplement: Supplementary file 7 [file Image_7.pdf]

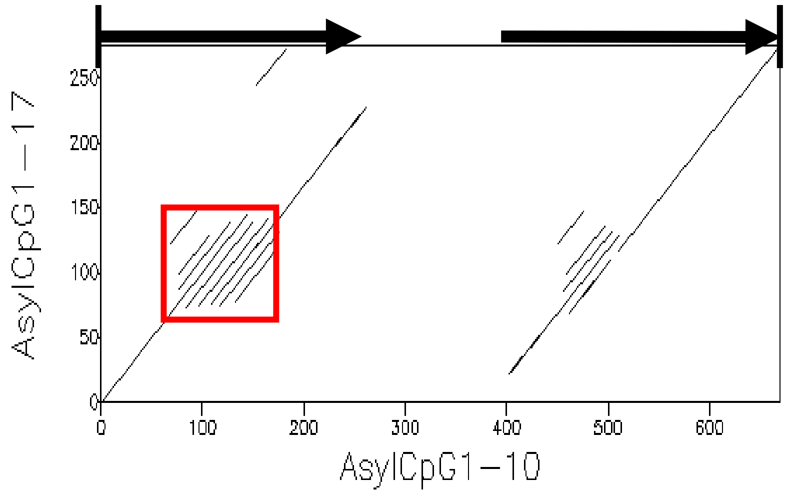

WS= 25, T= 70

Supplementary Figure S7. Presence of size variants of CpG1 region within 5’ETS in Anemone. Dot plot of the clone AsylCpG1-10 belonging to longer size variant of CpG1-637, that shows similarity with the clone AsylCpG1-17 belonging to shorter size variant of CpG1-277 from A. sylvestris, revealing subrepeats of AsylCpG1-17 within AsylCpG1-10 (arrows).
